# Effective reconstruction of functional organotypic kidney spheroid for *in vitro* nephrotoxicity studies

**DOI:** 10.1038/s41598-019-53855-2

**Published:** 2019-11-26

**Authors:** Hyun Mi Kang, Jung Hwa Lim, Kyung Hee Noh, Dongmin Park, Hyun-Soo Cho, Katalin Susztak, Cho-Rok Jung

**Affiliations:** 1 Laboratory of Disease Modeling and Therapeutics, Korea Research Institute of Bioscience and Biotechnology, Daejeon, Republic of Korea; 2 Stem Cell Research Center, Korea Research Institute of Bioscience and Biotechnology, Daejeon, Republic of Korea; 3 Division of Nephrology, Department of Medicine, Department of Genetics, Perelman School of Medicine, University of Pennsylvania, Philadelphia, PA USA; 4 Department of Functional Genomics, Korea University of Science and Technology, Daejeon, Republic of Korea

**Keywords:** Cell death, Phenotypic screening

## Abstract

Stable and reproducible kidney cellular models could accelerate our understanding of diseases, help therapeutics development, and improve nephrotoxicity screenings. Generation of a reproducible *in vitro* kidney models has been challenging owing to the cellular heterogeneity and structural complexity of the kidney. We generated mixed immortalized cell lines that stably maintained their characteristic expression of renal epithelial progenitor markers for the different lineages of kidney cellular compartments *via* the BMP7 signaling pathway from a mouse and a human whole kidney. These cells were used to generate functional and matured kidney spheroids containing multiple renal lineages, such as the proximal tubule, loop of Henle, distal tubules, and podocytes, using extracellular matrix and physiological force, named spheroid-forming unit (SFU). They expressed all apical and basolateral transporters that are important for drug metabolism and displayed key functional aspects of the proximal tubule, including protein endocytosis and increased gamma-glutamyltransferase activity, and cyclic AMP responded to external cues, such as parathyroid hormone. Following exposure, cells fluxed and took up drugs *via* proximal tubule-specific apical or basolateral transporters, and displayed increased cell death and expression of renal injury marker. Here, we developed a new differentiation method to generate kidney spheroids that structurally recapitulate important features of the kidney effectively and reproducibly using mixed immortalized renal cells, and showed their application for renal toxicity studies.

## Introduction

The kidney is a target organ of the toxicity of several xenobiotics and anticancer drugs. Renal toxicity is one of the most frequent adverse events encountered in drug development. In preclinical studies, kidney damage accounts for only 2% of drug development failure, but rises to approximately 20% at Phase 3 and post-approval stages^1–3^. This dramatic increase highlights the limitations of current *in vitro* nephrotoxicity models. Multiple factors contribute to nephrotoxicity, including direct tubular cell toxicity, inflammatory response, crystal precipitation, and hemodynamic effect^4,5^. The proximal tubule is the most common site of drug-induced kidney injury. Drug concentration is the highest in this segment owing to filtration, and most drugs undergo transporter-mediated active secretion, reabsorption, and metabolism at this segment^6,7^. This segment also has a high-energy demand, rendering it susceptible to cellular injury, death, dedifferentiation, and ultimately renal failure^8^. Therefore, to obtain critical information on cellular damage in *in vitro* nephrotoxicity studies, adequate, reproducible *in vitro* models are required to study either the mechanisms underlying the toxic effects of nephrotoxicants or therapeutic approaches in cancer treatment.

Several *in vitro* cellular models have been developed and used in nephrotoxicity evaluations, and past efforts have focused on using human embryonic kidney 293, porcine kidney, human kidney-2 (HK-2), and human telomerase reverse transcriptase (hTERT1)-immortalized renal proximal tubule epithelial cell lines (hPTECs) to test drug-induced toxicity^9–14^. Most cultured cells, such as HK-2 cells, which are a well-known human proximal tubule cell line, do not express critical uptake transporters, such as organic anion and cation transporters. The expression of apical efflux transporters (P-gp, MRPs) is much lower in most cultured cells than in the human kidney cortex^15^.hPTECs express the relevant transporters at both the mRNA and protein levels^16^, but functional activity assays of transporters on hPTECs have not been successfully performed^1^. Furthermore, immortalized cell lines are less sensitive or insensitive to well-known nephrotoxicants, than primary human renal proximal tubular cells^7,15^. More recently, human-induced pluripotent stem cell (iPSC)-derived renal organoids have been developed^17,18^. Kidney organoids contain self-organized nephron-like structures composed of early podocyte cells connected to tubular structure, and they display proximal tubule functions, such as dextran uptake, and response to nephrotoxicants^17,18^. Although the iPSC-derived organoid system is widely popular, recent data showed that this system generates a highly heterogeneous population of cells^19^, inducing variable amount of immature cells and non-renal cell types. Moreover, this organoid culture system usually requires several weeks with multi step-protocol to generate matured organoids that mimic the *in vivo* development.

Here, we report a simple, efficient, and highly reproducible system to generate matured and functional spheroids using established renal primary cell lines. These cells in our culture system showed progenitor-like characteristics and maintained their original renal tubule cell characteristics by activating the BMP7 pathway, which is secreted by the proximal tubule, loop of Henle, and distal tubule. Moreover, they successfully differentiated into functional kidney spheroids with a simple method within seven days, expressed various basolateral and apical transporters, and responded to nephrotoxic drugs depending on the activities of specific uptake and efflux transporters.

## Results

### Mixed immortalized cells possessed progenitor-like characteristics and retained cellular heterogeneity of the kidney

**Figure 1:**
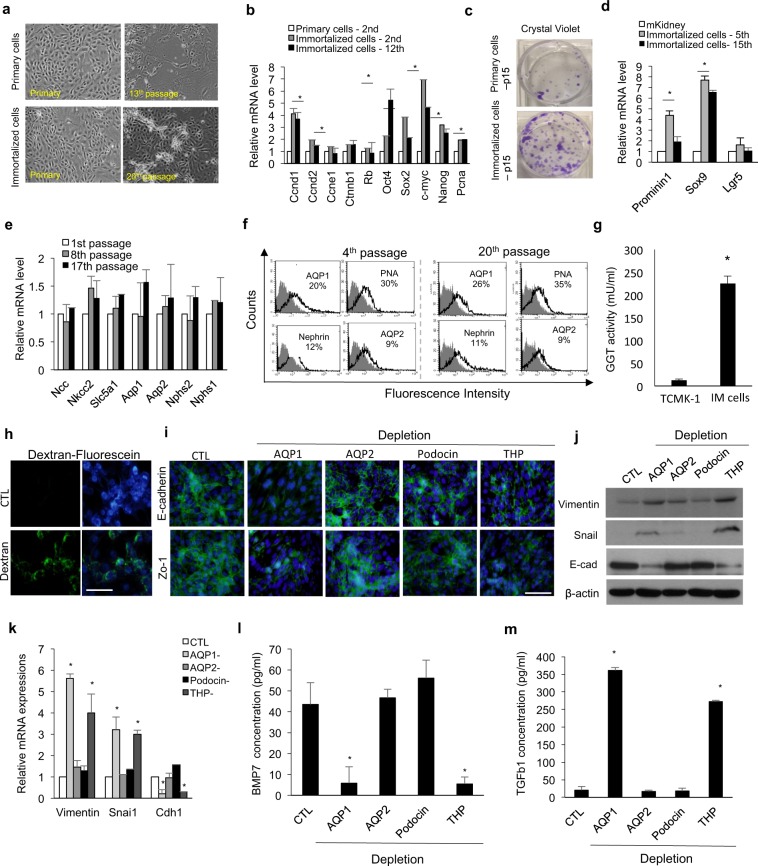
Establishment and characterization of primary renal cell lines from mouse. (**a**) Morphology of mouse tubular epithelial cells during *in vitro* culture. (**b**) Quantitative RT-PCR analysis of proliferation markers related to or downstream targets of hTERT and SV40 gene. n = 4 for primary cells at the 2^nd^ passage, immortalized cells at the 2^nd^ passage, and immortalized cells at the 12^th^ passage, respectively. (**c**) Crystal violet staining of primary cells and immortalized cells after the 15^th^ passage. (**d**) Relative mRNA expression of Prominin1, Sox9, and Lgr5 in the immortalized cells at the 5^th^ and 15^th^ passages compared to that in mouse kidney lysate (mKidney). n = 4 for immortalized cells at the 5^th^ and 15^th^ passages, respectively, and n = 3 for mouse kidney lysates. (**e**) Quantitative RT-PCR analysis of kidney-specific segment markers in mouse cells at the 1^st^, 8^th^, and 17^th^ passages. n = 5 for 1^st^, 8^th^, 17^th^ passage immortalized cells, respectively. (**f**) FACS analysis of tubule segment markers in cells at the 4^th^ and 20^th^ passages. Immortalized cells at the 4^th^ and 20^th^ passages were stained with anti-AQP1, PNA, nephrin, or AQP2 (open histograms) or isotype control mAb (grey histograms). (**g**) GGT activity of mouse immortalized primary cells compared to a mouse tubular cell line (TCMK-1). n = 6 for TCMK-1 and mouse renal primary cells, respectively. (**h**) Dextran uptake by mouse immortalized primary cells at the 8^th^ passage after 48 h of incubation with Alexa Fluor 488-conjugated dextran. (**i**) Representative images of immunostaining for E-cadherin and Zo-1 in CTL and AQP1-, AQP2-, podocin-, or THP-depleted cells. Cells were at the 5–7^th^ passages and the depleted population was obtained from negative fractions via magnetic activated cell sorting. (**j,k**) Western blotting (**j**) and quantitative RT-PCR (**k**) analyses for epithelial-mesenchymal transition of cells of the CTL and AQP1-, AQP2-, podocin-, or THP-depleted population. The grouping of gels/blots cropped from different parts of the same gel. (**l,m**) The amounts of BMP7 (**l**) and TGFb1 (**m**) secreted by cells of the CTL and AQP1-, AQP2-, podocin-, or THP-depleted population. n = 4 for CTL and AQP1-, AQP2-, podocin-, or THP-depleted cells, respectively. All data are shown as the means ± s.e.m. *p < 0.05 compared with primary cells, TCMK-1 or CTL by unpaired Student’s *t*-test. Scale bar, 20 μm.

We aimed to generate a kidney cell line that could be reproducible and easily differentiated using a simple protocol. To obtain cells that maintain their original characteristics with proliferative potential, we immortalized the cells using hTERT and simian virus 40 large T (SV40-T) (Fig. S1a–c). Immortalized cells maintained epithelial cell morphology during *ex vivo* expansion (Fig. 1a), and they underwent an average of 144.5 doublings over 30 passages, while primary cells without immortalization underwent an average of 55.6 doublings (Fig. S1d). The immortalized cells expressed markers of proliferation such as *cyclin D1*, *D2*, *Pcna*, *c-myc*, and *Nanog* (Fig. 1b). Our new cell lines showed higher clonal expansion capacity after two weeks of culture than did primary cells (Fig. 1c). The transcript levels of renal progenitor cell markers (*SRY-box* 9 and *prominin* 1) were 4–7 fold higher in immortalized cells than in mouse kidney lysates (mKidney), indicating that this progenitor-like cell line had epithelial characteristics (Fig. 1d). On the contrary, there were no significant differences in the expression of common adult stem cell markers, such as *KIT ligand*, *Cd90*, and *Cd105*, between immortalized cells and mouse kidney lysates (mKidney; Fig. S1e). In contrast, primary cells without immortalization changed from epithelial-like to fibroblast-like after three passages, and showed increased expression of epithelial-mesenchymal transition (EMT) markers (Fig. S2a,b). Additionally, the expression of kidney-specific markers, such as aquaporin 1 (AQP1) (a proximal tubule cell marker), gradually declined (Fig. S2c). To better characterize the stability of the immortalized cells, we examined the mRNA and protein expression patterns of these cells at early-, middle-, and late-passage. Quantitative reverse transcription-polymerase chain reaction (qRT-PCR) results showed that immortalized cells maintained the expression of kidney-specific markers in cell culture (Fig. 1e). The expression of AQP1, peanut agglutinin (PNA, a loop of Henle/distal tubule marker), nephrin (glomerular cell marker), and aquaporin 2 (AQP2, a collecting duct marker) was also detected in cells at the 4^th^ and 20^th^ passages (Fig. 1f). Since we established the cell line from whole kidney lysates, which were mixed and heterogeneous cell populations, we investigated multiple renal epithelial progenitors present in immortalized cells at early (4^th^ passage) and late (16^th^ passage) passages to define the origin of these mature kidney cells during the culture periods. Approximately 30‒34% of the total cell population were CD133- or Sox9-positive, and 12% expressed Hoxb7 at the 4^th^ passage (Fig. S3a,b). This proportion was maintained at the late passage (16^th^ passage) as well, and could be supplied to mature tubule cells throughout the culture period. The cell line showed proximal tubule-specific functional properties, such as gamma-glutamyltranspeptidase (GGT) activity (Fig. 1g) and fluorescein-conjugated dextran uptake (Fig. 1h), compared with a mouse proximal tubule cell line (TCMK-1 cells). To identify the factors maintaining renal epithelial characteristics, we obtained AQP1-, AQP2-, Tamm–Horsfall protein (THP)-, or podocin-depleted population from these cell lines using magnetic activated cell sorting and maintained them as used for immortalized cell maintenance. We examined their gene and protein levels to confirm their depletion in target cells (Fig. S4a–c). AQP1- or THP-depleted populations showed a loss of tight junctions with decreased expression of E-cadherin and Zo-1 (Fig. 1i,j), and they entered the EMT process by increasing the expression of vimentin and snai1 (Fig. 1j,k). BMP7 has been reported to maintain renal epithelial-like characteristics and inhibit EMT transition by TGFβ1, and its administration improves kidney function recovery^20,21^. Therefore, we investigated the expression and secretion of BMP7 and TGFβ1 in the depleted populations. BMP7 secretion by AQP1- or THP-depleted population significantly decreased (Fig. 1l), whereas that by TGFβ1 significantly increased, compared with that by the CTL and AQP2- or podocin-depleted populations (Fig. 1m). Therefore, BMP7 secretion by AQP- and THP-positive cells may be important to maintain renal tubular epithelial cells and prevent their entry into the EMT process. Next, we established an immortalized human renal cell line from a human kidney tissue using the same protocol. Similar to the mouse cell line, the human renal cells maintained their epithelial morphology (Fig. S5a) and expressed all segment markers and proteins similarly to the primary cells (Fig. S5b,c). Moreover, they showed increased GGT activity, compared with hPTECs (Fig. S5d), and fluorescein-conjugated dextran uptake (Fig. S5e). Collectively, these results suggested that the cell line we generated contained a heterogeneous progenitor population for the different compartments of the kidney, and it can be cultured while maintaining original renal characteristics throughout the *in vitro* culture period.

### Functional kidney spheroids were generated by SFU in the modified extracellular matrix via shear stress-related signaling pathway

**Figure 2:**
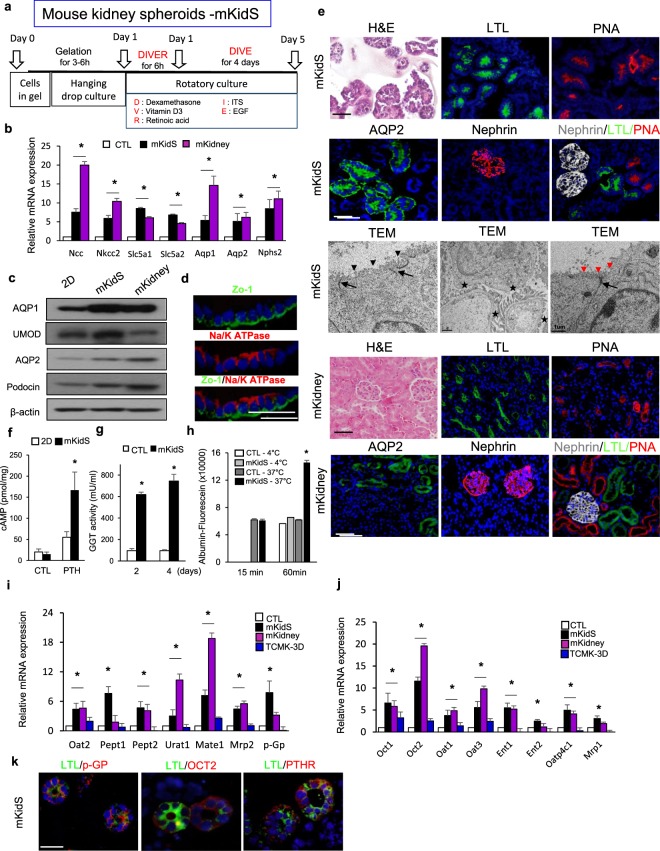
Generation of functional kidney spheroids using immortalized mouse primary cells. (**a**) Schematic diagram of SFU protocol (combination of hanging drop and rotation) and maturation factors for generation of kidney spheroids for structural maturation. (**b**) Quantitative RT-PCR analysis of kidney-specific segment markers in mouse kidney spheroids (mKidS) compared with 2D controls (2D CTL) and mouse kidney (mKidney). n = 9 for 2D CTL and mKidS, n = 3 for mKidney. (**c**) Western blotting analysis for renal multi-lineage markers in mouse kidney spheroids compared with 2D CTL and mKidney. The grouping of gels/blots cropped from different parts of the same gel. (**d**) Immunostaining images for cell polarity of Zo-1, an apical membrane, and Na/K+ ATPase, a basolateral membrane marker in mKidS. Scale bar, 10 μm. (**e**) Representative images of mKidS at day 5 of maturation stained with hematoxylin and eosin (H&E) or immunostained for the kidney-specific markers LTL, PNA, AQP2, and nephrin; transmission electron microscopy of kidney spheroids displaying long (black arrowhead) and short (red arrowhead) villi with tight junctions (black arrow) and foot processes (asterisks in the middle panel). Scale bar of TEM image, 1 μm. Mouse kidney from 12 weeks-old mice was used as a positive control for immunostaining of mKidS. (**f**) Response to parathyroid hormone (PTH) compared with 2D cells and 3D control spheroids (without maturation factors, CTL). n = 6 for 2D CTL, 2D PTH treated, n = 10 for 3D CTL and mKidS. (**g**) GGT activity in mKidS compared to 3D CTL after 2 and 4 days of maturation. n = 8 for CTL and mKidS, respectively. (**h**) Temperature-dependent albumin-fluorescein uptake compare to 3D CTL. n = 12 for 3D CTL and mKidS, respectively. (**I,j**) Relative mRNA expression of apical (**i**) and basolateral (**j**) transporters in mKidS compared with 2D cultured cells, TCMK-1 spheroids generated using the same protocol for mKidS (TCMK-3D) and mKidney. n = 8 for 2D CTL and mKidS and n = 4 for TCMK-3D. (**k**) Representative images of mKidS immunostained for p-GP, OCT2, and PTHR. All data are shown as the means ± s.e.m. *p < 0.05 compared with 2D or 3D control by unpaired Student’s *t*-test. Scale bar, 20 μm.

To generate *in vitro* kidney spheroids, we used an SFU protocol combining hanging-drop seeding and rotation, as previously described^22^. Cell aggregates cultured by the SFU methods (HD/Rotation) showed significantly increased expression of kidney-specific markers for the proximal, distal tubules, loop of Henle, collecting duct, and podocytes (Fig. S6a). Transporters play critical roles in small-molecule uptake and secretions and responses to nephrotoxicants in the kidney. We evaluated the mRNA expression levels of uptake and efflux drug transporters in the apical and basolateral membranes of cell aggregates generated by HD/Rotation, compared to those in 2D cultured cells. Some transporters, such as *Oct1, Oct2, Oat1*, and *Oat3,* in the basolateral membrane were increased in SFU-generated cell aggregates compared to those in 2D cultured cells, whereas there were no differences in drug transporter expression in the apical membrane, except for *Oat2* and *Pept1* (Fig. S6b,c). Moreover, *Ent2, Oatp4c1*, and *Mrp1* expression in the basolateral membrane, as well as *Urat1* expression in the apical membrane significantly decreased in SFU-generated cell aggregates compared to that in 2D cultured cells (Fig. S6b,c). It has been proposed that matrices such as ECM gel and Matrigel improve the maturation of pluripotent stem cells into organoids^23^. We embedded our cells in ECM gel (Sigma) or Matrigel to examine their effects on the induction of functional and structural maturation of kidney spheroids. We observed similar tubular and glomerular structures in the kidney spheroids embedded in the mixture of ECM gel and Matrigel, whereas the cells in the ECM or Matrigel formed small-size tubules (Fig. S6d). The cells embedded in matrix grew well, forming colonies in the gel (kidney spheroids, Fig. S6e). We also examined kidney tubule- and glomeruli-specific gene expression patterns in kidney spheroids embedded in Matrigel, ECM gel, or mixed gel. The expression of kidney-specific differentiation markers increased in the cells cultured in the mixture of ECM and Matrigel, compared to those in cells cultured in either Matrigel or ECM gel (Fig. S6f). We investigated the effects of several factors known to be involved in renal tubule differentiation and maturation, such as dexamethasone, ITS, vitamin D3, and retinoic acid. The apical and basolateral transporter expression profiles showed that the combination of dexamethasone, ITS, vitamin D3, and EGF after short exposure to retinoic acid (DIVER-DIVE) was the best condition for tubular maturation (Fig. S6g,h). Thus, we concluded that the ECM gel and Matrigel mixture, as well as the medium supplemented with dexamethasone, ITS, EGF, vitamin D3, and retinoic acid provided the best conditions for spheroid maturation, and these conditions were used for further experiments (Fig. 2a). Kidney spheroids showed increased expression levels of genes and proteins related to various kidney tubule segments compared with those in 2D cells, and similar expression of AQP1 and UMOD to that in mouse kidney lysates (Fig. 2b,c). Moreover, confocal microscopic image analysis of these matured cells revealed normal epithelial cell polarity, as indicated by the continuous linear distribution of tight junction protein of the apical membrane, Zo-1, and restriction of Na/K ATPase to the basolateral membrane (Fig. 2d). They also showed positive results for Lotus Tetragonolobus Lectin (LTL, proximal tubule marker), PNA, AQP2, and nephrin (podocyte marker) (Fig. 2e, upper panel), without co-expression of proximal tubule, Loop of Henle/distal tubule, and glomerular markers (Fig. 2e, upper panel). Mouse kidney from 12 weeks-old mice was used as positive control for immunostaining of mouse kidney spheroids, and staining patterns of mouse kidney spheroid were similar to those of mouse kidney (Fig. 2e, down panel). Transmission electron microscopy showed distinct epithelial subtypes; typical proximal tubular epithelium displaying long microvilli with tight junctions (left panel), cells with few short microvilli surrounding an open lumen characteristic of collecting ducts/distal tubules (right panel), and the presence of foot processes (middle panel) characteristic of podocytes (Fig. 2e, middle panel). These results suggested that the kidney spheroid we generated possessed the distinct segments of the kidney compartments similar to mouse kidney. To perform a comprehensive and unbiased expression analysis on these cells, we performed RNA-seq analysis on kidney spheroids. This analysis identified 1,348 transcripts with a corrected *P*-value of 0.05 and at least a 50% change in their expression values (Supplemental Table II) compared to 2D cells. Gene set enrichment analysis highlighted specific changes in metabolism-related pathways, such as glutathione metabolism and sulfur compound metabolism, and responses to toxic substances related to the functions of the proximal tubules (Fig. S7a–c), compared to 2D cells. The expression levels of these genes were equivalent to those observed in mouse kidneys (Supplemental Table II). These results showed that kidney spheroids produced in a 3D environment with SFU and a scaffold contained all the anticipated nephron components in structurally mature states, and not just cell aggregates.

Multiple previously published organoid systems can recapitulate fetal gene expression and structural changes; however, little is known about how well these organoids can functionally recapitulate the kidney. The use of kidney spheroids in drug screening requires functional maturation of nephrons. We focused on the proximal tubules, which play important roles in solute, vitamin, hormone, and amino acid reabsorption. Functionally, the proximal tubules are known to respond to parathyroid hormone (PTH) by increasing calcium resorption and phosphate secretion through a cyclic adenosine monophosphate (cAMP)-dependent mechanism^24^. Exposure to PTH leads to an increase in cAMP levels in kidney spheroids, compared with those in control spheroids (SFU-cultured gel spheroids without maturation factors), and in 2D cells, thereby suggesting an intact and mature PTH receptor expression and a cellular response (Fig. 2f). Gamma glutamyl transferase plays a key role in the gamma-glutamyl cycle, a pathway for synthesis and degradation of glutathione, as well as drug and xenobiotic detoxification, a key function of the kidney. GGT activity significantly increased in kidney spheroids compared with that in control spheroids and TCMK cells (Figs 1g and 2g). The spheroids also displayed temperature-dependent uptake of fluorescein-labeled albumin (Fig. 2h). The expression levels of major uptake and efflux drug transporters increased in kidney spheroids, compared with those in 2D cultured cells and TCMK spheroid, as examined using the same culture methods as our kidney spheroids (Fig. 2i,j). Moreover, these expression levels were similar to those in mouse kidney lysates (Fig. 2i,j). Immunostaining showed that p-GP transporter was located in the apical membrane, whereas OCT2 was located in the basolateral membrane of the proximal tubule, and that PTH receptor (PTHR) was expressed on the proximal tubule (Fig. 2k). These data suggested that the mouse kidney spheroids were functionally and structurally mature.

Shear stress induces reorganization of renal proximal tubule cells and enhances their tight junction^25^, whereas fluid shear stress promotes cellular differentiation in various cells^26–28^. To define the mechanism of the effect of SFU on kidney spheroid maturation, we examined ER stress-related gene expression under shear stress (Fig. S8a,b). Xbp-1, Atf4, and at6 showed increased expression after 1 h of rotatory culture, followed by increased expression of transcription factors related to renal progenitors (Fig. S8c) and mature lineage markers at the late time point of rotatory culture (Fig. S8d). *XBP-1* regulated the expression of *Wt1* (renal progenitor marker), since *XBP-1* motif was predicted within −1 kb of human and mouse *WT1* promoter regions (Fig. S8e). *Wt1* expression induced *Aqp1* expression by transcriptional regulation (Fig. S8f). We also examined the sequential expression of these factors by western blotting analysis (Fig. S8g). Together, these results suggested that increased *Xbp-1* under fluid shear stress by SFU culture condition promoted renal cell maturation *via* *Wt1* (Fig. S8h).

### Functional human kidney spheroid was generated by SFU in the modified extracellular matrix

**Figure 3:**
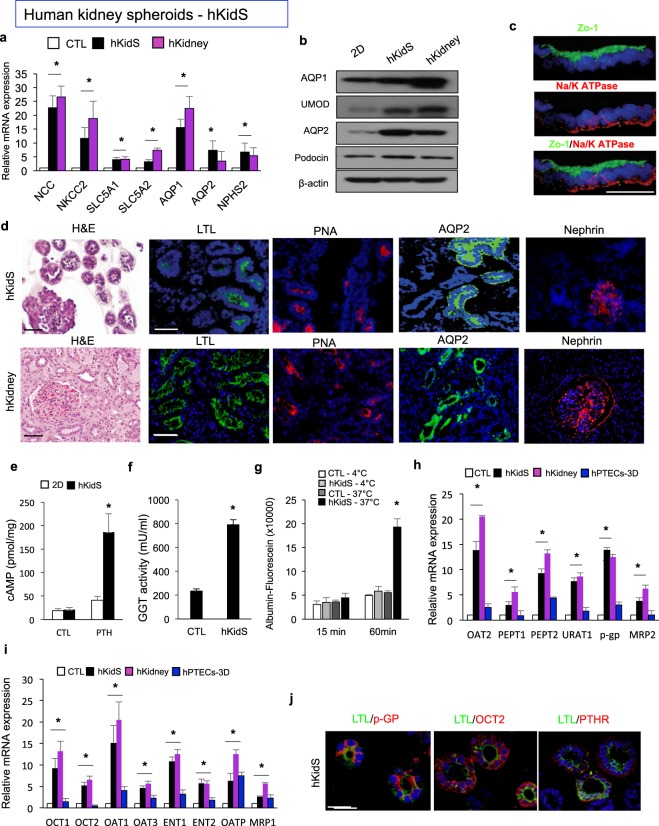
Generation of functional kidney spheroids using immortalized human primary cells. (**a**) Quantitative RT-PCR analysis of kidney-specific segment markers in hKidS compared with 2D controls (2D CTL) and human kidney lysates (hKidney). n = 8 for 2D CTL and hKidS and n = 3 for hKidney. (**b**) Western blotting analysis for renal multi-lineage marker in mouse kidney spheroids compared to 2D CTL and hKidney. The grouping of gels/blots cropped from different parts of the same gel. (**c**) Representative images for cell polarity of Zo-1, an apical membrane, and Na/K ATPase, a basolateral membrane marker in hKidS. Scale bar, 10 μm. (**d**) Representative images of human kidney spheroids (hKidS) at day 5 of maturation stained for the kidney-specific markers LTL, PNA, AQP2, and Nephrin. Human kidney was used as positive control for immunostaining of hKidS. (**e**) Responses to parathyroid hormone (PTH) of hKidS compared with 3D control spheroids (without maturation factors, CTL) and 2D cells. (**f**) GGT activity in hKidS compared with 3D CTL. (**g**) Temperature-dependent albumin-fluorescein uptake of hKidS compared to 3D CTL. n = 10 for 3D CTL and hKidS. (**h,i**) Relative mRNA expression of apical (**h**) and basolateral (**i**) transporters in hKidS compared to 2D cultured cells, hPTECs spheroids generated using the same protocol for hKidS (hPTECs-3D), and hKidney. n = 9 for 2D CTL and hKidS and n = 3 for hPTECs-3D and hKidney. (**j**) Representative images of hKidS immunostained for p-GP, OCT2, and PTHR. All data are shown as the means ± s.e.m. *p < 0.05 compared with 2D or 3D control by unpaired Student’s *t*-test. Scale bar, 20 μm.

We performed the same analysis described above on human kidney spheroids. Human kidney spheroids were positive for various kidney component markers, such as *NCC*, *NKCC2*, *SGLTs*, *AQP1*, *AQP2*, and *Podocin* (Fig. 3a), and they expressed all tubule segment-specific markers similar to those expressed by human kidney lysates (Fig. 3b). Confocal microscopy image analysis showed normal epithelial cell polarity, indicated by a continuous linear distribution of the tight junction proteins of the apical membrane, Zo-1 and E-cadherin, and restriction of Na/K ATPase to the basolateral membrane (Fig. 3c). Immunostaining images further confirmed the multi-lineage marker expression (Fig. 3d); human kidney was used as positive control for immunostaining of human kidney spheroids, and the staining patterns of human kidney spheroid were similar to those of human kidney (Fig. 3d). We also performed functional analyses of the human-derived kidney spheroids. cAMP responses to PTH hormone and GGT activity were similarly higher than those in 2D and control spheroids (Fig. 3e,f**)**. The kidney spheroids also showed temperature-dependent uptake of fluorescein-labeled albumin (Fig. 3g). The expression levels of all major uptake and efflux drug transporters examined in this study increased in spheroids, compared with those in the 2D cultured cells and hPTEC spheroids, as examined using the same culture methods as our kidney spheroids (Fig. 3h,i**)**, and these levels were similar to those observed in human kidney lysates (Fig. 3h,i). pGP and OCT2 proteins were also localized to the apical and basolateral membrane, respectively, and PTHR expression was localized to the proximal tubule (Fig. 3j). These data suggested that human kidney spheroids were functionally and structurally mature, and importantly that this protocol could be applied to both mouse and human cells.

### SFU based-protocol generated matured and functional kidney spheroids that were reproducible and stable during long culture periods

**Figure 4:**
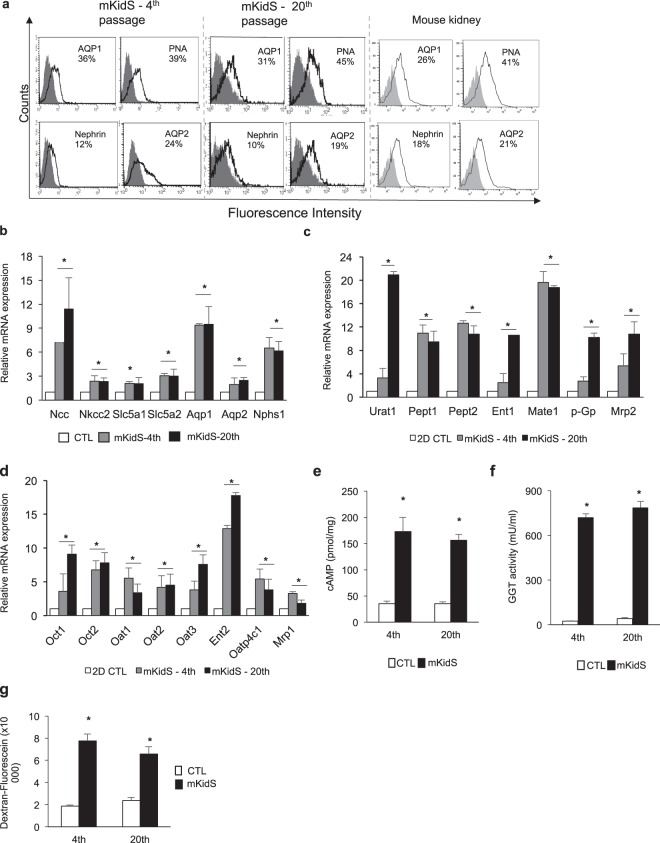
Reproducibility and maturity of kidney spheroids. (**a**) FACS analysis of tubule segment markers in mouse kidney spheroid generated by immortalized cells at the 4^th^ and 20^th^ passages. As a positive control, dissociated mouse whole kidney (at 6 weeks of age) was used. Mouse kidney spheroid at the 4^th^ and 20^th^ passages and dissociated whole mouse kidney were stained with anti-AQP1, PNA, nephrin, or AQP2 (open histograms), or isotype control mAb (grey histograms). (**b**) Quantitative RT-PCR analysis of kidney-specific segment markers in mKidS generated by cells at the 4^th^ and 20^th^ passages compared with 2D controls (2D CTL). n = 8 for 2D CTL and n = 6 for mKidS at the 4^th^ and 20^th^ passages. (**c,d**) Relative mRNA expression of apical (**c**) and basolateral (**d**) transporters in mKidS at the 4^th^ and 20^th^ passages compared with 2D cultured cells. n = 8 for 2D CTL and n = 6 for mKidS at the 4^th^ and 20^th^ passages. (**e**) Responses to parathyroid hormone (PTH) of mKidS at the 4^th^ and 20^th^ passages compared to 3D CTL. (**f**) GGT activity in mKidS at the 4^th^ and 20^th^ passages compared to 3D CTL. (**g**) Alexa Fluor 488-conjugated dextran uptake of mKidS at the 4^th^ and 20^th^ passages compared with 3D CTL. n = 6 for 3D CTL and mKidS at the 4^th^ and 20^th^ passages for E-G. All data are shown as the means ± s.e.m. *p < 0.05 compared with 2D or 3D control by unpaired Student’s *t*-test.

To examine their reproducibility and similarity to adult kidneys, we generated mouse kidney spheroids using early and late passage cells and compared their marker expression to that of dissociated whole mouse kidney (Fig. 4a). Flow cytometry analysis showed that mouse kidney spheroids sustained all segment-specific markers in both early and late passages and these expression patterns were similar to those of dissociated mouse whole kidney (Fig. 4a). The expression levels of segment-specific markers (Fig. 4b) and major uptake and efflux drug transporters (Fig. 4c,d) also increased, compared with those in 2D CTL, and were consistent with those in the spheroids generated by cells at different passages. Increased cAMP levels, GGT activity, and ability of fluorescein-conjugated dextran uptake were also observed in the spheroids generated by late passaged cells (Fig. 4e–g). We also examined the reproducibility of human kidney spheroids, as shown for mouse kidney spheroids (Fig. S9a–f). We generated kidney spheroids using early- (4^th^ passage) and late- (13^th^ passage) passage cells and investigated the mature marker expression levels in human kidney spheroids. Flow cytometry analysis showed that human kidney spheroids sustained all segment-specific markers in both early and late passages, and cAMP levels, GGT activity, and the expression of major uptake and efflux drug transporter were increased in spheroids generated by early and late passaged cells (Fig. S9a–f). In addition, we examined the long-term culture effects of our kidney spheroids to determine whether they retain the expression of renal markers, hormone response, enzyme activity, and protein uptake function (Fig. S10a–d). Quantitative RT-PCR analysis showed that human kidney spheroids sustained all segment-specific markers for 21 days after maturation (Fig. S10a), and cAMP levels after PTH treatment (Fig. S10b), GGT activity (Fig. S10c), and protein uptake (Fig. S10d) ability were also maintained for 21 days after maturation. In conclusion, we properly generated functional kidney spheroids using cell lines at the late passage and they maintained their functionality for a long time. These data suggested that our protocol is useful to produce kidney spheroids reproducibly and effectively.

### Kidney spheroids provided a platform for *in vitro* nephrotoxicity testing

**Figure 5:**
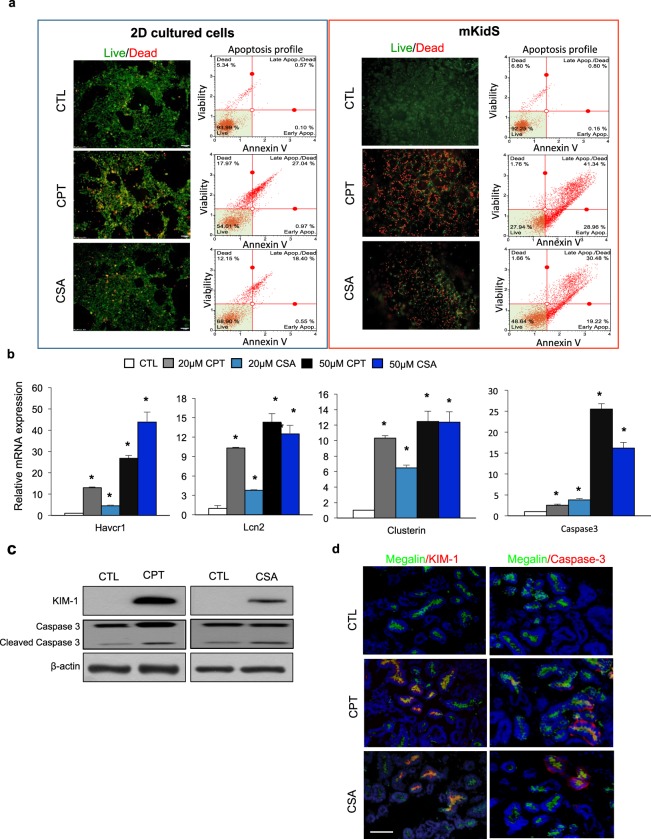
*In vitro* nephrotoxicity assessment of kidney spheroids. (**a**) Live/dead stained images and apoptosis profile as analyzed by MUSE analyzer in 2D cultured cells (left) and mouse kidney spheroids (right) incubated with 20 μM cisplatin (CPT) or 50 μM cyclosporin A (CSA) for 24 h. Cell viability assay was performed by the Muse Count & Viability reagent (Millipore) following the manufacturer’s protocols. The results were obtained with Muse Count & Viability software module and the statistics are shown as the percentage of viable cells and annexin V-positive apoptotic cells. (**b**) Relative mRNA expression of kidney injury markers, such as Havcr1, Lcn2, clusterin, and caspase-3, in the kidney spheroids after treatment with nephrotoxicants. (**c,d**) Western blotting analysis (**c**) and immunostaining images (**d**) of kidney injury markers in kidney spheroids after treatment with 20 μM cisplatin (CPT) or 50 μM cisplatin cyclosporin A (CSA). All data are shown as the means ± s.e.m. *p < 0.05 compared with control by unpaired Student’s *t*-test. Scale bar, 20 μm.

To evaluate nephrotoxicity, kidney spheroids were exposed to various concentrations of known nephrotoxic compounds, such as cisplatin, cyclosporin A, acyclovir, and doxorubicin. Cell death was analyzed by live/dead staining, and apoptosis profile was evaluated using the MUSE analyzer. The 2D cultured cells displayed low level of cell death after exposure to 20 μM cisplatin or cyclosporin A. Apoptosis analysis showed that 28% and 19% of total cells were apoptotic cells (annexin V positive cells) among 20 μM cisplatin- and cyclosporin A-treated cells, respectively (Fig. 5a, left). However, among 3D kidney spheroids, the percentages of dead cells dramatically increased after cisplatin or cyclosporin A treatment (up to 60% of total cells among cisplatin-treated cells, and 50% of total cells among cyclosporin A-treated cells), and apoptosis profiles showed that most of the dead cells were apoptotic cells (annexin V-positive cells), indicating that our spheroids showed increased sensitivity to nephrotoxic compounds (Fig. 5a, right). The expression of kidney injury markers, such as *Havcr1*, *Lcn2*, *clusterin*, and *caspase-3*, significantly increased in a concentration-dependent manner (Fig. 5b). In addition, KIM-1 and cleaved caspase-3 protein expression was detected in the proximal tubule of cisplatin or cyclosporin A-treated spheroids (Fig. 5c,d). This drug response after nephrotoxicant treatment was maintained in spheroids, under an extended culture period (3d to 21d), which showed similar apoptosis patterns after cisplatin treatment compare to matured human kidney spheroids (0d, Fig. S10e). In addition, similar results were observed following treatment with the antiviral drug acyclovir and the anticancer drug doxorubicin (Fig. S11a–d). To examine variations in response to nephrotoxicants, we performed apoptosis profile analysis of single spheroids after drug exposure. Three different spheroids showed similar apoptosis profile after cyclosporin A and cisplatin treatment (Fig. S12).

**Figure 6:**
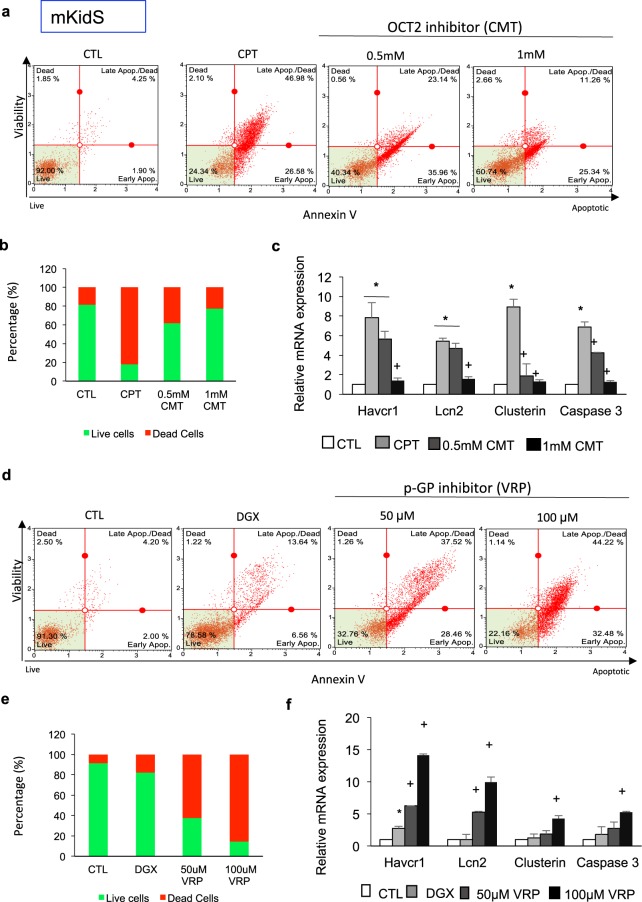
Functional characterization of uptake and efflux transporter activity in mouse kidney spheroids. (**a**) Apoptosis profile by MUSE analyzer in mouse kidney spheroids (mKidS) incubated with 20 μM cisplatin (CPT) with and without preincubation with cimetidine (CMT), an OCT-2 transporter inhibitor. Cell viability assay was performed by the Muse Count & Viability reagent (Millipore) following the manufacturer’s protocols. The results were obtained with Muse Count & Viability software module and the statistics are shown as the percentage of viable cells and annexin V-positive apoptotic cells. (**b**) Quantification of live/dead-stained mKidS incubated with 20 μM CPT with and without preincubation with CMT, an OCT-2 transporter inhibitor. (**c**) Quantitative RT-PCR analysis of kidney injury markers Havcr1, Lcn2, clusterin, and caspase-3 in mKidS compared with untreated controls. n = 12 for CTL, CPT, and CMT, respectively. (**d**) Apoptosis profile as analyzed by MUSE analyzer in mouse kidney spheroids (mKidS) incubated with 10 μM digoxin (DGX) with and without preincubation with verapamil (VRP), a p-Gp efflux transporter inhibitor. (**e**) Quantification of live/dead stained mKidS incubated with 10 μM DGX with and without preincubation with VRP. (**f**) Quantitative RT-PCR analysis of kidney injury markers Havcr1, Lcn2, clusterin, and caspase-3 in mKidS compared with untreated controls. n = 12 for CTL, DGX, and VRP, respectively. All data are shown as the means ± s.e.m. *p < 0.05 compared to control by unpaired Student’s *t* test.

**Figure 7:**
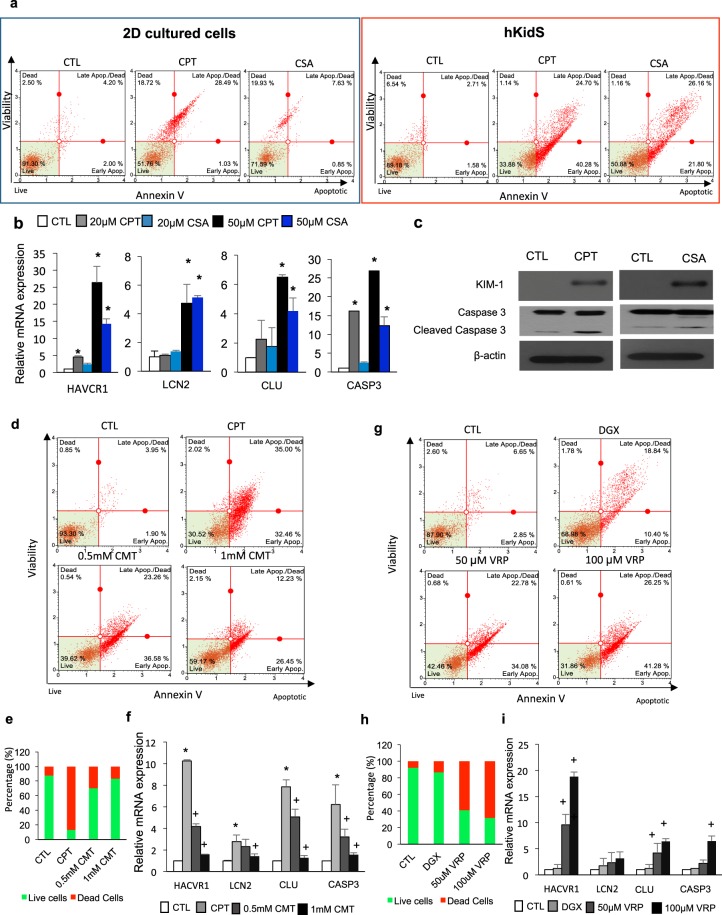
*In vitro* nephrotoxicity assessment and functional characterization of uptake and efflux transporter activity in human kidney spheroids. (**a**) Apoptosis profile as analyzed by MUSE analyzer in 2D cultured cells (left) and human kidney spheroids (hKidS, right) incubated with 20 μM cisplatin (CPT) or 50 μM cyclosporin A (CSA) for 24 h. Cell viability assay was performed by the Muse Count & Viability reagent (Millipore) following the manufacturer’s protocols. The results were obtained with Muse Count & Viability software module and the statistics are shown as the percentage of viable cells and annexin V-positive apoptotic cells. (**b**) Relative mRNA expression of kidney injury markers, such as HAVCR1, LCN2, CLU and CASP3, in the kidney spheroids after treatment with nephrotoxicants. (**c**) Western blotting analysis of kidney injury markers in human kidney spheroids after treatment with 20 μM cisplatin (CPT) or 50 μM cisplatin cyclosporin A (CSA). (**d**) Apoptosis profile as analyzed by MUSE analyzer in human kidney spheroids (hKidS) incubated with 20 μM cisplatin (CPT) with and without preincubation with cimetidine (CMT), an OCT-2 transporter inhibitor. (**e**) Quantification of live/dead stained hKidS incubated with 20 μM CPT with and without preincubation with CMT, an OCT-2 transporter inhibitor. (**f**) Quantitative RT-PCR analysis of kidney injury markers HAVCR1, LCN2, CLU, and CASP3 in hKidS compared with untreated controls. n = 8 for CTL, CPT, and CMT, respectively. (**g**) Apoptosis profile by MUSE analyzer in hKidS incubated with 10 μM digoxin (DGX) with and without preincubation with verapamil (VRP), a p-gp efflux transporter inhibitor. (**h**) Quantification of live/dead stained hKidS incubated with 10 μM DGX with and without preincubation with VRP. (**i**) Quantitative RT-PCR analysis of kidney injury markers HAVCR1, LCN2, CLU, and CASP3 in hKidS compared with untreated controls. n = 8 for CTL, DGX, and VRP, respectively. All data are shown as the means ± s.e.m. *p < 0.05 compared to control by unpaired Student’s *t* test.

To further examine the mechanisms of drug-induced nephrotoxicity and the functionality of transporters, we treated spheroids with cimetidine, an inhibitor of OCT2. OCT2 is a poly specific cation transporter responsible for cisplatin uptake. Apoptosis profile and quantification of live/dead staining results indicated that OCT-2-mediated cisplatin uptake was inhibited by cimetidine in a concentration-dependent manner (Fig. 6a,b). The expression of kidney injury markers dramatically decreased in kidney spheroids treated with cimetidine and cisplatin, compared with that in spheroids treated with cisplatin alone (Fig. 6c). We also examined the effects of verapamil in inhibiting p-Gp, a digoxin efflux transporter. Apoptosis profile and quantification of live/dead staining showed that p-Gp-mediated digoxin efflux was inhibited in a concentration-dependent manner by verapamil. The percentage of apoptotic cells dramatically increased by inhibiting efflux transporter function (Fig. 6d,e). *Havcr1, Lcn2, clusterin*, and *caspase-3* expression levels dramatically increased in kidney spheroids treated with verapamil and digoxin compared with that in spheroids treated with digoxin alone (Fig. 6f). Next, we also examined the drug-induced nephrotoxicity of human kidney spheroids by the same protocols to test mouse kidney spheroids. Human kidney spheroids also showed sensitive apoptosis profile in response to nephrotoxicants (Fig. 7a–c), and these responses were mediated by specific transporter activity, similar to that of mouse kidney spheroids (Fig. 7d–i). These results showed that the kidney spheroids recapitulated renal proximal tubule cells functionally, including GGT activity, response to PTH, and protein endocytosis. Moreover, these cells maintained the functional activity of both uptake and efflux transporters, suggesting their potential use in studying drug transporter interactions and evaluating renal toxicants.

## Discussion

Over the last three years, several groups have published direct protocols for differentiation of human pluripotent stem cells to kidney organoids containing multiple kidney lineages^17,18^ for nephrotoxicity testing. Although, structurally, these systems are able to generate tubule-like systems, these organoids are more similar to first-trimester human kidneys rather than mature kidneys, as indicated by marker gene expression analysis^17,18^. Indeed, a recent study indicated that the cellular differentiation of these cells could be highly diverse and large numbers of cells remain immature, as analyzed by single cell RNA sequencing^19^. Other studies established *in vitro* differentiated single cell types, such as iREC (*in vitro* differentiated renal epithelial cells)^29^ and iPSC-derived podocytes^30^. Recently, engineered kidney tubules in polydimethylsilozan scaffold using iPSCs or cells from polycystic kidney disease (PKD) patient showed 3D tubules with renal epithelial properties^31^. The characterization and detailed comparisons of these cells and systems are still on-going. In addition, some studies showed common renal cell lines on a chip for nephrotoxicity assessments *in vitro*^32,33^, although validation against established nephrotoxicity assays is required, along with demonstration of whether 3D renal models exceed the sensitivity and specificity of existing 2D *in vitro* and animal models. In this study, we established new human and mouse kidney cell lines that showed renal progenitor cell-like characteristics, with stable renal gene expression characteristics after repeated culturing. Importantly, these cell lines exhibited the ability to reproducibly differentiate into kidney spheroids using a simple differentiation protocol within 5‒7 days, and the kidney spheroids recapitulated the kidneys structurally and functionally for direct and accurate nephrotoxicity screening.

Previous results^34–38^ showed that renal progenitors express high levels of CD133 and Sox9, and these marker-positive cells *in vivo* contribute to regeneration of the renal epithelium after injury. Hoxb7 is a marker of ureteric bud epithelium in the developing kidney; the ureteric bud epithelium undergoes a series of branching to form the collecting duct system of the kidney^39,40^. In this study, we demonstrated multiple progenitor cells such as Sox9-, Cd133-, and Hoxb7- positive cells in the generated cell lines that were stable during an extended culture period and could be used to generate mature tubular cells for kidney spheroids. BMP7 has been shown to induce cells to take on renal epithelial-like characteristics and improve kidney function recovery, but TGFb1 promotes EMT transition^20,21^. Here, we showed the role of BMP7 secreted by our renal tubule cells in maintaining epithelial-like cells and preventing cells from entering the EMT process *in vitro*. Moreover, in our previous study, cancer cell spheroids generated by SFU protocol showed enhanced aggressiveness through increased cancer stem cell markers and cancer-related genes, mimicking human live cancers^22^. Fluid shear stress also induces reorganization of renal proximal tubule cells and enhances their tight junction^25^, as well as promotes cellular differentiation in various cells^26–28^. In this study, we showed that fluid shear stress generated by rotatory culture using the SFU protocol induced ER stress, which could precede the expression of renal progenitor markers, such as WT1, and mature renal cell markers, such as AQP1, in our spheroid model. XBP1, one of the principal components induced by ER stress, establishes and maintains the subcellular machinery for synthesizing large quantities of proteins during normal development of professional secretory cells^41^. Moreover, activation of XBP-1 protects against polycystic diseases in the setting of impaired biogenesis of polycystin-1^42^. A previous study^43^ and our current study predicted the XBP-1 binding motif in the WT1 promoter, and showed that WT1 can bind to the AQP1 promoter. WT1 is a key regulator of the balance between the mesenchymal and epithelial states in nephron formation and kidney development^44^.

The utility of *in vitro* models is highly dependent on their ability to recapitulate *in vivo* physiology. In the body, proximal epithelial cells reabsorb filtered glucose, amino acids, phosphate, and urea from the glomerular filtrate through kidney-specific transporters^45^. They reabsorb low-molecular-weight proteins *via* endocytosis and show increased cAMP in response to parathyroid hormone^46^. The kidney spheroids in our study showed albumin and dextran uptake as well as GGT activity and increased cAMP in the cytosol after PTH treatment. One of the most critical requirements for *in vitro* models of kidney function is drug toxicity testing. Cisplatin is a well-known chemotherapeutic drug that induces nephrotoxicity in various *in vitro* models, including human embryonic kidney 293, immortalized rat kidney proximal tubule, Madin-Darby canine kidney, and HK-2 cells, as well as isolated human proximal tubules^47–49^. However, species-specific differences in drug transporter expression and loss of mature features in human cell lines and even primary cells, limit the predictive value of current *in vitro* models^50^. Furthermore, certain transporters involved in nephrotoxicity are differently expressed and localized in different species, and this is a major reason for the different effects of drugs observed in *in vitro* and *in vivo* models^51,52^. In this study, kidney spheroids generated from both mouse and human renal primary cells showed increased expression of all apical and basolateral transporters, and they exhibited dose-dependent, higher fidelity toxicity responses to diverse nephrotoxins, including cisplatin. We also showed that these nephrotoxic events were drug-induced apoptotic events, and these responses were mediated *via* specific transporter activity using an uptake and efflux inhibitor, such as cimetidine, a blocker of the uptake organic cation transporter OCT2, and verapamil, an inhibitor of the efflux transporter p-glycoprotein that mediates the efflux of certain cancer chemotherapies, which can lead to multidrug resistance^53^.

In summary, we established mixed kidney cell lines that were enriched for heterogeneous renal progenitor marker expression and stable characteristics that were maintained following cell proliferation. We established a simple protocol using SFU in the ECM that allowed further differentiation of these cells into functional kidney spheroids that recapitulate the kidney structurally and functionally. These cells differentiated into polarized tubules and showed renal-specific enzyme activity, protein uptake, hormone response, and drug transporter expression. The spheroids exhibited both uptake and efflux transport functions. These data indicated that this kidney spheroid might provide a useful *in vitro* model for studying renal physiology, kidney diseases, and nephrotoxicity.

## Methods

### Antibodies and reagents

LTL (IF, Vector Labs #FL-1321/ ICC, Vector Labs, #B-1325), DBA (IF, Vector Labs, #FL-1031), PNA (IF, Vector Lab #FL-1071/ ICC, Vector Labs, #B-1075), aquaporin 1 (IF, Santacruz, #sc32737), zo-1 (IF, invitrogen, #40–2200), PTHR (IF, Novus, #NBP1-51640), e-cadherin (WB, and IF, BD biosciences, #610181), vimentin (WB, santacruz, #sc6260), snail (WB, santacruz, #sc28199), XBP-1 (WB, Cell signaling technology, #12782 S), WT1 (WB, santacruz, #sc192), ATF4 (WB, Cell signaling technology, 11815), ATF6 (WB, Cell signaling technology, 65880 S), megalin (IF, Abcam, #ab76969), aquaporin 2 (ICC/IF, Bioss, #bs-4611R), Nephrin (ICC/IF, R&D systems, #AF3159), fluorescein-dextran (Invitrogen, #D3301), fluorescein-albumin (Life technology, #A23015), caspase 3 (Cell signaling technology, #9662 S), KIM-1 (Novus, #NBP1-76701), NGAL (Novus, #NBP1-05162), DMEM/F12 (Gibco), FBS (Gibco), EGF (Peprotech), bFGF (Peprotech), Insulin-Transferrin-Selenium (ITS, Gibco), 1α,25-Dihydroxyvitamin D3 (Sigma), all-trans retinoic acid (Sigma) RNAeasy kit (Qiagen, Valencia, CA), collagenase I (Worthington Biochemical Product), 100 μm cell strainer (Fisherbrand).

### Primary culture of renal tubule cells

Kidneys were collected from mice add strain (male, C57BL/6, 3–5 weeks old) and humans from patients who underwent surgery for nephrectomy at Chungnam National University Human Resources Bank (Daejeon, South Korea) under accordance with relevant guidelines and regulations. Informed consent was obtained from patients for the use of specimens for research purpose only. We selected the healthy and normal kidneys (above 65 of GFR value) for cell isolation. Cells were isolated by 2 mg/mL collagenase I digestion for 30 min at 37 °C with gentle stirring. Cells were then filtered through a 100 μm mesh to isolate single cells. Cell suspensions were cultured in Dulbecco’s modified Eagle’s medium with nutrient mixture F-12 (DMEM/F12) supplemented with 10% fetal bovine serum, 20 ng/mL epidermal growth factor, and 1% penicillin/streptomycin at 5% CO_2_ and 37 °C. After 2–6 hours, cells were incubated with hTERT and SV40 lentiviruses and 4 μg/mL polybrene. Cells were detached using 0.1% trypsin-ethylenediaminetetraacetic acid at 80% confluence and passaged. The cumulative population doubling with each subculture was calculated by the formula 2 ×  = NH/NI, where NI was the number of cells seeded, NH was the number of cells harvested at confluence ( > 80%), and X was the population doubling. The calculated population doubling was then added to previous population doublings to yield the cumulative population doubling level. To determine the half maximal inhibitory concentration, mouse and human immortalized primary cells (2 × 10^3^ cells) were seeded into 96-well plates and incubated in DMEM/F12 containing serially diluted drugs for 24 h. Cell viability was measured using the CytoX cell viability assay kit (LPS Solution, Daejeon, Korea) according to the manufacturer’s instructions.

### Ethics

All experimental protocols were approved by the Institutional Ethics Committee/IRB of the Korea research institute of bioscience and biotechnology (KRIBB).

### Cell sorting using immunomagnetic beads

We generated single marker depletion population from immortalized cells using anti-AQP1, AQP2, podocin, or THP antibody and MACS columns (Miltenyi Biotec). The assay was performed according to the manufacturer’s instructions.

### Quantitative real-time polymerase chain reaction (qRT-PCR)

RNA was isolated using the RNAeasy Mini kit, 1 μg was reverse transcribed using the cDNA archival kit (Life Technologies, Gaithersburg, MD), and qPCR was performed according to the manufacturers’ instructions (Applied Biosystems, Waltham, MA, USA, and Agilent Technologies) using SYBRGreen Master Mix. The data were normalized and analyzed using the ^ΔΔ^CT method. Primers used are listed in Supplementary Table I.

### Colony forming unit assay

Primary and immortalized cells (1 × 10^3^) were plated onto coverslips in 6-well culture dishes. On day 7, cells were fixed with methanol and stained with 0.005% crystal violet staining solution for 5 min at room temperature.

### Generation of kidney spheroids using mouse and human renal primary cell lines

Immortalized renal cells were suspended in a 2:1 mixture of ECM gel (Sigma) and matrigel and 50 μL droplets at 1~2 × 10^4^ cells/μL were deposited on the inverted lid of a culture dish. Then the lid was placed onto a phosphate-buffered saline (PBS)-filled dish, and after gelation (3–6 h later), cell in the matrix were transferred to the SFU filled with DMEM/F12 supplemented with 10% FBS, 1X Insulin-Transferrin-Selenium (ITS), 20 ng/mL epidermal growth factor (EGF), 100 nM dexamethasone, 20 uM 1α,25-Dihydroxyvitamin D3 and 5 μM all-trans retinoic acid (ATRA) for 6 h. Cells were then cultured for 2–5 additional days without ATRA. For nephrotoxicity tests, kidney spheroids were treated with various concentrations of drugs in serum-free DMEM/F12 for 24 h, and for transporter activity assays, spheroids were pretreated overnight with cimetidine or verapamil before drug treatment.

### Western blot analysis

Cells were lysed in radioimmunoprecipitation assay buffer (50 mM Tris-HCl, pH 8.0, 150 mM NaCl, 5 mM ethylenediaminetetraacetic acid, and 0.1% sodium dodecyl sulfate) and the cell debris was cleared by centrifugation at 15 000 × *g* for 10 min. The lysates were boiled in sodium dodecyl sulfate sample buffer for 5 min. The proteins were resolved by sodium dodecyl sulfate-polyacrylamide gel elctrophoresis and transferred to polyvinylidene difluoride membranes (Millipore, Billerica, MA, USA). The membranes were blocked in 5% skim milk in PBS containing 0.5% Tween-20 at room temperature for 1 h. The membranes were incubated with the appropriate primary antibodies for 1 h at room temperature or overnight at 4 °C and then with secondary antibodies for 1 h at room temperature. The proteins were detected with a chemiluminescence kit (Intron Biotech, Seoul, Korea, and Millipore).

### Live/dead cell staining

Cell viability after drug treatment was examined using a two-color live/dead cell assay kit (Invitrogen, Waltham, MA, USA). Calcein-AM (2 μM) and ethdium homodimer-1 (4 μM) were added to cells and incubated for 15 min at 37 °C with 10% CO_2_. After washing with PBS, the cells were visualized under a fluorescence microscope (Panasonic, Kadoma, Osaka, Japan). Calcein and ethdium homodimer-1 were excited using green (485 ± 10 nm) and red (530 ± 12.5 nm) fluorescence filters, respectively.

### Immunofluorescence and immunocytochemical analysis

Spheroids embedded in 2% agar were fixed with 10% formalin, embedded in paraffin, and cut to 5-μm thickness. The sections were incubated in 2% bovine serum albumin with 0.2% fish skin gelatin at room temperature for 1 h to block nonspecific binding, and the sections were incubated with primary antibodies overnight at 4 °C, and then secondary antibodies at room temperature for 1 h. Nuclei were counterstained with 4’,6-diamidino-2-phenylindole.

### Flow cytometry

Renal primary cells and kidney spheroids were harvested by trypsinization, washed, and resuspended in PBS supplemental with 2% FBS. Fluorescein conjugate primary antibody incubation was applied for 30 mins at 4 °C and rinsed and resuspended in PBS. All data acquisition was performed on a FACS Aria or FACSCalibur flow cytometer (BD Biosciences) with CellQuest Pro software.

For MUSE analysis, cells treated drugs suspended 100 μl of serum free DMEM/F12 were transferred in suspension to a new tube and incubated with 100 μl of Muse Annexin V & Dead Cell reagent (Millipore) for 20 minutes at room temperature. The apoptosis was determined by Muse Cell Analyzer (Millipore) and the statistics were shown the percentages of the cells represented by alive, apoptosis and dead population.

### RNA-seq library sequencing

Total RNA was extracted from tissue samples using the RNeasy Mini Kit (Qiagen, Hilden, Germany) according to the manufacturer’s instructions. The extracted RNA samples were analyzed using an Agilent 2100 Bioanalyzer system (Agilent Biotechnologies, Palo Alto, USA) with the RNA 6000 Nano Labchip Kit. Only samples of high-quality RNA (RNA Integrity Number ≥7.5) were used in the following mRNA sample preparation for sequencing. Approximately 0.5 to 4 μg of total RNA was used for construction of each RNA-seq library. For library construction and purification were done according to the TruSeq RNA Sample Preparation Kit V2. The libraries were clustered using HiSeq Rapid Cluster Kit V2 with Flow cell V2 and HiSeq rapid SBS V2. Samples sequencing was performed on Illumina HiSeq2500 machines (Illumina, San Diego, CA, USA) using the standard Illumina RNA-seq protocol with a read length of 2 × 100 bases. The samples were sequenced multiplexed 10 per lane, producing an average of 19 million mappable read pairs per sample.

### GGT activity

GGT activity was measured by following the release of para-nitroanilide from gamma glutamyl-p-nitroanilide using a GGT activity colorimetric assay kit (BioVision, Milpitas, CA, USA). Cell and kidney spheroids were homogenized in 200 μL of ice-cold GGT assay buffer, and 10 μL aliquots were combined with 90 μL of GGT substrate solution and added to the assay plate for a 5 h incubation. Absorbance changes at 418 nm were measured every 30 min at 37 °C.

### Response to parathyroid hormone (PTH)

PTH was obtained from Prospec (New Brunswick, NJ, USA). After overnight incubation with 0.1 mM 3-isobutyl-1-methylxanthine, a phosphodiesterase inhibitor, cells were treated with 100 nM PTH for 30 min. Intracellular cAMP was measured using the cAMP direct EIA kit.

### Albumin and Dextran Uptake

Fluorescein-labeled albumin and dextran were incubated with the cells for 6 h at 4 or 37 °C. Cells were washed 8 times with ice cold Ringers Solution (pH 7.3) and lysed with 0.1% Triton X-100 in 1 × MOPS. Fluorescence was measured at 485/520 nm.

### BMP7 and TGFb1 secretion

Cells were starved with serum free culture medium for 24 h and supernatant was collected for BMP7 and TGFb1 secretion assessments using mouse BMP7 and TGFb1 ELISA quantitation kit (Komabiotech, Korea). The assay was performed according to the manufacturer’s instructions.

### Statistical analysis

Data are displayed as the mean ± standard error of the mean and were analyzed with excel and GraphPad Prism version 5.0 (GraphPad Software). Statistical significance was determined using a two-tailed Student’s t-test. *p* < 0.05 was considered significant.

## Supplementary information

Supplemental information

## References

[1] Tiong HY (2014). Drug-induced nephrotoxicity: clinical impact and preclinical *in vitro* models. Mol Pharm.

[2] Naughton CA (2008). Drug-induced nephrotoxicity. Am Fam Physician.

[3] Schultze AE (2013). Current practices in preclinical drug development: gaps in hemostasis testing to assess risk of thromboembolic injury. Toxicol Pathol.

[4] Isoda K (2017). Hepatotoxicity, nephrotoxicity, and drug/chemical interaction toxicity of platinum nanoparticles in mice. Pharmazie.

[5] Huang J, Wu H (2017). Drug-induced nephrotoxicity: pathogenic mechanisms, biomarkers and prevention strategies. Curr Drug Metab.

[6] Choudhury D, Ahmed Z (2006). Drug-associated renal dysfunction and injury. Nat Clin Pract Nephrol.

[7] Bens M, Vandewalle A (2008). Cell models for studying renal physiology. Pflugers Arch.

[8] Lohr JW, Willsky GR, Acara MA (1998). Renal drug metabolism. Pharmacol Rev.

[9] Zucco F, De Angelis I, Testai E, Stammati A (2004). Toxicology investigations with cell culture systems: 20 years after. Toxicol In Vitro.

[10] Gunness P, Aleksa K, Kosuge K, Ito S, Koren G (2010). Comparison of the novel HK-2 human renal proximal tubular cell line with the standard LLC-PK1 cell line in studying drug-induced nephrotoxicity. Can J Physiol Pharmacol.

[11] Ryan MJ (1994). HK-2: an immortalized proximal tubule epithelial cell line from normal adult human kidney. Kidney Int.

[12] Wieser M (2008). hTERT alone immortalizes epithelial cells of renal proximal tubules without changing their functional characteristics. Am J Physiol Renal Physiol.

[13] Li S (2017). Development and Application of Human Renal Proximal Tubule Epithelial Cells for Assessment of Compound Toxicity. Curr Chem Genom Transl Med.

[14] Shaw G, Morse S, Ararat M, Graham FL (2002). Preferential transformation of human neuronal cells by human adenoviruses and the origin of HEK 293 cells. FASEB J.

[15] Jenkinson SE (2012). The limitations of renal epithelial cell line HK-2 as a model of drug transporter expression and function in the proximal tubule. Pflugers Arch.

[16] Aschauer L, Carta G, Vogelsang N, Schlatter E, Jennings P (2015). Expression of xenobiotic transporters in the human renal proximal tubule cell line RPTEC/TERT1. Toxicol In Vitro.

[17] Takasato M (2015). Kidney organoids from human iPS cells contain multiple lineages and model human nephrogenesis. Nature.

[18] Freedman BS (2015). Modeling Kidney Disease with iPS Cells. Biomark Insights.

[19] Wu H, U. K., Donnelly E, Kirita Y, Morris SA, Humphreys BD. Comparative analysis of kidney organoid and adult human kidney single cell and single nucleus transcriptomes. *bioRxiv* (2017).

[20] Tsujimura T, Idei M, Yoshikawa M, Takase O, Hishikawa K (2016). Roles and regulation of bone morphogenetic protein-7 in kidney development and diseases. World journal of stem cells.

[21] Zeisberg M (2003). BMP-7 counteracts TGF-beta1-induced epithelial-to-mesenchymal transition and reverses chronic renal injury. Nat Med.

[22] Jung HR (2017). Cell Spheroids with Enhanced Aggressiveness to Mimic Human Liver Cancer *In Vitro* and *In Vivo*. Sci Rep.

[23] Fong AH (2016). Three-Dimensional Adult Cardiac Extracellular Matrix Promotes Maturation of Human Induced Pluripotent Stem Cell-Derived Cardiomyocytes. Tissue Eng Part A.

[24] Aarskog D, Aksnes L, Markestad T (1983). Effect of parathyroid hormone on cAMP and 1,25-dihydroxyvitamin D formation and renal handling of phosphate in vitamin D-dependent rickets. Pediatrics.

[25] Duan Y (2008). Shear-induced reorganization of renal proximal tubule cell actin cytoskeleton and apical junctional complexes. Proceedings of the National Academy of Sciences of the United States of America.

[26] Datta N (2006). *In vitro* generated extracellular matrix and fluid shear stress synergistically enhance 3D osteoblastic differentiation. Proceedings of the National Academy of Sciences of the United States of America.

[27] Yourek G, McCormick SM, Mao JJ, Reilly GC (2010). Shear stress induces osteogenic differentiation of human mesenchymal stem cells. Regenerative medicine.

[28] Wolfe RP, Ahsan T (2013). Shear stress during early embryonic stem cell differentiation promotes hematopoietic and endothelial phenotypes. Biotechnology and bioengineering.

[29] Lagies S (2018). Metabolic characterization of directly reprogrammed renal tubular epithelial cells (iRECs). Sci Rep.

[30] Musah, S. *et al*. Mature induced-pluripotent-stem-cell-derived human podocytes reconstitute kidney glomerular-capillary-wall function on a chip. *Nat Biomed Eng* **1**, 10.1038/s41551-017-0069 (2017). 10.1038/s41551-017-0069 PMC5639718 29038743

[31] Benedetti V (2018). Engineered Kidney Tubules for Modeling Patient-Specific Diseases and Drug Discovery. EBioMedicine.

[32] DesRochers TM, Suter L, Roth A, Kaplan DL (2013). Bioengineered 3D human kidney tissue, a platform for the determination of nephrotoxicity. PLoS One.

[33] Jang KJ (2013). Human kidney proximal tubule-on-a-chip for drug transport and nephrotoxicity assessment. Integr Biol (Camb).

[34] Kang HM (2016). Sox9-Positive Progenitor Cells Play a Key Role in Renal Tubule Epithelial Regeneration in Mice. Cell Rep.

[35] Kumar S (2015). Sox9 Activation Highlights a Cellular Pathway of Renal Repair in the Acutely Injured Mammalian Kidney. Cell Rep.

[36] Ma Q, Wang Y, Zhang T, Zuo W (2018). Notch-mediated Sox9(+) cell activation contributes to kidney repair after partial nephrectomy. Life Sci.

[37] Lazzeri E (2007). Regenerative potential of embryonic renal multipotent progenitors in acute renal failure. Journal of the American Society of Nephrology: JASN.

[38] Brossa A (2018). Role of CD133 Molecule in Wnt Response and Renal Repair. Stem cells translational medicine.

[39] Takasato M (2014). Directing human embryonic stem cell differentiation towards a renal lineage generates a self-organizing kidney. Nature cell biology.

[40] Nagalakshmi VK, Yu J (2015). The ureteric bud epithelium: morphogenesis and roles in metanephric kidney patterning. Molecular reproduction and development.

[41] Lee AH, Chu GC, Iwakoshi NN, Glimcher LH (2005). XBP-1 is required for biogenesis of cellular secretory machinery of exocrine glands. The EMBO journal.

[42] Fedeles SV (2015). Sec. 63 and Xbp1 regulate IRE1alpha activity and polycystic disease severity. J Clin Invest.

[43] Fraizer GC (1994). Transcriptional regulation of the human Wilms’ tumor gene (WT1). Cell type-specific enhancer and promiscuous promoter. The Journal of biological chemistry.

[44] Kreidberg JA (2010). WT1 and kidney progenitor cells. Organogenesis.

[45] Mizuno N, Niwa T, Yotsumoto Y, Sugiyama Y (2003). Impact of drug transporter studies on drug discovery and development. Pharmacol Rev.

[46] Hruska KA (1977). Degradation of parathyroid hormone and fragment production by the isolated perfused dog kidney. The effect of glomerular filtration rate and perfusate CA++ concentrations. J Clin Invest.

[47] Pabla N, Dong Z (2008). Cisplatin nephrotoxicity: mechanisms and renoprotective strategies. Kidney Int.

[48] Ludwig T, Riethmuller C, Gekle M, Schwerdt G, Oberleithner H (2004). Nephrotoxicity of platinum complexes is related to basolateral organic cation transport. Kidney Int.

[49] Ciarimboli G (2005). Cisplatin nephrotoxicity is critically mediated via the human organic cation transporter 2. Am J Pathol.

[50] Pfaller W, Gstraunthaler G (1998). Nephrotoxicity testing *in vitro*–what we know and what we need to know. Environ Health Perspect.

[51] Tahara H (2005). A species difference in the transport activities of H2 receptor antagonists by rat and human renal organic anion and cation transporters. J Pharmacol Exp Ther.

[52] Uetrecht J (2006). Role of animal models in the study of drug-induced hypersensitivity reactions. AAPS J.

[53] Gottesman MM, Pastan I (1993). Biochemistry of multidrug resistance mediated by the multidrug transporter. Annu Rev Biochem.

