# Supplementary material for: Effective reconstruction of functional organotypic kidney spheroid for in vitro nephrotoxicity studies
Source: Sci Rep. 2019 Nov 26;9:17610. doi: 10.1038/s41598-019-53855-2 (PMC6879515; doi:10.1038/s41598-019-53855-2)
Supplement: Supplementary file 1 — Supplemental information [file 41598_2019_53855_MOESM1_ESM.pdf]

Effective reconstruction of functional organotypic kidney spheroid for *in vitro* nephrotoxicity studies

Hyun Mi Kang[1*], Jung Hwa Lim[1*], Kyung Hee Noh[1], Dongmin Park[1], Hyun-Soo Cho[2], Katalin Susztak[3], Cho-Rok Jung[1,4].

[1]Laboratory of Disease Modeling and Therapeutics, Korea Research Institute of Bioscience and Biotechnology, Daejeon, Republic of Korea.

[2]Stem Cell Research Center, Korea Research Institute of Bioscience and Biotechnology, Daejeon, Republic of Korea.

[3]Division of Nephrology, Department of Medicine, Department of Genetics, Perelman School of Medicine, University of Pennsylvania, Philadelphia, PA, USA

[4]Department of Functional Genomics, Korea University of Science and Technology, Daejeon, Republic of Korea.

* These author contributed equally to this work.

Correspondence and requests for materials should be addressed to Katalin Susztak (ksusztak@pennmedicine.upenn.edu) or Cho-Rok Jung (crjung@kribb.re.kr).

Supplemental information

Supplemental figure 1. Characterization of immortalized cells derived from mouse kidney.

a. Schematic diagram of the isolation and immortalization of cells with high proliferative capacity. **b-c**. hTERT (b) and SV40 (c) expression in immortalized cells at different passage. **d**. Cumulative doubling numbers for three primary cell lines and three immortalized cell lines throughout *ex vivo* expansion. **e.** Relative transcript levels of mesenchymal stem cell markers in mouse kidney (mKidney), and immortalized cells at the fifth and 15th passages. n=3 of each group. All data are shown as the means ± s.e.m. *P<0.05 compared to mKidney by unpaired Student's t test.

Supplemental figure 2. Characterization of primary cells derived from mouse kidney.

a. Morphology of mouse tubular epithelial cells during *in vitro* culture. Cell changed their morphology to fibroblast like cells after passage 4. **b.** Quantitative RT-PCR analysis of EMT markers such as vimentin, fibronectin and e-cadherin in mouse cells at the primary, and 4th passages. n=3 of each group. **c.** Related mRNA levels of kidney segment specific markers in the cell at primary, 1st and 3rd passage. n=3 of each group. All data are shown as the means ± s.e.m. *P<0.05 compared to primary cells by unpaired Student's t test.

Supplemental figure 3. Characterization of single segment depletion cells from immortalized cells.

a. Morphology of single segment depletion cells after magnetic activated cell sorting. Cells were maintained same methods used for culturing immortalized cells. Cell of

AQP1 or THP depletion cells changed their morphology to fibroblast like cells. **b.** Quantitative RT-PCR analysis of AQP1, AQP2, podocin (PODO), and THP to show to lose of single marker in the depletion cells. **c.** FACS analysis of tubule segment markers in AQP1, AQP2, podocin or THP depletion cells. CTL cells (open black histograms) and each marker depletion cells (open red histograms) were stained with anti-AQP1, AQP2, podocin or THP or isotype control mAb (grey histograms).

Supplemental figure 4. Analysis of multiple renal progenitor cells in immortalized cells at early and late passage.

FACS analysis of renal progenitor cells markers such as Cd133 and Sox9 (a) and ureteric bud epithelial cell marker as Hoxb7 (b) in mouse immortalized cells at early (4th passage) and late (16th passage) passage. **a**, Fluorescence data from cells at 4th and 16th passage clarifies which cells expressed renal progenitor cells markers, Cd133 and Sox9. The percentage of immortalized cells express only Cd133, only Sox9, or both was presented in right table. **b**, Fluorescence data from cells at 4th and 16th passage clarifies which cells expressed Cd133 and Hoxb7. The percentage of immortalized cells express only CD133, only Hoxb7, or both was presented in right table.

Supplemental figure 5. Characterization of immortalized cells derived from human kidney.

a. Morphology of human tubular epithelial cells during *in vitro* expansion. **b**. Quantitative RT-PCR analysis of kidney-specific segment markers in immortalized human cells at 1st and at the 6th passage. n=3 each group. **c**. Representative images of immunostaining of human primary cells at 2nd passage and immortalized cells at 6th passage for LTL, PNA, AQP2, and nephrin. **d**. GGT activity of human immortalized

primary cells compared to a human primary tubular epithelial cells (hPTECs). n=6 hPTECs and n=8 human renal primary cells. **e**. Dextran uptake by human immortalized cells at 3nd passage after 48 h incubation with Alexa Fluor 488-conjugated dextran. All data are shown as the means ± s.e.m. *$p<0.05$ compared to hPTECs cells by unpaired Student's *t* test. Scale bar, 20μm.

Supplemental figure 6. Characterization of cell aggregates and kidney spheroids embedded gel matrix derived from immortalized primary cells using spheroid forming unit (SFU) protocol.

a-c. Relative mRNA expression of kidney segment specific markers (a), basolateral (b) and apical (c) transporters in cell aggregates using SFU (combination of hanging-drop and rotation) compared to 2D cultured cells. n=6 of 2D cells and cell aggregates. **d**. Representative images of mouse kidney spheroids embedded ECM gel (Sigma), matrigel or mixture of ECM and matrigel as 2:1 ratio at day 5 after SFU stained with hematoxylin and eosin (H&E). The tubular structures were marked by a red arrow in the image. **e.** Gel mixture only (left) and cell-embedded gel mixture after 5 days of maturation (right). **f.** Related mRNA levels of kidney segment specific markers in the kidney spheroid embedded in the mixture of ECM and matrigel compared to spheroids embedded in matrigel. **g-h.** Quantitative RT-PCR analysis of basolateral (g) and apical (h) transporters in kidney spheroids cultured in the various combination of maturation factors for 5 days. CTL means kidney spheroids without factors, DIVE means kidney spheroids cultured in DMEM/F12 supplemented with 10% FBS, 1X ITS, 20 ng/mL epidermal growth factor (EGF), 100 nM dexamethasone, and 20 uM 1α,25-Dihydroxyvitamin D3, VR means kidney spheroids cultured in DMEM/F12 supplemented with 10% FBS, 20 uM 1α,25-Dihydroxyvitamin D3 and 5 μM all-trans

retinoic acid (ATRA) and DIVER-DIVE means kidney spheroids cultured in DMEM/F12 supplemented with 10% FBS, 1X ITS, 20 ng/mL epidermal growth factor (EGF), 100 nM dexamethasone, 20 uM 1α,25-Dihydroxyvitamin D3 and 5 μM all-trans retinoic acid (ATRA) for 6h then cultured in same medium with our ATRA. n=3 of each group. All data are shown as the means ± s.e.m. *P<0.05 compared to cells embedded matrigel by unpaired Student's *t* test.

Supplemental figure 7. Gene Set Enrichment Analysis (GSEA) generated by RNA-seq analysis from kidney spheroids.

The gene expression data generated by RNA-seq was analyzed using GSEA to extract biological knowledge. Highly, significant enriched gene-sets are shown here. **a.** GSEA analysis highlighting strong enrichment for the glutathione metabolism (left) and heatmap analysis of the expression of transcripts related to glutathione metabolism (right). **b.** GSEA analysis highlighting strong enrichment for the sulfur compound metabolic process (left) and heatmap analysis of the expression of transcripts related to sulfur compound metabolic process (right). **c.** GSEA analysis highlighting strong enrichment for response to toxic substance (left) and heatmap analysis of the expression of transcripts related to response to toxic substance (right).

Supplemental figure 8. Effects of shear stress by SFU culture condition on kidney spheroid maturation.

a-b. Relative mRNA (a) and protein (b) expression of ER stress markers such as XBP-1, AFT4, ATF6, and CHOP at 1, 3, 6, and 24 hours of SFU protocol compared to spheroids cultured in static medium for 24 hours (0h of SFU). n=3 of each time point. **c-d.** Quantitative mRNA expression related to early renal progenitor markers (c) and mature segment marker (d) at 1, 3, 6, 24, 48 and 72 hours of SFU protocol compared

to spheroids cultured in static medium for 24 hours (0h of SFU). n=4 of each time point. **e.** Promoter sequence of WT1 and transcription factor binding sites identified in the box. Binding motif of XBP-1 identified in the red. **f.** Promoter sequence of AQP1 and transcription factor binding sites identified in the box. Binding motif of WT1 identified in the red. **g.** Western blot analysis to show the sequential expression of XBP-1, WT1, and AQP1 at the different time point of SFU protocol. **h.** Summary animation of findings that SFU induced the renal spheroid maturation. All data are shown as the means ± s.e.m. *P<0.05 compared to spheroid at 0h of SFU protocol by unpaired Student's *t* test.

Supplemental figure 9. Reproducibility human kidney spheroids

a. FACS analysis of tubule segment markers in human kidney spheroid (hKidS) generated by immortalized cells at the 4th and 13th passages. Kidney spheroid at the 4th and 13th passages were stained with anti-AQP1, PNA, Nephrin, or AQP2 (open histograms), or isotype control mAb (grey histograms). **b**. Quantitative RT-PCR analysis of kidney-specific segment markers in hKidS generated by cells at the 4th and 13th passage compared with 2D controls (2D CTL). n = 6 for 2D CTL and n = 5 for hKidS at the 4th and 20th passages. **c-d**. Relative mRNA expression of apical (c) and basolateral (d) transporters in hKidS at the 4th and 13th passages compared with 2D cultured cells. n = 6 for 2D CTL and n = 5 for hKidS at the 4th and 13th passages. **e**. Responses to parathyroid hormone (PTH) of hKidS at the 4th and 13th passages compared to 3D CTL. **f**. GGT activity in hKidS at the 4th and 13th passages compared to 3D CTL. n = 5 for 3D CTL and hKidS at the 4th and 13th passages for e-f. All data are shown as the means ± s.e.m. *p < 0.05 compared with 2D or 3D control by unpaired Student's t-test. Scale bar, 20 μm.

Supplemental figure 10. Assessments of stability and extended culture effects of human kidney spheroids.

a. Relative mRNA expression of kidney segment specific markers in human kidney spheroids (hKidS) for 21 days (at day 0, 3, 7, 14, and 21) after maturation compared to 2D cultured cells (CTL). n=3 of 2D cells and hKidS at each time point. **b**. Response to parathyroid hormone (PTH) of long-term cultured hKidS at 0d, 3d, 7d, 14d, and 21d compared with 3D control spheroids (without maturation factors, CTL). n = 4 of 3D CTL and hKidS each time point. **c**. GGT activity in hKidS after 21 days of maturation compared to 3D CTL. n=3 of CTL and hKidS at each time point, respectively. **d**. Dextran-fluorescein uptake of long-term cultured hKidS compare to 3D CTL. N = 3 of 3D CTL and hKidS at each time point, respectively. **e**. Apoptosis profile by MUSE analyzer in hKidS at the different time point of extended culture after maturation incubated with 20 μM cisplatin (CPT). Cell viability assay was performed by the Muse Count & Viability reagent (Millipore) following the manufacturer's protocols. The results were obtained with Muse Count & Viability software module and the statistics were shown as the percentage of viable cells and annexin V-positive apoptotic cells. All data are shown as the means ± s.e.m. *P<0.05 compared to untreated control by unpaired Student's *t* test.

Supplemental figure 11. Assessment of response to nephrotoxicants in kidney spheroids.

a. Live/dead stained images of mouse kidney spheroids incubated with 100μM acyclovir for 24 h. **b**. Relative mRNA expression of kidney injury markers such as Hacvr1, Lcn2, and clusterin after 40uM or 100uM acyclovir treatment compare to

untreated spheroids. n=4 of each group. **c.** Live/dead stained images of mouse kidney spheroids incubated with 7.5 uM for 24 h. **d.** Relative mRNA expression of kidney injury markers such as Hacvr1, Lcn2, and clusterin after 3uM or 7.5uM doxorubicin treatment compared to untreated control. n=4 of each group All data are shown as the means ± s.e.m. *P<0.05 compared to untreated control by unpaired Student's *t* test.

Supplemental figure 12. Assessment of spheroid-to-spheroid variation response to nephrotoxicants in kidney spheroids.

Apoptosis profile by MUSE analyzer in there different single a mouse kidney spheroid incubated with 50 μM cyclosporin A or cisplatin for 24 h. Cell viability assay was performed by the Muse Count & Viability reagent (Millipore) following the manufacturer's protocols. The results were obtained with Muse Count & Viability software module and the statistics were shown the percentage of viable cells and annexin V positive apoptotic cells.

Supplemental Table I. Sequences of oligonucleotide primers used for qPCR.

Supplemental Table II. The list of differentially expressed genes when 2D cells and mouse kidney spheroids (mKidS) were compared.

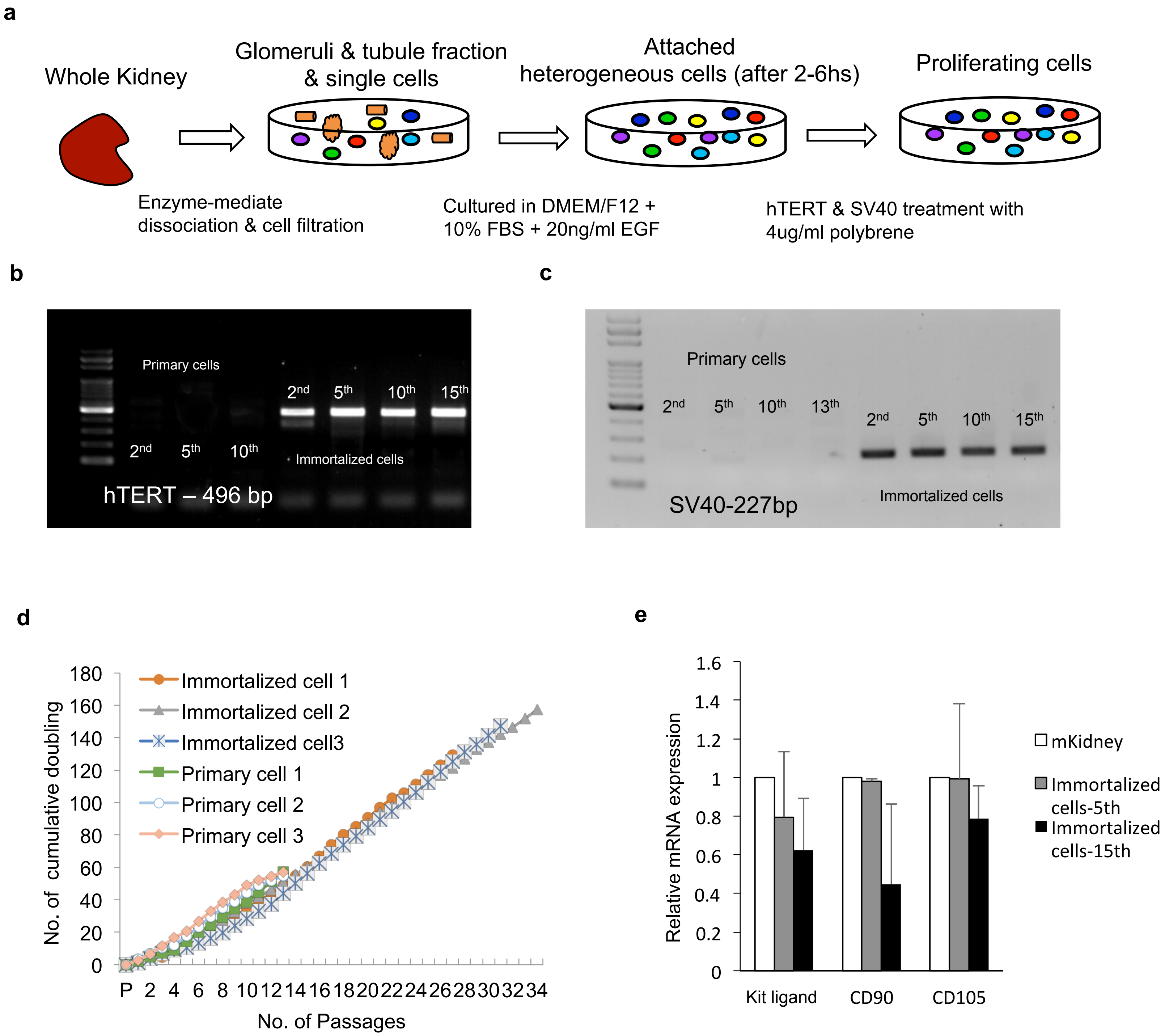
a
Whole Kidney
Glomeruli & tubule fraction & single cells
Attached heterogeneous cells (after 2-6hs)
Proliferating cells
Enzyme-mediate dissociation & cell filtration
Cultured in DMEM/F12 + 10% FBS + 20ng/ml EGF
hTERT & SV40 treatment with 4ug/ml polybrene
b
Primary cells
2nd 5th 10th
2nd 5th 10th 15th
Immortalized cells
hTERT – 496 bp
c
Primary cells
2nd 5th 10th 13th
2nd 5th 10th 15th
SV40-227bp
Immortalized cells
d
Immortalized cell 1
Immortalized cell 2
Immortalized cell3
Primary cell 1
Primary cell 2
Primary cell 3
No. of cumulative doubling
No. of Passages
e
mKidney
Immortalized cells-5th
Immortalized cells-15th
Relative mRNA expression
Kit ligand
CD90
CD105

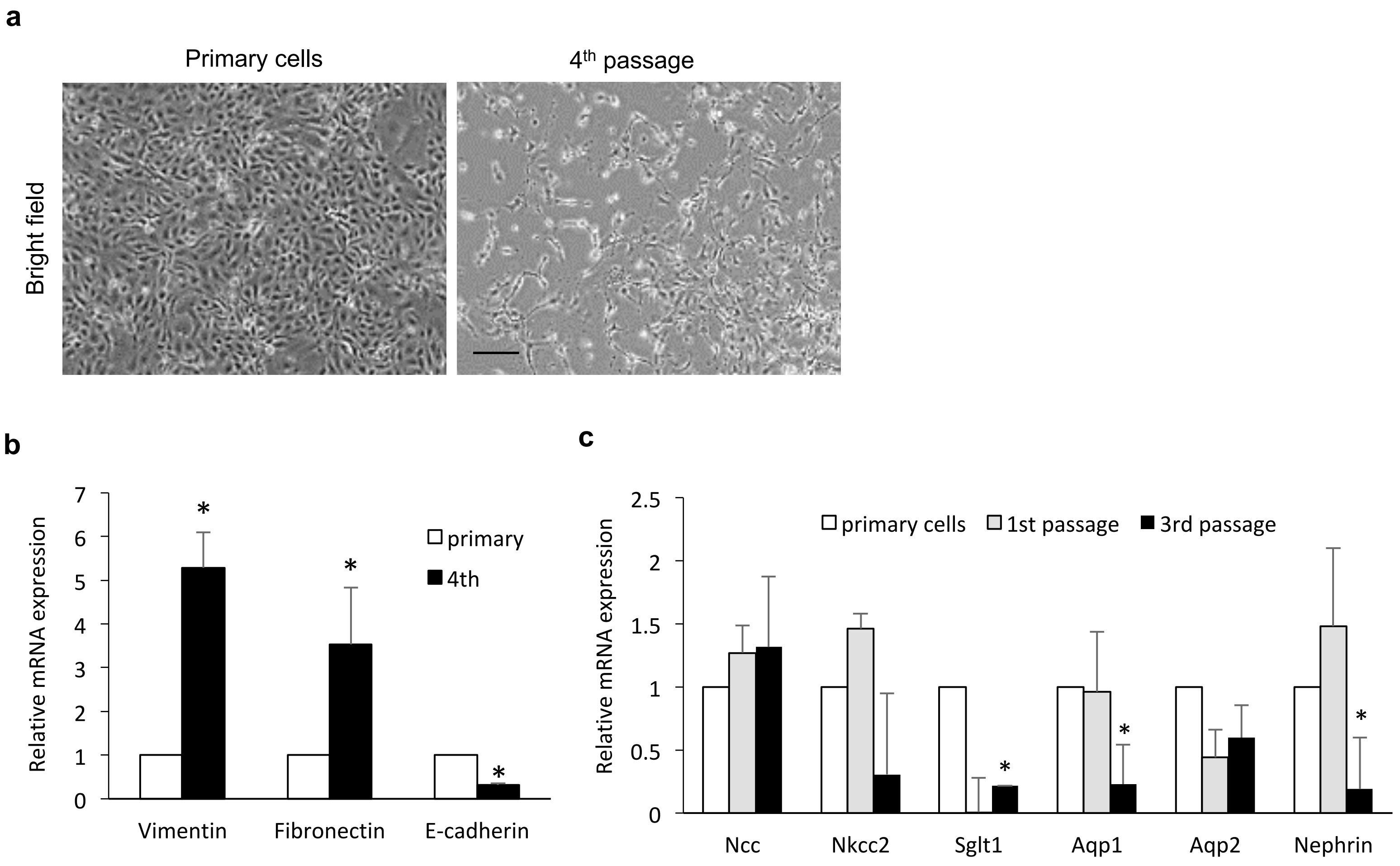

a
Primary cells
4th passage
Bright field
b
Relative mRNA expression
primary
4th
Vimentin
Fibronectin
E-cadherin
c
Relative mRNA expression
primary cells
1st passage
3rd passage
Ncc
Nkcc2
Sglt1
Aqp1
Aqp2
Nephrin

a

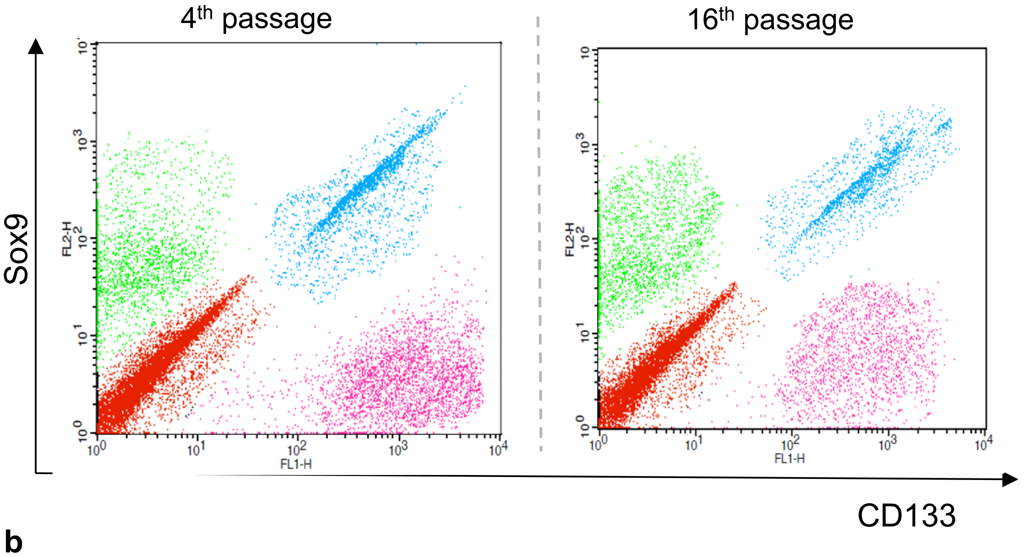

	4th	16th
CD133+ only	21.3%	15.7%
Sox9+ only	18.4%	19.2%
CD133+ Sox9+	16.3%	14.3%

b

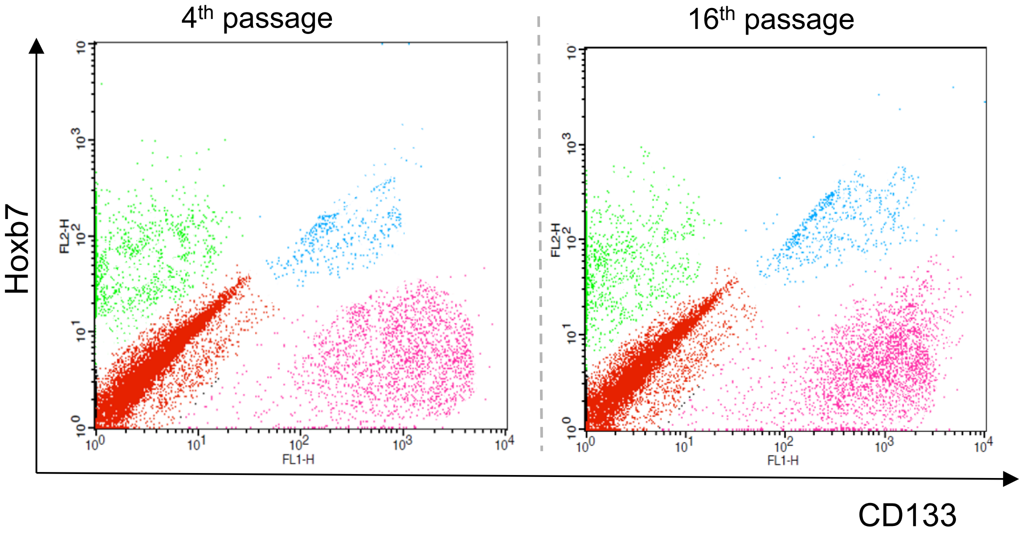

	4th	16th
CD133+ only	19.2%	16.9%
Hoxb7+ only	8.1%	9.4%
CD133+ Hoxb7+	4.5%	5.9%

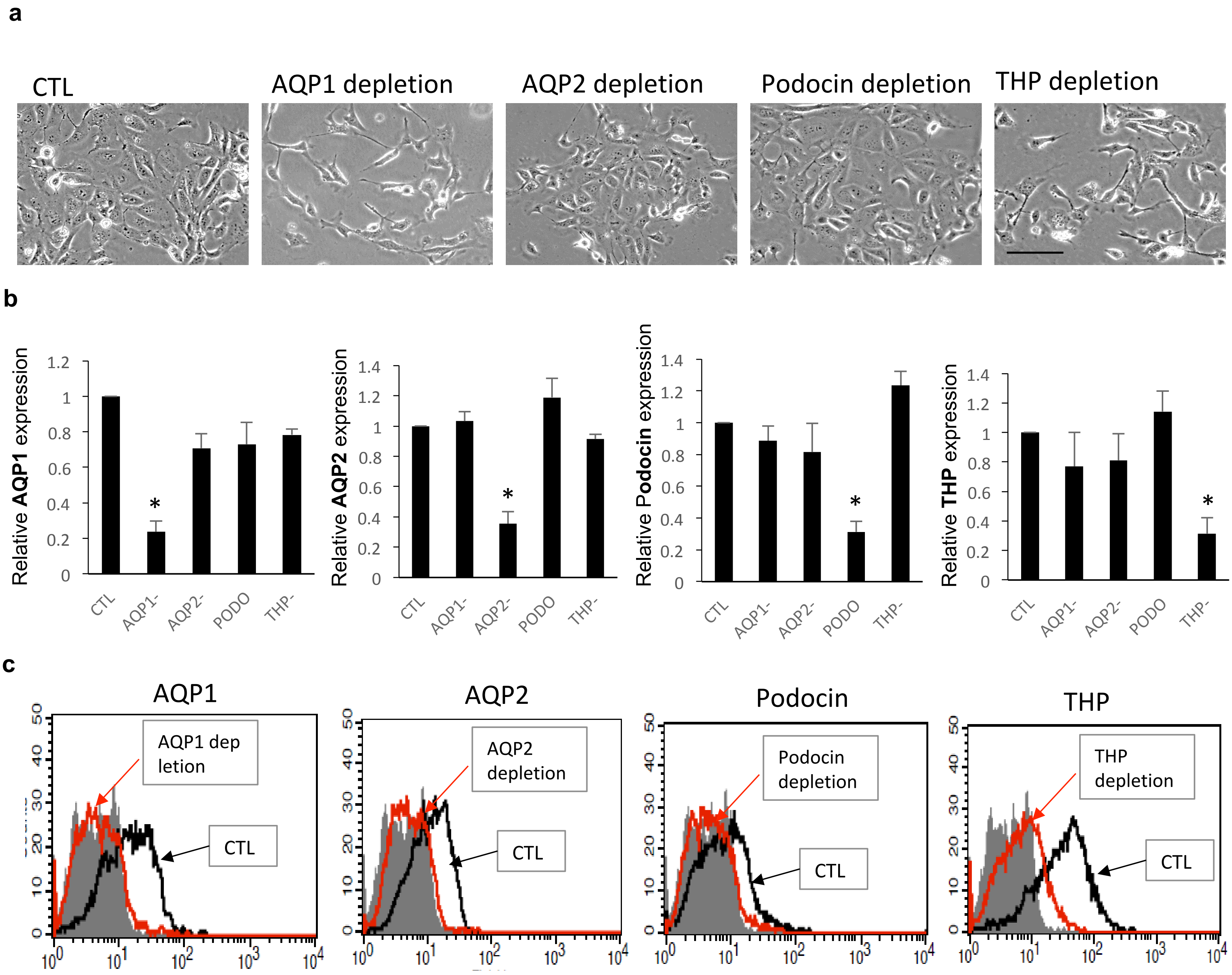
a
CTL
AQP1 depletion
AQP2 depletion
Podocin depletion
THP depletion
b
Relative AQP1 expression
Relative AQP2 expression
Relative Podocin expression
Relative THP expression
CTL
AQP1-
AQP2-
PODO
THP-
c
AQP1
AQP2
Podocin
THP
AQP1 dep letion
AQP2 depletion
Podocin depletion
THP depletion
CTL

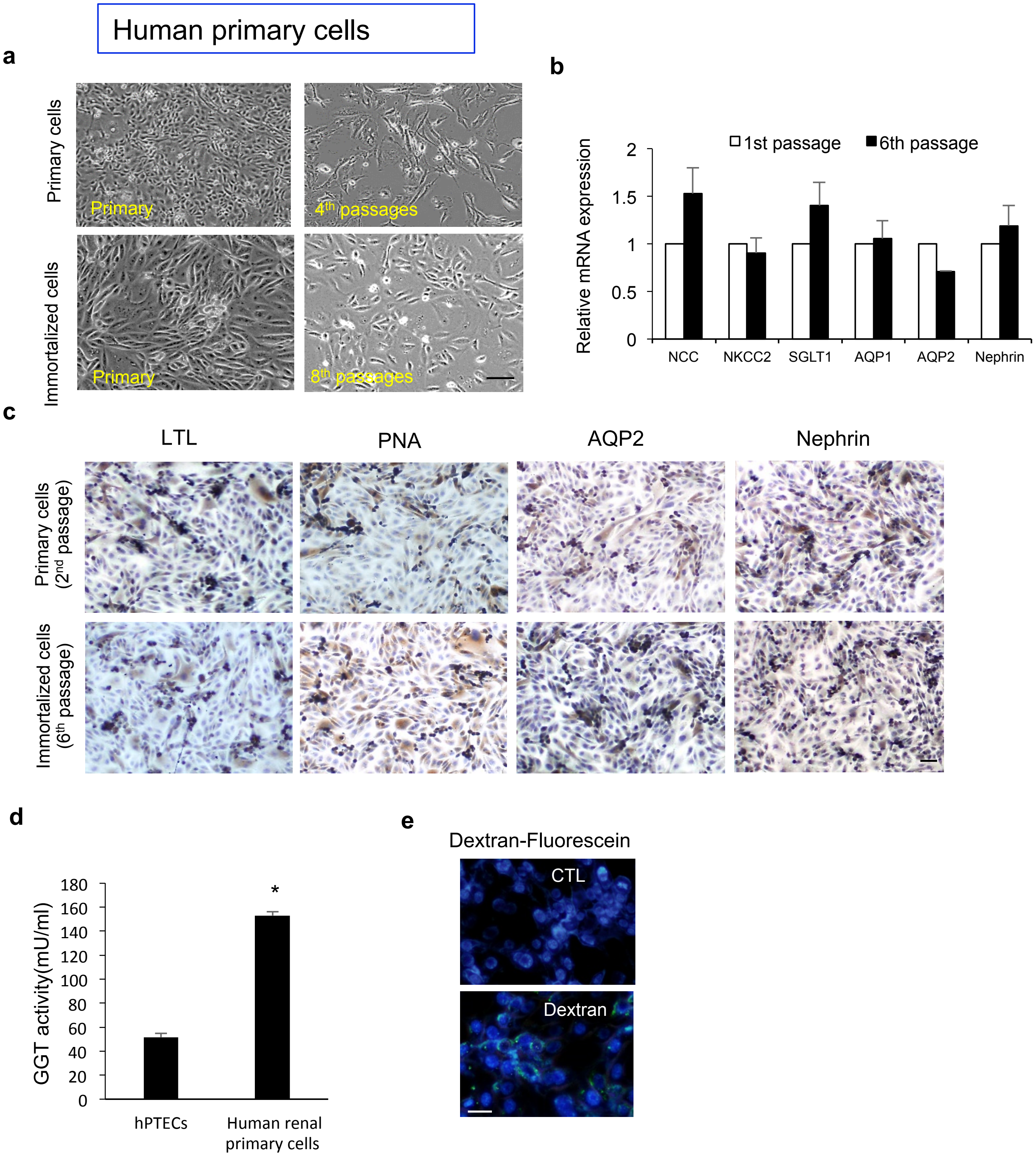
Human primary cells
a
Primary cells
Immortalized cells
Primary
4th passages
Primary
8th passages
b
Relative mRNA expression
1st passage
6th passage
2
1.5
1
0.5
0
NCC
NKCC2
SGLT1
AQP1
AQP2
Nephrin
c
LTL
PNA
AQP2
Nephrin
Primary cells (2nd passage)
Immortalized cells (6th passage)
d
GGT activity(mU/ml)
180
160
140
120
100
80
60
40
20
0
*
hPTECs
Human renal primary cells
e
Dextran-Fluorescein
CTL
Dextran

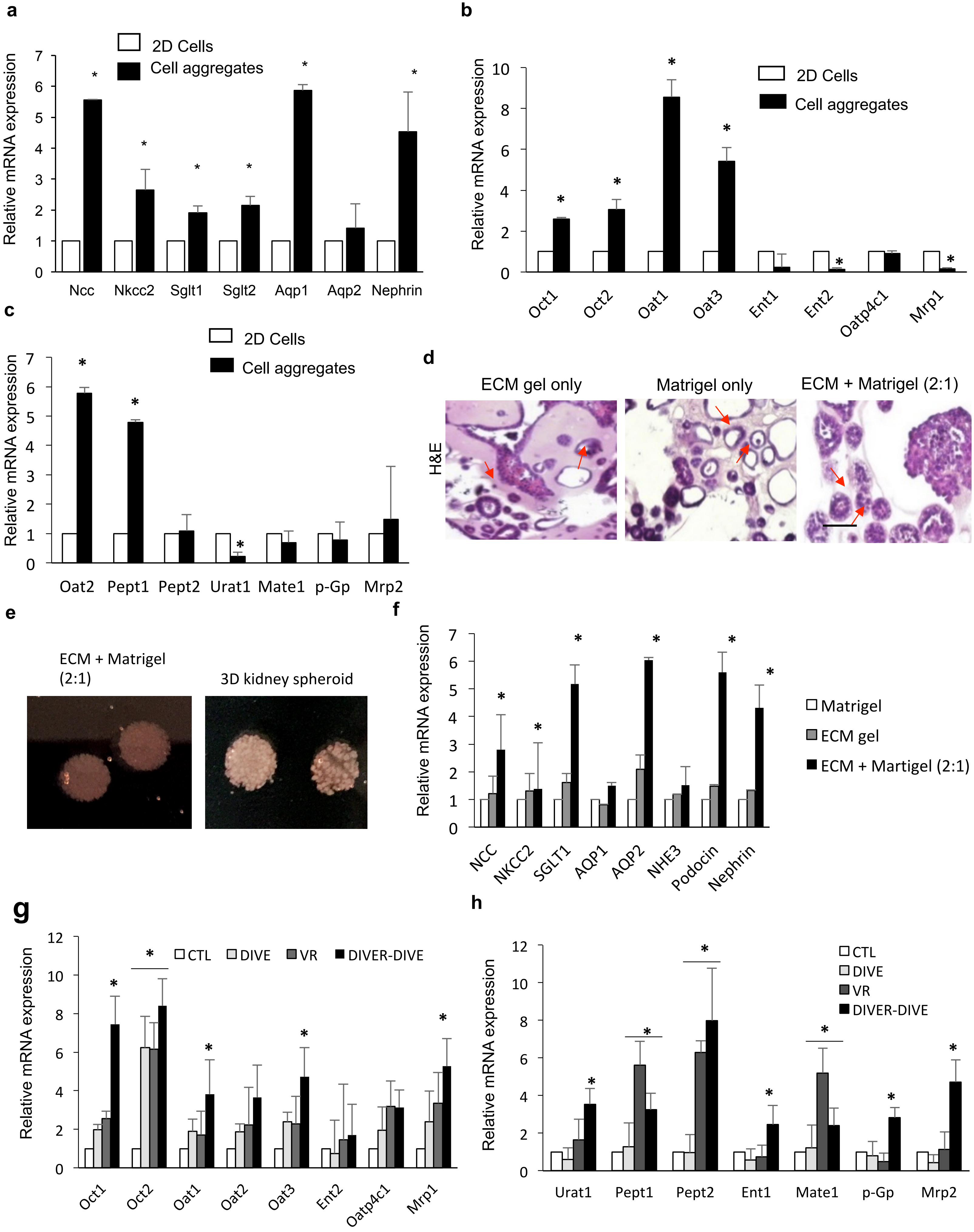
a
2D Cells
Cell aggregates
Relative mRNA expression
Ncc
Nkcc2
Sglt1
Sglt2
Aqp1
Aqp2
Nephrin
b
2D Cells
Cell aggregates
Relative mRNA expression
Oct1
Oct2
Oat1
Oat3
Ent1
Ent2
Oatp4c1
Mrp1
c
2D Cells
Cell aggregates
Relative mRNA expression
Oat2
Pept1
Pept2
Urat1
Mate1
p-Gp
Mrp2
d
ECM gel only
Matrigel only
ECM + Matrigel (2:1)
H&E
e
ECM + Matrigel (2:1)
3D kidney spheroid
f
Relative mRNA expression
Matrigel
ECM gel
ECM + Martigel (2:1)
NCC
NKCC2
SGLT1
AQP1
AQP2
NHE3
Podocin
Nephrin
g
Relative mRNA expression
CTL
DIVE
VR
DIVER-DIVE
Oct1
Oct2
Oat1
Oat2
Oat3
Ent2
Oatp4c1
Mrp1
h
Relative mRNA expression
CTL
DIVE
VR
DIVER-DIVE
Urat1
Pept1
Pept2
Ent1
Mate1
p-Gp
Mrp2

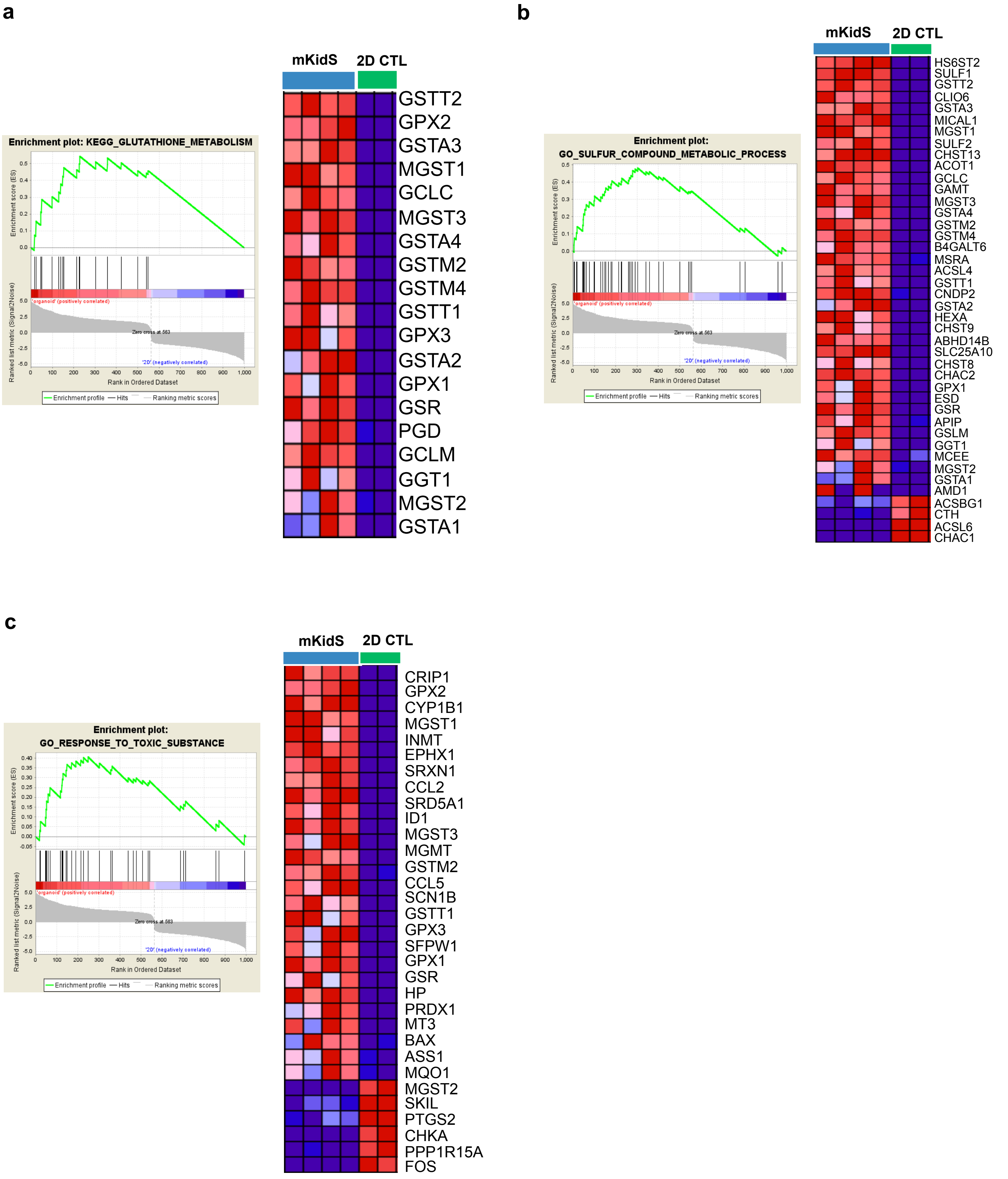

a
Enrichment plot: KEGG_GLUTATHIONE_METABOLISM
Enrichment score (ES)
Ranked list metric (Signal2Noise)
'organoid' (positively correlated)
Zero cross at 563
'2D' (negatively correlated)
Rank in Ordered Dataset
Enrichment profile
Hits
Ranking metric scores
mKidS
2D CTL
GSTT2
GPX2
GSTA3
MGST1
GCLC
MGST3
GSTA4
GSTM2
GSTM4
GSTT1
GPX3
GSTA2
GPX1
GSR
PGD
GCLM
GGT1
MGST2
GSTA1
b
Enrichment plot:
GO_SULFUR_COMPOUND_METABOLIC_PROCESS
Enrichment score (ES)
Ranked list metric (Signal2Noise)
'organoid' (positively correlated)
Zero cross at 563
'2D' (negatively correlated)
Rank in Ordered Dataset
Enrichment profile
Hits
Ranking metric scores
mKidS
2D CTL
HS6ST2
SULF1
GSTT2
CLIO6
GSTA3
MICAL1
MGST1
SULF2
CHST13
ACOT1
GCLC
GAMT
MGST3
GSTA4
GSTM2
GSTM4
B4GALT6
MSRA
ACSL4
GSTT1
CNDP2
GSTA2
HEXA
CHST9
ABHD14B
SLC25A10
CHST8
CHAC2
GPX1
ESD
GSR
APIP
GSLM
GGT1
MCEE
MGST2
GSTA1
AMD1
ACSBG1
CTH
ACSL6
CHAC1
c
Enrichment plot:
GO_RESPONSE_TO_TOXIC_SUBSTANCE
Enrichment score (ES)
Ranked list metric (Signal2Noise)
'organoid' (positively correlated)
Zero cross at 563
'2D' (negatively correlated)
Rank in Ordered Dataset
Enrichment profile
Hits
Ranking metric scores
mKidS
2D CTL
CRIP1
GPX2
CYP1B1
MGST1
INMT
EPHX1
SRXN1
CCL2
SRD5A1
ID1
MGST3
MGMT
GSTM2
CCL5
SCN1B
GSTT1
GPX3
SFPW1
GPX1
GSR
HP
PRDX1
MT3
BAX
ASS1
MQO1
MGST2
SKIL
PTGS2
CHKA
PPP1R15A
FOS

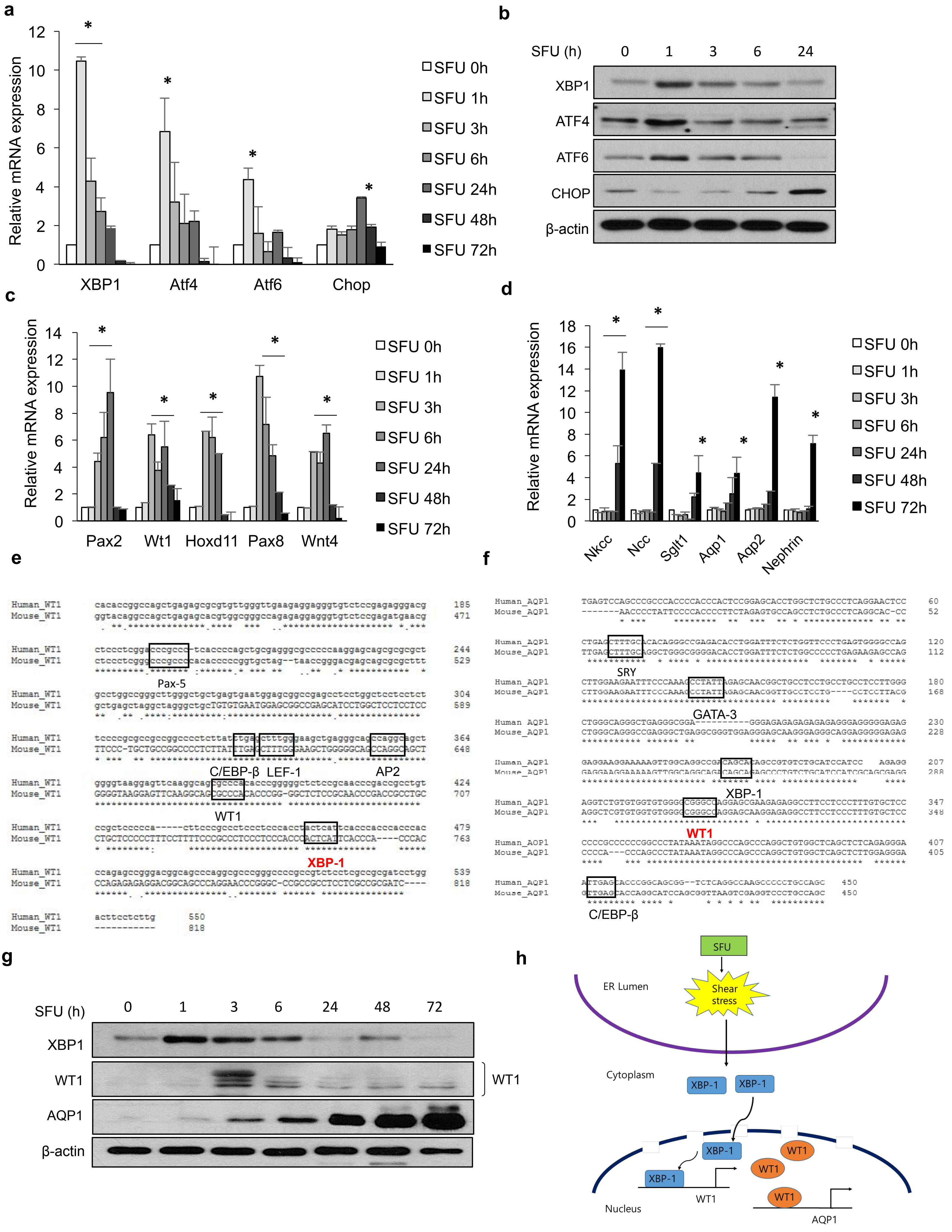
a
Relative mRNA expression
12
10
8
6
4
2
0
XBP1
Atf4
Atf6
Chop
SFU 0h
SFU 1h
SFU 3h
SFU 6h
SFU 24h
SFU 48h
SFU 72h
b
SFU (h)
0
1
3
6
24
XBP1
ATF4
ATF6
CHOP
β-actin
c
Relative mRNA expression
14
12
10
8
6
4
2
0
Pax2
Wt1
Hoxd11
Pax8
Wnt4
SFU 0h
SFU 1h
SFU 3h
SFU 6h
SFU 24h
SFU 48h
SFU 72h
d
Relative mRNA expression
18
16
14
12
10
8
6
4
2
0
Nkcc
Ncc
Sglt1
Aqp1
Aqp2
Nephrin
SFU 0h
SFU 1h
SFU 3h
SFU 6h
SFU 24h
SFU 48h
SFU 72h
e
Human_WT1
Mouse_WT1
Pax-5
C/EBP-β
LEF-1
AP2
WT1
XBP-1
f
Human_AQP1
Mouse_AQP1
SRY
GATA-3
XBP-1
WT1
C/EBP-β
g
SFU (h)
0
1
3
6
24
48
72
XBP1
WT1
AQP1
β-actin
WT1
h
SFU
ER Lumen
Shear stress
Cytoplasm
XBP-1
XBP-1
XBP-1
WT1
XBP-1
WT1
WT1
Nucleus
WT1
AQP1

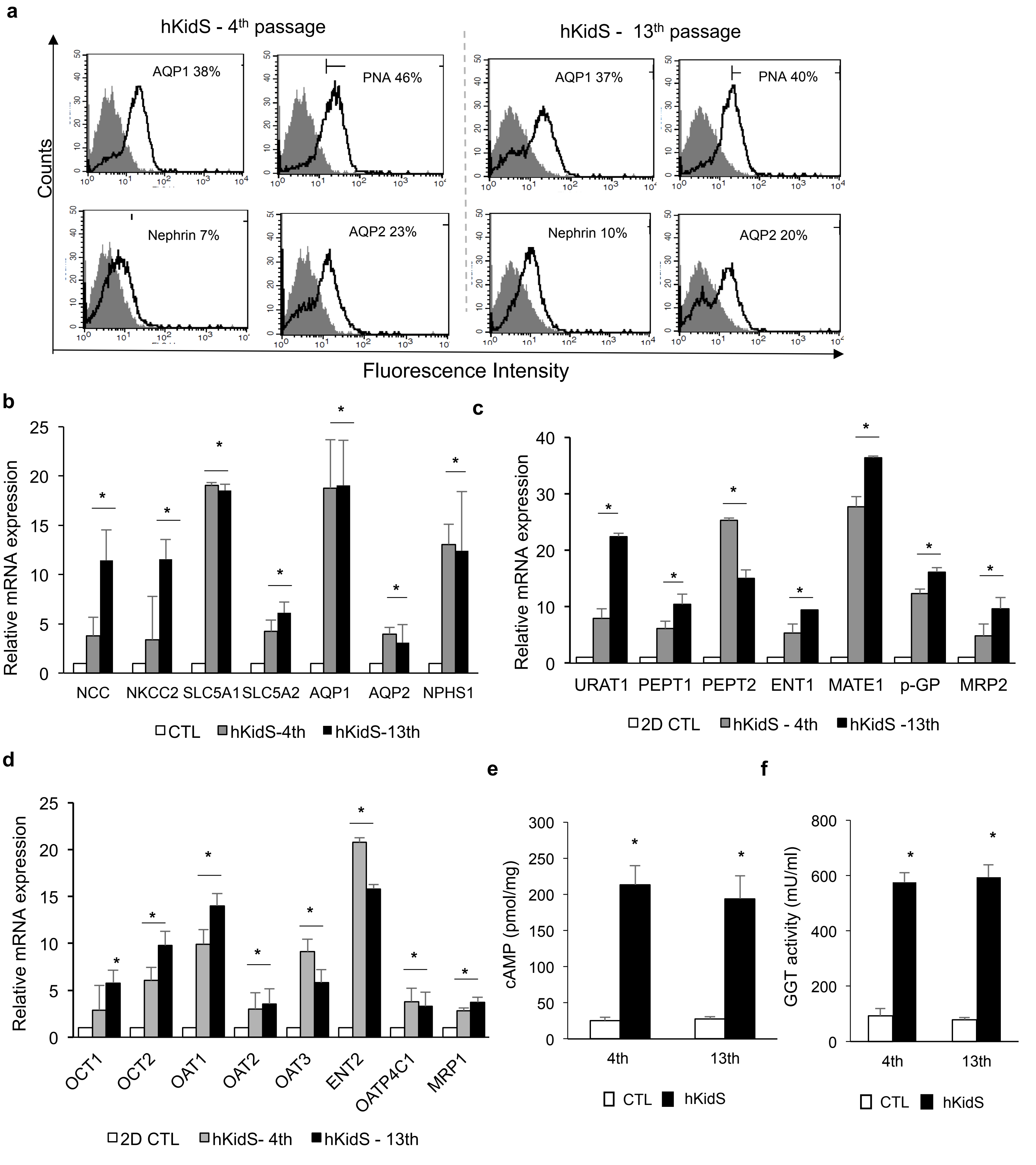
a
hKidS - 4th passage
hKidS - 13th passage
AQP1 38%
PNA 46%
AQP1 37%
PNA 40%
Nephrin 7%
AQP2 23%
Nephrin 10%
AQP2 20%
Counts
Fluorescence Intensity
b
Relative mRNA expression
NCC
NKCC2
SLC5A1
SLC5A2
AQP1
AQP2
NPHS1
CTL
hKidS-4th
hKidS-13th
c
URAT1
PEPT1
PEPT2
ENT1
MATE1
p-GP
MRP2
2D CTL
hKidS - 4th
hKidS -13th
d
OCT1
OCT2
OAT1
OAT2
OAT3
ENT2
OATP4C1
MRP1
2D CTL
hKidS- 4th
hKidS - 13th
e
cAMP (pmol/mg)
4th
13th
CTL
hKidS
f
GGT activity (mU/ml)
4th
13th
CTL
hKidS

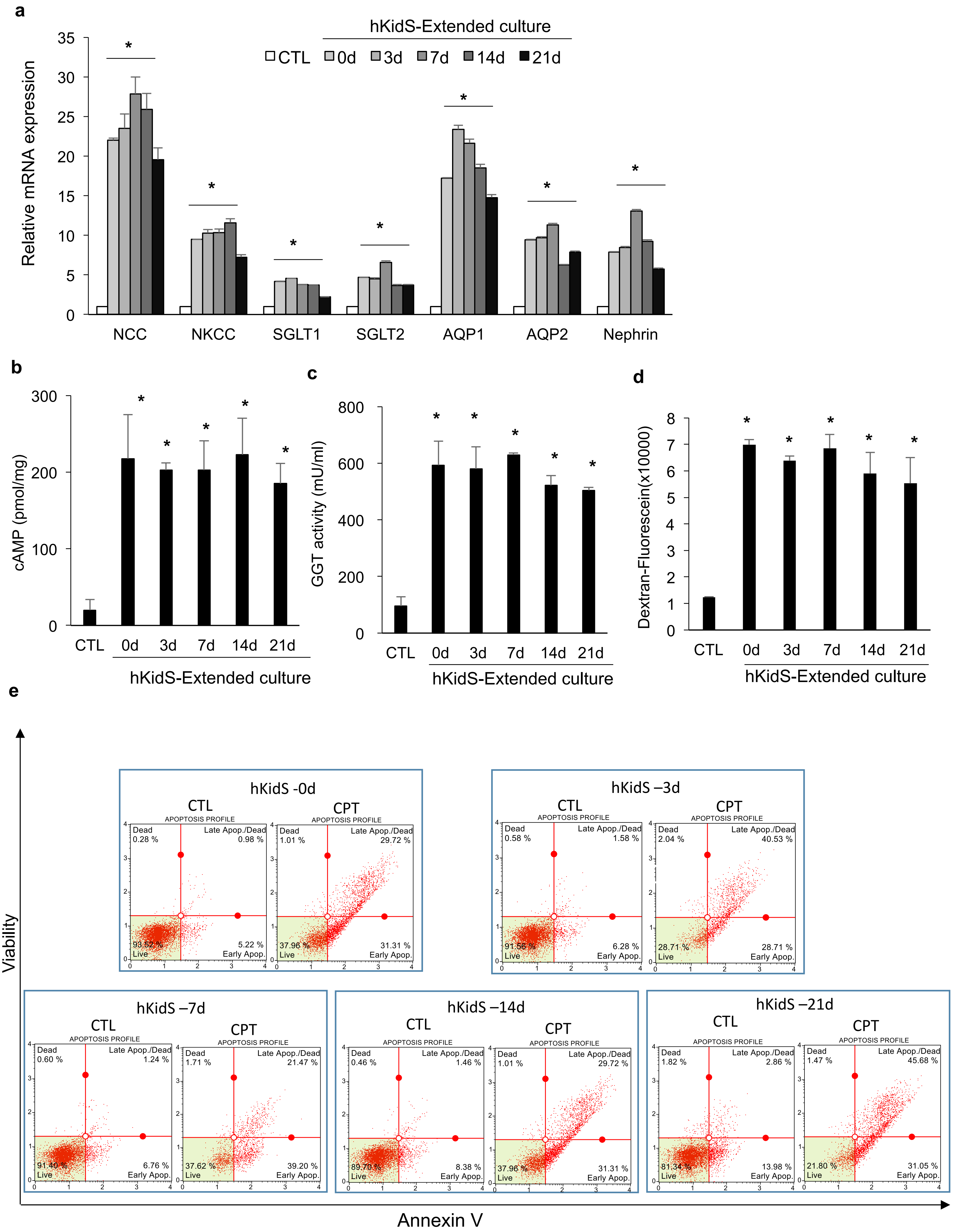

a
hKidS-Extended culture
CTL 0d 3d 7d 14d 21d
Relative mRNA expression
NCC NKCC SGLT1 SGLT2 AQP1 AQP2 Nephrin
b
cAMP (pmol/mg)
CTL 0d 3d 7d 14d 21d
hKidS-Extended culture
c
GGT activity (mU/ml)
CTL 0d 3d 7d 14d 21d
hKidS-Extended culture
d
Dextran-Fluorescein(x10000)
CTL 0d 3d 7d 14d 21d
hKidS-Extended culture
e
Viability
Annexin V
hKidS -0d
CTL
CPT
hKidS –3d
CTL
CPT
hKidS –7d
CTL
CPT
hKidS –14d
CTL
CPT
hKidS –21d
CTL
CPT
APOPTOSIS PROFILE
Dead
Late Apop./Dead
Live
Early Apop.

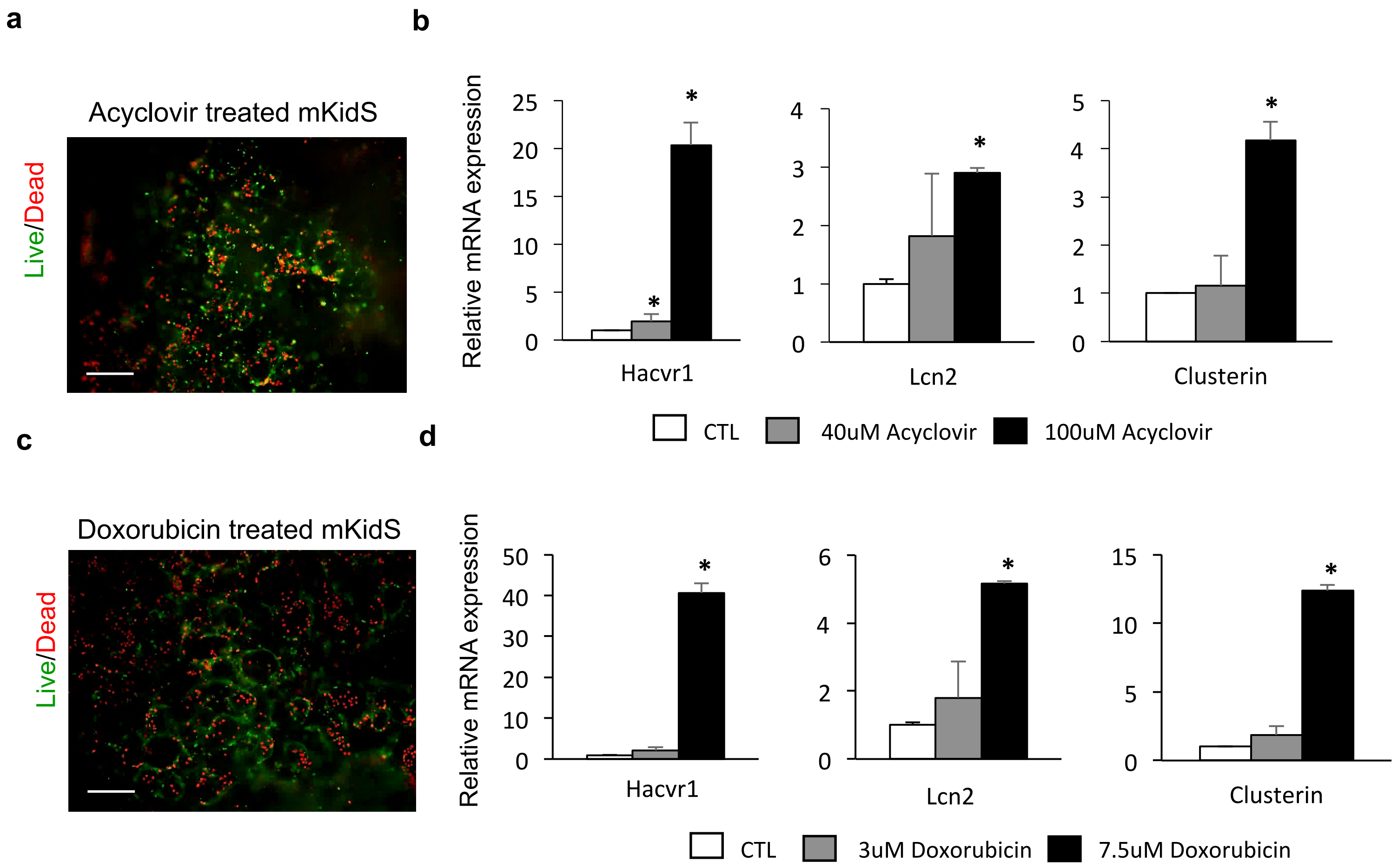
a
b
Acyclovir treated mKidS
Live/Dead
Relative mRNA expression
Hacvr1
Lcn2
Clusterin
CTL
40uM Acyclovir
100uM Acyclovir
c
d
Doxorubicin treated mKidS
Live/Dead
Relative mRNA expression
Hacvr1
Lcn2
Clusterin
CTL
3uM Doxorubicin
7.5uM Doxorubicin

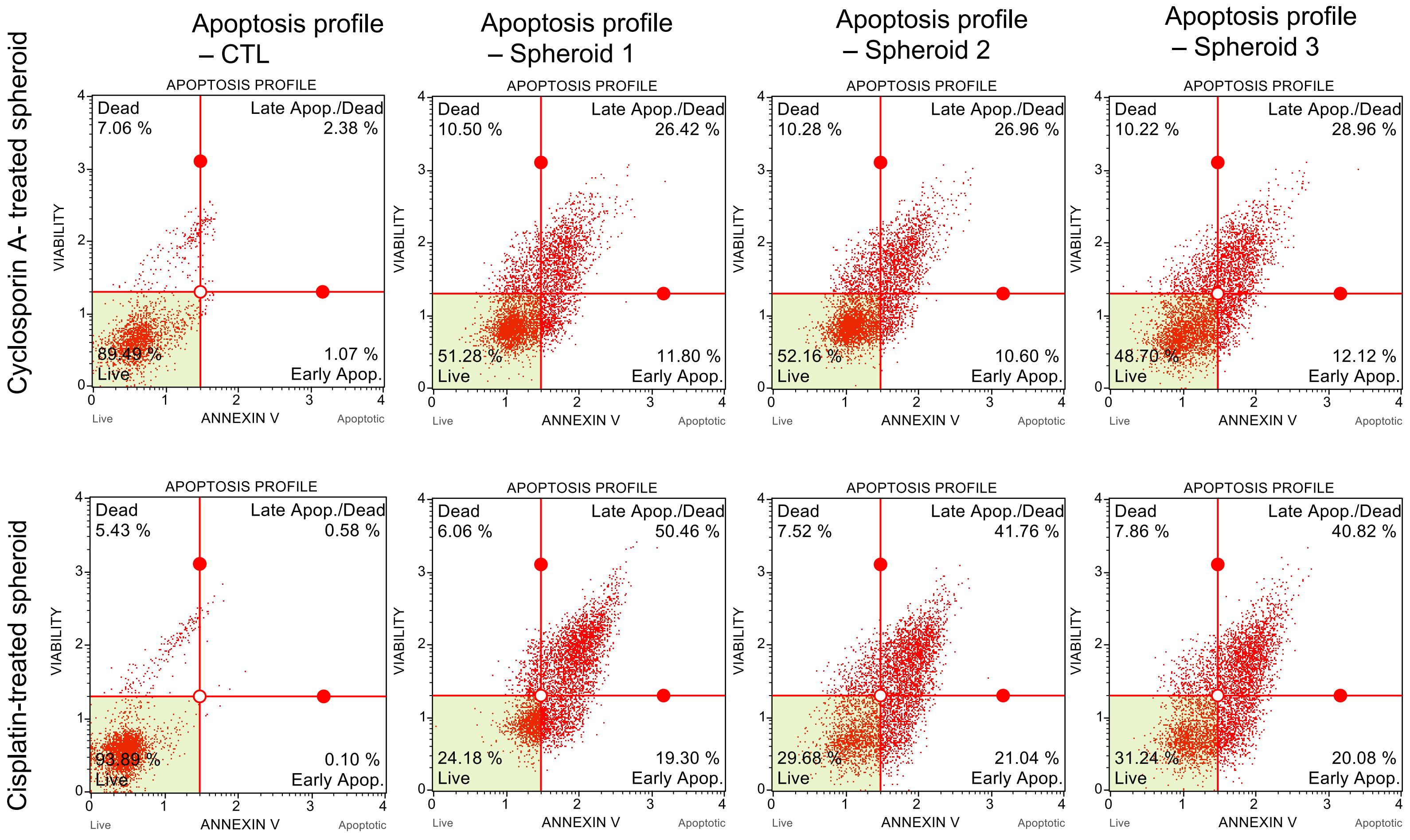

Cyclosporin A- treated spheroid
Cisplatin-treated spheroid
Apoptosis profile – CTL
Apoptosis profile – Spheroid 1
Apoptosis profile – Spheroid 2
Apoptosis profile – Spheroid 3
APOPTOSIS PROFILE
VIABILITY
ANNEXIN V
Live
Apoptotic
Dead 7.06 %
Late Apop./Dead 2.38 %
89.49 % Live
1.07 % Early Apop.
Dead 10.50 %
Late Apop./Dead 26.42 %
51.28 % Live
11.80 % Early Apop.
Dead 10.28 %
Late Apop./Dead 26.96 %
52.16 % Live
10.60 % Early Apop.
Dead 10.22 %
Late Apop./Dead 28.96 %
48.70 % Live
12.12 % Early Apop.
Dead 5.43 %
Late Apop./Dead 0.58 %
93.89 % Live
0.10 % Early Apop.
Dead 6.06 %
Late Apop./Dead 50.46 %
24.18 % Live
19.30 % Early Apop.
Dead 7.52 %
Late Apop./Dead 41.76 %
29.68 % Live
21.04 % Early Apop.
Dead 7.86 %
Late Apop./Dead 40.82 %
31.24 % Live
20.08 % Early Apop.

Fig. 1j

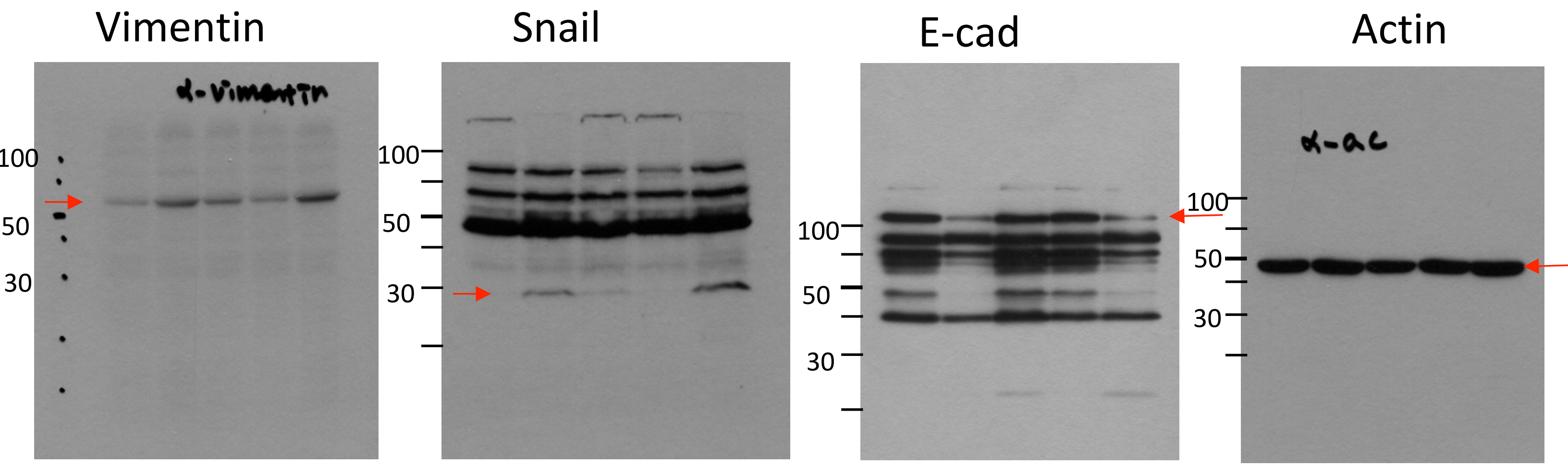

Fig. 2c

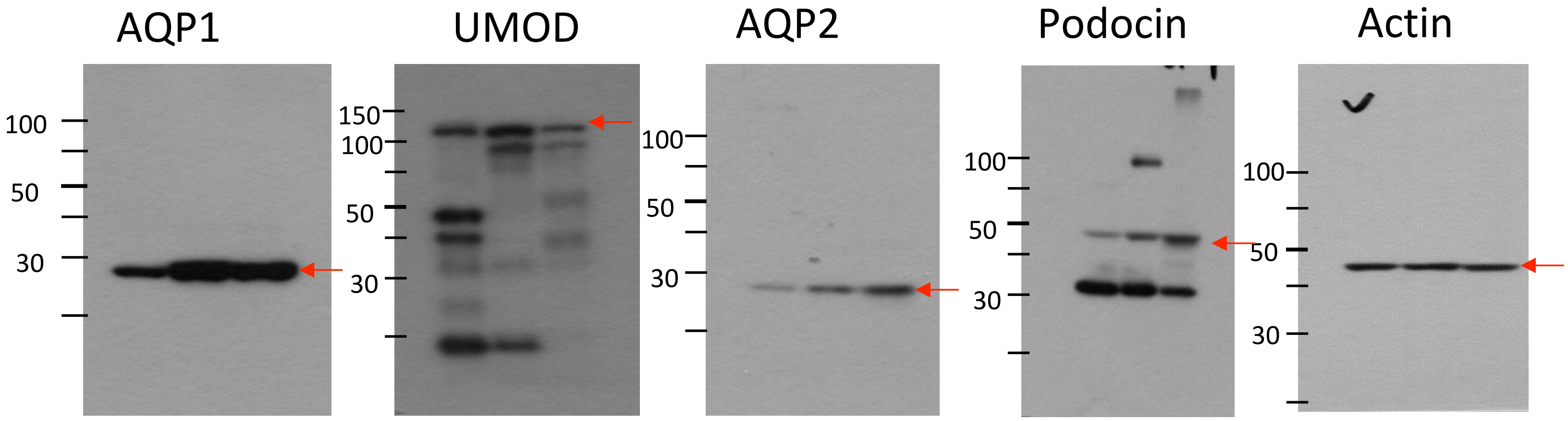

Fig. 3b

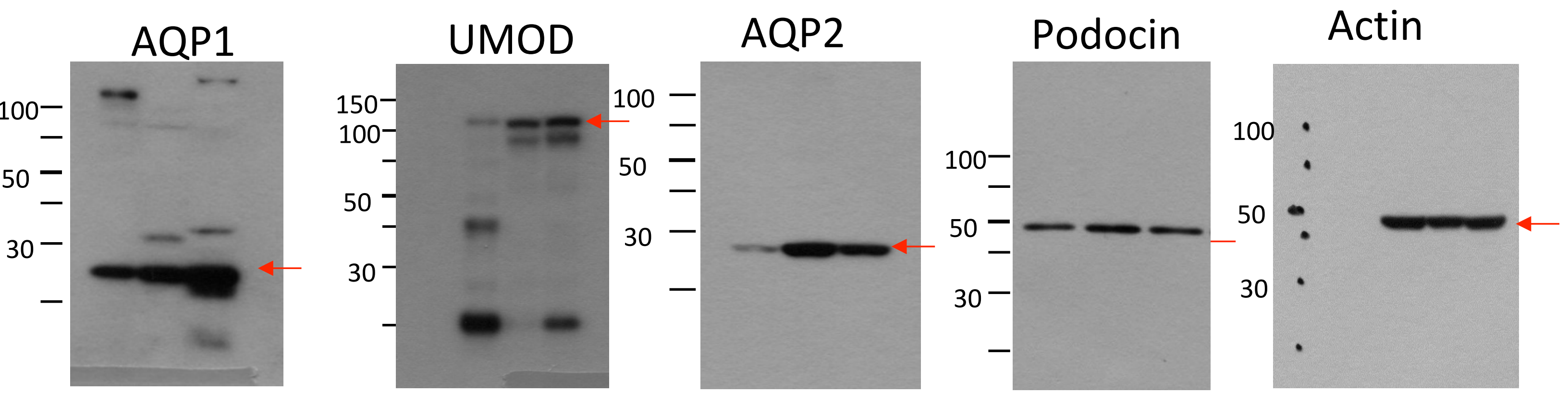

Fig. 5c

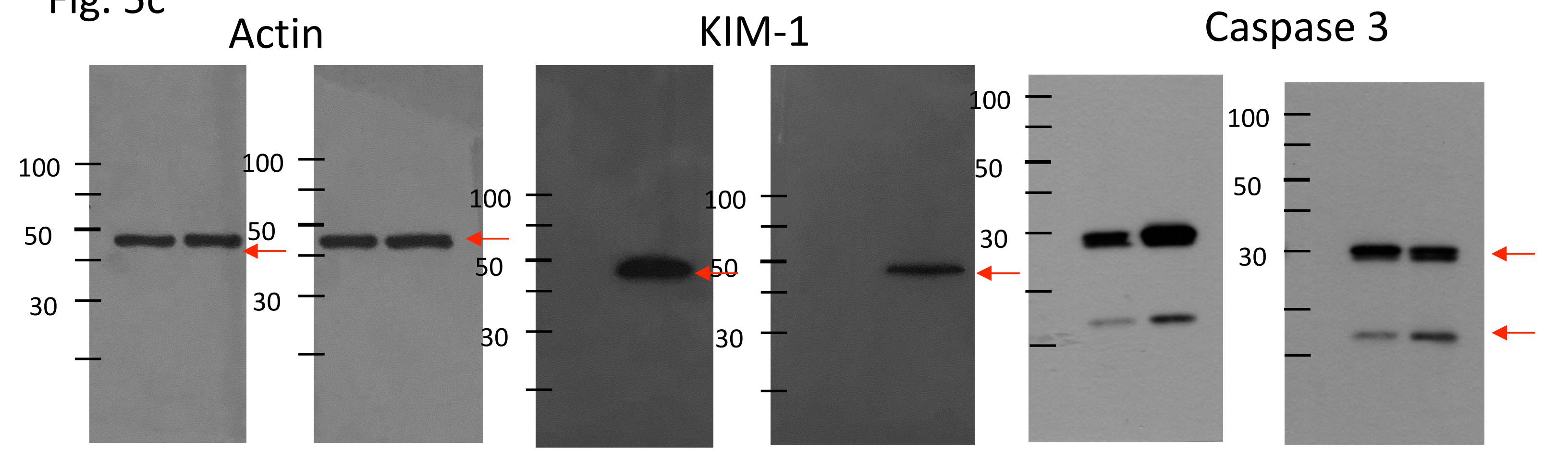

Fig. 7c

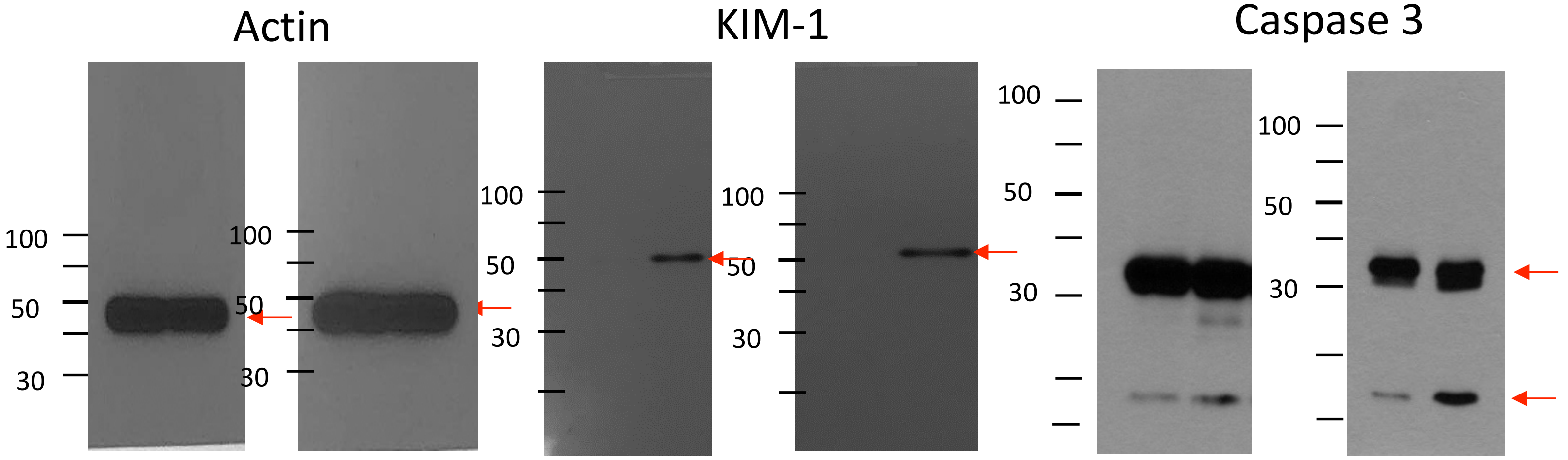

Supplemental Fig. 7b

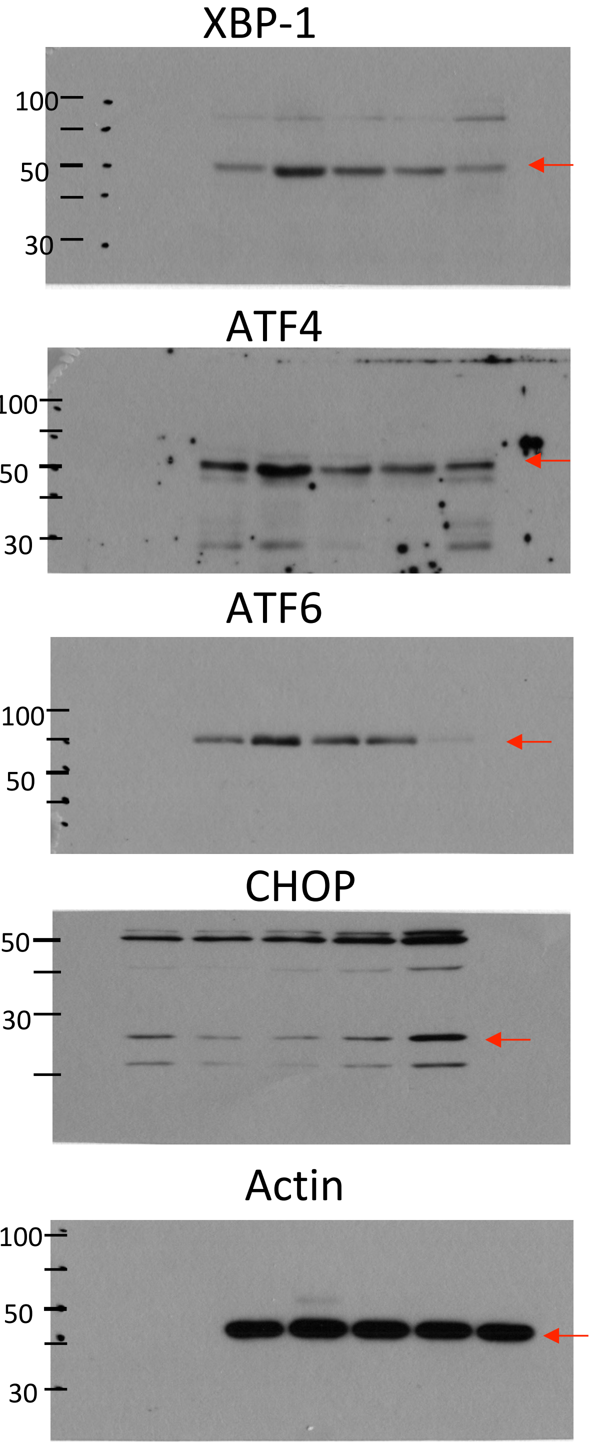

Supplemental Fig. 7g

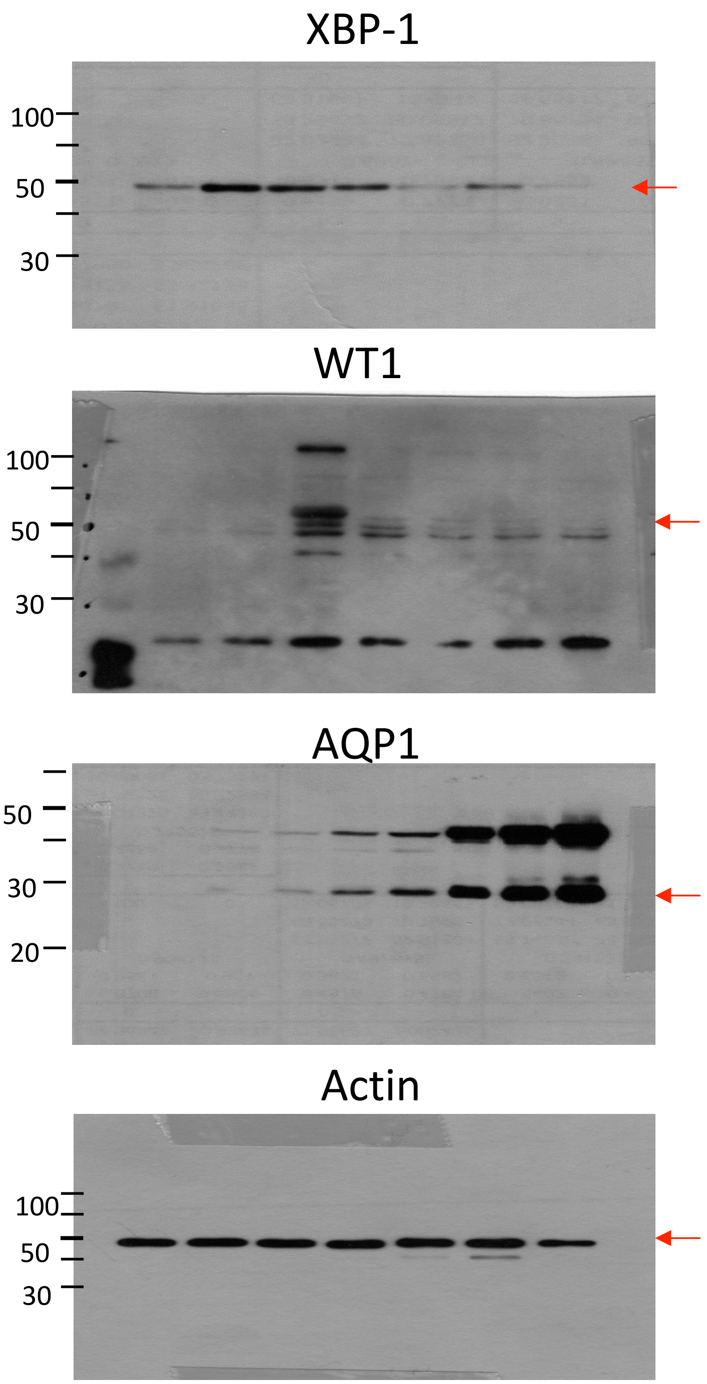

Full-length blots

Supplemental table I. Sequences of oligonucleotide primers used for qPCR

Gene name	Forward	Reverse
mNcc	CCTCAAGCAGGAAGGTAGCC	TCACTGTTGGTGCCTGTCTC
mNkcc2	GAGAAGCGGGAATTGGTCTTG	CTCCACCTCCACGAACAAAC
mAqp1	GGCCTTTGGTTTGAGCATCG	CCGGAGGATGCTGATCTGAC
mAqp2	TTCGAGCTGCCTTCTACGTG	GGCTGTTGCATTGTTGTGGA
mSglt1	CTGCAGACATCTTCTCCGGG	GGCAGTGATTGCTAGCAGGA
mPodocin	CCGCTGCATTGAGAATGGAC	CGGCCTTTGCCTTCTTGTCA
mNephrin	CGAACTGATGCATGCAAGAAGG	TACACTAGTGCGTGGTCTCT
mMate 1	CATGGAACGCACGGAGGAGT	CATCATCAGCTGGGCCAAGAA
mUrat1	CGATGTTCTTCTGGCCGTCT	TGGTCGTAAACCCAGCCATC
mOatp4c1	AATCAGAATGGGGGTTCGCA	GAGACACTGGGGGTGAAAGC
mEnt1	CGGGTTTGAAAGCGCTCG	GGTGACTGGTTGTCATGGCT
mEnt2	TCTCAGGCTCCAACTCAGGA	CCGTCAGCCAGATCTTCCG
mOat1	CTGATGGCTTCCCACAACAC	GTCCTTGCTTGTCCAGGGG
mOat2	CAACTGCGGAATCTGGTGCT	ATCAGGCAGGGCACAATGATG
mOat3	ATGACCTTCTCCGAGATTCTGG	GTGGTTGGCTATTCCGAGGAT
mOct1	GACGCCTGGAAAGTGGACC	GCAACATGGATGTATAGTCTGG
mOct2	CCAGTGCATGAGGTATGAGGT	CTGAAACAGGTCCAGCATCCA
mPept1	CCGGCACACCCTTCTAGTG	TGGCGTTGTGACTGGTGAC
mPept2	AAAGCGACAACATTGGCTAGA	AAATCCCAAATCGCCATCCAT
mp-Gp	GGACTTTGCAGAGGAAACCG	TGTCCAGCCAACCTGCATAA
mMrp2	CTGGAAATCACGATGGACGA	GAAAGCCCAAGGGAATCCACA
mMrp1	GCACCTATGCCAACGCTGAG	AGACGAGTTGCTGAGATGCC
mHavcr1	GCTGCTACTGCTCCTTGTGA	TCTTCAGCTCGGGAATGCAC
mLcn2	GCTGTCGCTACTGGATCAGA	TGGCGAACTGGTTGTAGTCC
mClusterin	CCCAAAGGGGGTGTACTTGA	GAATCTTCATGGCGTGGCCT
mCd133	CCCGGAGGAGAGCTGGACCG	AGGGCAATCTTCAGAGCCAGACT

mSox9	GTGCAAGCTGGCAAAGTTGA	TGCTCAGTTCACCGATGTCC
mFoxd1	CCCCTCCTGGACTAACCGGGC	CGAGGTGTTTGCGCTCCCCG
mLgr4	CCGCTGCCTGCTTGCCTGAA	TCCTGGTGACACGCCGCTTC
mPax2	GCGAGCCGACACCTTCAC	GACGCTCAAAGACTCGATCCA
mPax8	CAGAAGGCGTTTGTGACAATGA	TGCACTTTGGTCCGGATGAT
mLgr5	CACAGCCACTGCGGCGACTT	CAATGGGCGTCTGCCGGGTC
mSix2	GGACGGATCGTTGTGACTCAGGA	TCGCTGTTCTCCCTTTCCTGGC
mBmi	CTCCAAGATGGCCGCTTGGCT	TTGGAGCCATTGGCAGCATCAGC
mWt1	CTCATGAACCTTCACAACAACGA	TGGCGCATCTCAGAGAACAC
mScf	AAGCAACGGCCAAGGACGGG	GCGGTGTGGCGACTCCGTTT
mc-kit	GCCAAGGGCATGGCGTTCCT	TCCGCCCGTGAGTGAGGAGG
mCd90	AGTCTCGGGCGCGAATCCCA	TGCCGCCACACTTGACCAGC
mCd105	CTCAGGGCCCGTCCACACCA	AGGGGCGTGGGTGAAGGTCA
mCcnd1	TCAAGTGTGACCCGGACTGC	CCTTGGGGTCGACGTTCTG
mCcnd2	TAAGGGAAGCACTCCCCGA	CACTCGGTCCCGACTGTAA
mCcne1	GTTCCAAGCCCAAGTCCTGA	CTGGAGCGGACTGAAAGGTC
mctnnb1	GCCGCGCCGCTTATAAATC	CTTCACAGGACACGAGCTGA
mc-myc	GTTGGAAACCCCGCAGACAG	ATAGGGCTGTACGGAGTCGT
mOct4	GGCTTCAGACTTCGCCTTCT	TGGAAGCTTAGCCAGGTTCG
mSox2	CAGTAGACTGCACATGGCCC	CTTCTCCAGTTCGCAGTCCA
mRB1	AGCCCAGAGAGGATTGGGTA	GGTGGTGTCTCATACGCACA
mNanog	AAGGATGAAGTGCAAGCGGT	GGTGCTGAGCCCTTCTGAAT
mPCNA	TACAGCTTACTCTGCGCTCC	TTGGACATGCTGGTGAGGTT
mVim	GATCGATGTGGACGTTTCCAA	ATACTGCTGGCGCACATCAC
mFn	ACAAGGTTCGGGAAGAGGTT	CCGTGTAAGGGTCAAAGCAT
mHoxd11	ATGCGCTGTTGTTCCTCTCC	CTAGCCCCATGGCCTAACTT
mEya1	GAATGCAACAAGCCACAGCC	CCGTCTTGATGCCATAGGAG
mLhx1	CAACCTGACCGAGAAGTGCT	CGAACCAGATCGCTTGGAGA
mXBP1	AACACGCTTGGGAATGGACA	ACATAGTCTGAGTGCTGCGG

mATF4	TGGGGCCTTTAGGACGATCT	GTGGTCACGTGATCCTACCG
mATF6	TGGTTAGTAATGGGAATGGAAGC	CTTGGTCCATCGTGGGAGG
mChop	CAGAGCCAGAATAACAGCCG	TGGACCAGGTTCTGCTTTCA
mGapdh	TAACATCAAATGGGGTGAGG	GGTTCACACCATCACAAAC
hNCC	CTTCTGCATGCGCACCTTTG	GTGTCTGCCTTCCTGCTTGA
hNKCC	CTGCGATAGCAACTAACGGGT	ACATAGCAACAGCCACTGCAT
hSGLT1	CCCATTCGCAGGACAGCTCTTA	GGGAGCAGGGCAGTAGCG
hAQP1	TACGACTTCATCCTGGCCCC	ATTTGGGCTTCATCTCCACCC
hAQP2	CTCCCCTAGACATGGCCTCT	ATACACACACGTGAAGGCGT
hPodocin	AAGAAGTCAAAGGCCGGGAG	TCCACATTCACTACCGTGGC
hNephrin	GTGCAATGGGCCAAAGATGG	GCACTCATACTCCGCGTCAT
hMRP1	CCCGCTCTGGGACTGGAA	TCGGTCATGTCGGGAGAGAT
hMRP2	GGGAACTGGTGAGTCTCCCT	CCATTCTGATGTTCTCCGTAAAGG
hSLC22A1	CCCCTCATTTTGTTTGCGGT	AAGGTTCTCGGCGTCCTTC
hSLC22A2	TGCAGCTGGAGTTCTCATGG	CTCCGATATCTCCGCCCAAC
hSLC15A1	GAGCGGCTCATCTCCCAAAT	CCCGGACATAGTTGTTGCCT
hSLC15A2	TGATCAAGTCCTTGGGTGCC	CACCAAAAGCTGCCACACAG
hSLC29A1	CAGCCCGGAGAAGGGACG	TTTTAGGCCCGGGAGCCA
hSLC22A6	AGTATGGAGGTACTCCGGGC	GCATGGAGAGGCAGAGGAAG
hSLC22A7	CACCACGTTGGGGGAAGAAA	ACCAGATCCCACTCAGTTGC
hSLC22A8	GGACACCAGAGTCCATACGC	CCTTTCTCCCTCTTCTTGCCA
hSLC22A12	GAACACGGGCACTCTCCTGA	CAGTCCCGCACACCGTAGG
hSLC29A2	TTCTCCAGCGTCCCCGATA	CCGGGAGTGCTCTCATAATCT
hSLCO4C1	GGTTGGTCCTTGAGGCGAC	CCGCCTGAACCTTCCTACTC
hP-gp	GAGAGCGGAGGACAAGAAGG	AATGTTCTGGCTTCCGTTGC
hHAVCR1	GCTCTGAAGCCGTTATATGTGG	GGTTGGTACCCTCCACCAGA
hLCN2-F	CACCCTCTACGGGAGAACCA	ACACTGGTCGATTGGGACAG
hClusterin-F	CACTCGACTCTGCTGCTCAT	CAAAATGAAGGCATGCCGGG
hTUBB	GTGGTACGGAAGGAGGTCGAT	GGCGGAACATGGCAGTGAAC

Supplemental Table II. The list of differentially expressed genes when 2D cells and mouse kidney spheroids (mKidS) were compared.

tracking_id	2D cells-1	2D cells-1	mKidS-1	mKidS-2	mKidS-3	mKidS-4	mkidney-1	mkidney-2
0610040J01Rik	0.455924	0.451682	12.2687	18.9776	8.03338	12.0805	33.2355	42.6219
1110008P14Rik	31.8528	28.3732	108.661	70.8353	109.519	99.4178	97.9958	91.2505
1110032A03Rik	1.72103	2.31066	4.622	5.69935	4.70756	4.58296	44.2251	39.6742
1110054M08Rik	1.91527	1.94416	6.2986	4.44433	4.51838	5.42653	27.282	26.523
1190007I07Rik	6.46972	7.23491	17.4532	11.8152	19.8546	15.7638	12.2972	13.4246
1300002E11Rik	4.32375	4.69464	15.5261	11.3389	15.5066	12.7375	18.3929	21.6805
1600002K03Rik	5.20493	6.51885	18.1339	14.2029	20.058	17.3657	19.1382	7.41541
1700001F09Rik	0	0	0.955719	0.856908	1.71111	0.80774	0	0
1700007K13Rik	0.904437	1.09599	5.3813	2.59328	5.13707	5.76206	2.29335	0.703986
1700009P17Rik	0.567888	0.802369	3.93599	3.22047	2.70238	4.04619	15.1499	10.8579
1700010I14Rik	0.262717	0.247258	1.45368	1.26663	1.5399	1.50644	0.425922	1.04747
1700011H14Rik	3.8526	4.09125	43.6534	38.798	39.1638	39.1417	356.706	311.511
1700020I14Rik	5.8683	7.17784	16.7302	15.4849	16.7928	15.8457	15.8707	15.0287
1700037C18Rik	1.85193	2.3256	5.23055	3.45187	5.91066	5.93682	20.3971	17.3515
1810043G02Rik	4.46548	4.98337	13.8818	10.1779	12.0701	12.5599	25.5828	19.6347
1810043H04Rik	18.8214	19.0992	54.6824	29.2369	55.6395	51.1168	88.8974	59.4932
1810058I24Rik	33.9916	39.5783	81.8699	56.6068	80.4997	81.4546	133.967	123.554
2010111I01Rik	3.35395	3.70341	8.60896	8.39636	11.1739	10.046	45.3929	33.7263
2010320M18Rik	6.55763	6.78683	18.9317	10.4527	14.529	12.7249	32.8692	39.0564
2200002D01Rik	84.9367	81.7148	203.284	83.5675	227.833	187.137	78.2952	55.4541
2210016F16Rik	2.44364	2.53053	17.1992	15.2755	19.5051	17.1449	24.2342	23.5095
2310007B03Rik	16.4774	16.6449	1.39626	1.08957	2.09504	1.54701	5.59699	5.75977
2310009A05Rik	7.67822	8.09887	24.5848	17.8937	23.2899	19.2154	51.113	46.5066
2310022B05Rik	32.2403	28.694	13.5857	12.6667	12.1856	12.6229	14.4577	12.3638
2310069B03Rik	3.16834	3.583	0.897005	0.799861	0.669998	0.688575	0	0
2410004I01Rik	1.72188	1.4747	0.0578877	0.0973209	0.194367	0.366882	0	0.189887

2410006H16Rik	461.896	474.542	316.77	122.805	216.82	229.17	31.5944	26.9819
2410018L13Rik	0.0660142	0.0310749	1.20638	1.07567	1.80158	1.89115	0.196003	0
2510002D24Rik	8.61661	8.3673	19.522	16.1967	18.5197	18.2731	16.2079	15.7113
2610528A11Rik	1.18883	0.593307	40.64	6.79245	2.86805	14.0427	0.826996	1.01526
2610528J11Rik	6.20647	5.17491	13.8355	15.7286	12.2094	12.7484	89.7903	73.1263
2700094K13Rik	102.597	93.4989	267.415	131.815	264.362	239.027	20.9119	14.0185
2810008D09Rik	10.8917	11.5387	32.1343	13.5023	37.2139	32.8591	53.5104	30.7438
3300005D01Rik	11.4695	10.1129	0.844558	0.316177	1.94179	0.949749	0.562409	0
3830406C13Rik	2.04623	2.22419	7.19854	6.95726	6.9581	6.60282	20.3309	17.1444
4930426D05Rik	0.0281254	0.0794232	2.0679	1.52406	1.26851	1.15039	7.965	7.35224
4930461G14Rik	34.521	30.8299	11.9375	8.65409	9.51407	10.565	23.4376	36.3355
4930503L19Rik	6.09396	7.12735	2.3392	3.16467	2.57369	2.80531	1.14337	1.12495
4933412E12Rik	10.5959	9.88703	3.10827	4.4524	3.0045	3.166	7.4137	5.33462
5430416N02Rik	48.2648	53.3455	22.4528	9.94007	21.0605	18.4423	3.71844	1.95501
5830416P10Rik	8.31736	7.47952	1.88927	1.1083	3.97998	2.6051	0	0.0582638
8430408G22Rik	3.56237	2.41545	16.2238	15.7555	16.7043	16.3774	4.65272	2.98777
9030617O03Rik	1.03906	1.11542	10.737	9.60976	11.4734	10.1794	33.1412	30.6244
9030625G05Rik	1.58505	1.71253	0.465256	0.130369	0.347222	0.545974	0.276399	0.339598
9130008F23Rik	11.6453	11.9427	4.46839	2.30753	4.90545	4.52437	2.56038	1.19953
9330182L06Rik	3.79272	3.32699	0.727248	0.993673	0.702045	0.89517	0	0.0334764
9930111J21Rik1	1.4983	1.51816	0.0487784	0.166497	0.0303652	0.0514667	0.259869	0.121194
A230050P20Rik	1.97612	2.19391	11.6236	11.6563	11.0768	11.117	13.3459	11.4495
A330074K22Rik	1.75796	1.81957	0.544425	0.621225	0.196114	0.451419	0	0
AA467197	2.18352	4.20165	1.36081	0.408444	0.760504	0.513868	0.515407	0.948521
AI506816	4.52073	4.41047	0.978829	0.822299	1.0917	1.55498	0	0
AW112010	0.0677202	0.0638337	20.7646	18.9074	13.6329	16.2188	7.61001	10.0807
Aars	127.365	129.3	51.9246	63.3788	58.9452	56.543	39.0665	40.9052
Aatk	3.92613	4.0296	0.628878	0.332318	0.161149	0.341336	1.01668	0.47652
Abcb1b	16.6006	15.8731	6.76329	6.08582	9.12958	7.44307	2.49869	2.21619

Abcc3	0.505753	0.44402	2.26367	3.19579	1.93203	2.24084	39.0534	44.7753
Abcd4	11.0693	9.86218	4.97361	3.86007	4.54489	4.90544	14.6107	8.22584
Abcg2	19.1804	17.7668	6.97428	8.11794	8.8828	8.65154	142.521	90.1515
Abhd14b	6.72559	6.30204	18.7407	15.1148	15.249	16.3103	431.601	434.192
Acaa1a	19.7063	20.5793	46.7798	36.7237	41.0673	43.7045	85.3581	67.8726
Acot1	0.852766	0.664339	4.68833	4.05107	4.12121	3.98686	22.3603	23.5168
Acp5	2.89004	3.63117	8.81739	10.7347	7.9498	10.6609	82.793	56.9319
Acsbg1	17.2425	20.4778	5.77912	2.89507	7.29155	6.22621	0.279298	0.17177
Acsl4	16.6405	16.6252	46.4361	68.3865	47.4687	50.9924	7.56044	8.27783
Acsl6	26.3059	24.867	3.96889	2.80965	4.28521	3.816	0.101342	0.18708
Actn1	195.822	182.512	70.3015	105.192	99.0042	90.9135	13.1158	16.7203
Acyp2	0.0818928	0.231694	3.18827	1.99053	3.25882	3.56315	36.2669	42.7239
Adamts5	11.3774	11.2029	4.41683	4.34798	3.90965	4.23483	6.63433	4.32622
Adamts9	0.326291	0.306909	1.38851	3.10908	1.23849	1.78051	2.35694	2.5184
Adck4	4.56226	4.10328	11.1664	9.46347	9.50972	10.5356	12.1253	12.2543
Adm	0.674363	0.666646	17.3289	9.19944	5.52136	9.30537	8.00834	4.6759
Adora1	0.973268	0.797235	4.1136	6.80007	3.61708	4.2561	4.04112	10.0839
Adssl1	11.9298	13.5972	6.60184	5.64414	4.08835	6.05691	7.18035	12.3237
Aebp1	0.188968	0.177697	1.17188	1.56126	0.988241	1.11417	38.3701	34.3318
Aff1	15.1425	16.0085	4.97843	9.66463	6.11493	6.46664	8.4607	12.0994
Ak1	0.850962	1.26982	7.54744	4.39795	9.77536	8.34583	27.8778	40.6357
Akr1e1	8.14476	8.06444	24.2657	21.4839	19.3805	20.6085	38.5016	40.9628
Akr7a5	10.4367	11.2012	29.6481	22.639	28.4199	27.4568	198.181	172.257
Akt3	13.0539	13.0515	5.3251	7.60347	5.40593	5.61826	3.34736	3.03969
Alcam	5.58776	6.19215	24.7672	36.1099	26.6278	28.1137	4.50765	4.79813
Aldh18a1	88.6711	86.498	16.843	22.986	18.4457	19.3714	2.17087	1.86977
Aldh1a1	1.22661	1.46355	0.0727068	0.101882	0.217249	0.179052	10.4189	5.28514
Aldh1a3	5.61375	10.4559	14.5325	14.8341	21.7454	17.4238	2.6281	4.01809
Aldh1l1	0.0139851	0.0526304	1.57724	1.57318	1.0399	1.10954	206.326	184.897

Aldh4a1	3.94044	3.03311	18.1896	26.5087	14.2212	17.2975	77.4438	73.0658
Aldh6a1	2.37578	2.89962	5.90633	11.7348	4.456	6.0374	106.783	96.1907
Alg10b	28.4978	27.1541	9.44575	14.5642	10.4888	10.3806	1.90923	3.10368
Alkbh6	9.97679	10.1452	24.5394	18.8062	20.7785	20.663	32.8382	40.9143
Alox15	0	0.0170958	1.32633	1.85858	1.35124	1.56936	0	0.132918
Amd1	0.0151179	0.0141796	5.30819	0.0139964	5.9433	0.0175018	0.0900749	0.0556068
Amotl2	69.3266	61.4704	13.2808	16.3171	19.1684	17.4741	21.0516	24.2663
Ampd3	4.56818	4.48861	12.3104	15.8553	6.77394	10.3062	12.6749	15.4755
Anapc11	17.9832	18.3377	46.7493	32.5935	46.3342	42.8421	15.068	16.8858
Anapc13	80.1083	83.0056	207.134	139.934	184.553	186.706	616.38	595.661
Ank	4.0427	3.95309	6.82582	11.5012	9.27551	9.0171	4.81283	6.6382
Ankrd1	757.773	714.166	90.5139	49.4869	136.489	101.159	1.82434	3.7386
Ankrd11	34.261	33.732	7.65075	16.1663	8.47148	9.76815	7.74603	7.5146
Ankrd37	6.52301	5.20052	46.7756	19.8154	24.2338	28.302	1.32079	2.83898
Antxr2	37.124	44.5142	14.74	16.1477	22.1339	18.7241	3.25104	4.79098
Anxa2	669.818	620.569	1379.47	1027.71	1540.42	1368.8	124.045	169.557
Anxa8	0.763819	0.965986	13.234	8.77237	16.9881	13.9417	1.27703	0.872392
Aoc1	0	0	0.91851	2.66511	0.423869	0.836234	2.71298	6.07795
Ap2s1	118.512	111.393	286.736	212.33	280.808	279.182	214.546	187.148
Ap4s1	2.47051	3.10231	8.07197	6.38177	7.90767	7.25587	27.5062	18.1633
Apbb1	8.58337	8.62899	3.19233	2.73981	1.77627	2.20043	13.8394	14.805
Apcdd1	0.728664	0.568891	3.4803	3.40192	2.93989	3.5821	0.922497	1.36163
Apip	11.0159	12.5772	27.5782	20.6512	30.055	25.8251	23.7871	23.2956
Apln	13.2052	11.0075	1.78534	0.814168	1.78064	1.30913	9.44717	9.61734
Apobec1	2.55626	3.24945	0.464601	0.647722	0.471042	0.653769	2.02188	3.35401
Apobec3	10.387	10.9129	30.3478	29.0309	30.1596	29.5665	5.42607	7.83106
Apol9a	0.125119	0.117787	16.0599	25.4608	19.7343	20.0287	3.53005	2.92468
Apol9b	0.307608	0.307158	17.8483	22.462	19.2775	19.3228	2.74069	5.61551
Apoo-ps	0.000942551	0.67456	2.62498	3.58955	2.32611	4.2468	7.69E-60	6.38E-05

App	36.6696	33.3615	138.787	220.804	119.75	146.415	311.348	365.2
Aqp1	9.96172	7.28508	0.487292	0.263386	0.604655	0.661132	366.595	679.539
Arg2	2.29496	2.60125	32.1742	58.8927	25.0275	32.5964	30.6155	45.7759
Arhgef10	5.38483	5.29406	1.70757	2.37371	1.21537	1.44685	3.28588	3.75966
Arhgef18	17.0454	15.4659	6.22925	8.97799	7.06228	7.62723	21.655	22.3384
Arhgef5	56.2499	53.627	21.3906	27.1778	22.9309	23.6465	14.4042	14.1038
Arl4c	55.4209	61.9895	24.0345	18.6826	31.2508	26.0724	2.87525	2.63326
Arl4d	5.08722	5.89969	17.9116	13.8348	10.741	13.3949	24.8054	37.6223
Arnt2	3.82195	4.41253	1.19684	1.61529	1.67515	1.5361	5.75271	4.67661
Arrdc3	1.55885	1.16337	10.2235	14.8646	8.21547	10.3383	16.0872	21.9986
Asap1	29.081	26.6786	10.6676	14.9899	12.0508	12.1179	3.5381	4.00167
Asns	131.772	142.478	36.3518	41.0817	37.6791	37.3647	7.17683	8.99491
Aspa	1.0152	0.871341	4.95913	10.7844	7.0364	7.46879	48.4226	33.363
Ass1	0.524362	0.975488	1.71689	5.76062	3.8373	3.60847	61.4844	17.0723
Atf3	42.3909	50.6283	8.54668	6.78649	8.93233	8.41392	1.07418	0.82558
Atf4	375.067	428.052	201.117	142.947	212.703	193.888	98.2434	110.827
Atf6	8.70424	9.06902	3.39897	4.54897	3.66568	3.92417	5.85373	6.13871
Atl1	3.75567	3.69293	1.25955	1.43112	0.724918	1.22136	1.28223	1.07023
Atox1	108.637	96.4371	299.128	174.33	281.401	283.066	472.882	340.248
Atp1b1	18.9726	16.7291	59.074	122.723	53.3173	69.2629	824.336	1212.64
Atp5e	145.555	133.175	463.836	228.143	432.871	390.314	1912.96	1412.69
Atp5j	102.012	100.039	243.378	144.038	227.266	215.478	902.897	763.161
Atp6v0d2	0	0	0.966335	2.32369	0.974668	1.20333	33.4721	25.6344
Atxn2	25.9063	25.2603	10.0676	14.5719	10.5939	12.4107	14.3206	13.8381
Atxn2l	54.0055	51.2701	21.1356	32.6178	22.8523	24.1089	34.385	25.1447
Aven	8.17188	7.93084	21.4003	13.6866	26.49	23.0486	14.8376	12.0601
B2m	232.058	265.832	628.059	1005.37	673.766	709.127	1051.48	891.375
B3galnt2	5.41838	6.14136	12.3585	12.4583	14.6945	13.1851	6.6079	4.67991
B3galt4	1.91449	2.4575	6.19835	5.69711	5.54034	5.83123	3.61897	2.22463

B4galnt1	26.4841	24.8625	54.8944	61.2144	59.97	61.0063	22.0043	31.7071
B4galnt3	26.2403	22.0917	3.95589	6.01416	4.66909	4.82176	6.69103	4.06173
B4galt6	5.75698	5.02779	17.5684	30.2289	20.9224	21.9283	5.16263	7.27835
B930041F14Rik	0.785816	0.564351	2.49375	2.17361	2.61494	2.24555	2.82839	3.10025
B9d1	25.1793	22.5403	64.9678	61.1375	64.7272	65.9946	23.2472	27.0913
B9d2	4.51674	5.61242	12.6827	8.64559	13.4812	12.1571	28.4837	23.4465
BC002163	0.000934154	0.0051502	64.0268	38.2327	16.5688	57.8542	74.7741	97.8633
BC005624	24.3341	25.8998	54.3819	49.4954	55.2602	51.7661	18.2149	34.9986
BC026585	0	0	1.33447	0.509939	0.9963	0.640453	123.712	135.411
BC030500	0.824757	0.428331	3.21155	5.50596	4.02093	4.3208	0	0
BC031181	34.7313	36.1828	89.8978	67.7052	103.125	86.7553	70.2052	77.9378
Bag1	84.4091	84.4291	199.662	157.436	202.946	198.569	381.667	460.795
Basp1	435.007	432.019	205.931	153.494	232.108	204.52	3.02213	3.00829
Bax	67.4613	66.1627	170.248	98.135	195.371	167.64	78.0111	58.3365
Baz1a	26.5254	25.3002	9.07612	14.0416	13.3493	12.3561	1.37244	1.51483
Bcap29	18.1112	19.1895	10.2878	6.11558	9.68346	8.98505	10.4713	11.0361
Bcl2l12	7.40794	6.50927	17.2282	13.3039	17.4371	17.1898	9.64272	6.41777
Bcl2l15	2.44999	3.06074	6.33386	4.53977	14.6745	9.43876	0.536794	0.577587
Bcl3	10.5602	10.8845	27.957	28.3151	27.1303	28.6786	7.71887	6.626
Bdkrb2	2.91854	3.61194	0.29914	0.955689	0.469172	0.726258	0	0.0987188
Bex1	88.5151	79.1416	27.2251	27.659	35.7961	32.488	6.26776	4.91791
Bex4	1.60657	2.27149	8.03116	7.81292	9.48266	9.58695	50.2857	34.2726
Blnk	6.49713	7.2893	19.1299	22.9347	25.5288	24.2179	16.3598	17.4878
Bloc1s1	47.302	44.753	117.067	82.3479	113.897	111.594	472.816	347.649
Bmf	0.784903	0.798432	3.69186	4.92775	1.48136	2.67125	3.31516	4.44638
Bmp8b	5.89998	5.24648	1.36662	1.10801	1.787	1.68243	0	0.0538487
Bmyc	0.732224	1.03506	11.5173	13.4963	14.402	12.6698	13.0021	17.0758
Bnip3	27.4488	31.8644	208.341	93.29	49.7643	92.1784	80.879	73.4098
Bola2	85.1987	86.3605	205.913	110.55	241.403	212.177	319.711	236.45

Brcc3	15.6203	13.9088	32.9658	29.0538	29.8721	31.1815	19.4756	17.339
Brsk1	11.3747	11.2576	3.92746	3.04223	4.19509	3.91589	1.94948	1.27862
Bscl2	3.89026	3.54805	11.0544	7.78976	10.4209	9.59934	70.389	67.7803
Bst2	94.1873	95.4602	519.493	439.722	524.073	510.893	54.4801	53.1585
Btn1a1	1.67808	1.81258	0.261099	0.219535	0.261861	0.261	0	0
C1ql1	5.04929	4.64729	0.925452	0.353639	0.80081	0.710674	0.225312	0.276916
C230052I12Rik	4.56394	4.74651	9.71191	9.54956	11.1623	10.5752	4.79577	4.42263
C3	1.55104	1.45897	56.1841	189.744	44.7163	78.6041	3.46994	2.79603
Cacna1g	1.32042	0.972233	0.062534	0.0902332	0.152987	0.0834088	1.12708	0.773311
Cacnb1	2.65063	2.34928	0.945983	0.590769	0.605891	0.613223	3.93325	5.32595
Cad	31.3554	26.1235	10.3065	13.8029	15.3639	13.6593	2.57638	2.48467
Calcb	0.516483	0.486567	12.6054	11.6558	17.8921	14.5859	0.306038	0
Cald1	175.655	157.042	22.2298	22.649	29.8562	25.5198	22.3404	27.7928
Caly	0	0.0474488	0.949833	1.31508	0.687917	1.36844	0	0
Camk1	35.0452	43.8486	19.388	9.68355	15.3453	17.4095	50.8318	47.3954
Camk2b	0.608542	0.613108	3.52635	3.20604	3.70758	3.37887	1.53503	1.25897
Cand2	5.88202	6.07662	1.89977	2.66471	1.41353	2.05821	2.66292	3.50231
Capsl	1.79244	3.17869	8.07168	8.75171	8.05185	8.64984	1.87405	1.91839
Car2	19.0894	14.53	47.9697	45.1577	52.7131	48.044	345.767	339.754
Casp12	0.117185	0.26778	2.62907	3.54924	2.03347	2.26641	2.19114	1.16376
Casp4	0.355862	0.548218	7.16566	10.5725	5.4978	6.77067	1.92122	1.77128
Cav1	49.2852	43.1696	16.3632	12.5144	17.1628	15.3975	36.1305	21.4898
Cbl	5.72019	4.74122	0.989465	3.80552	1.30442	1.68187	0.543066	0.728896
Cbr2	0.528554	0.543137	19.2906	27.8182	10.3197	15.4778	21.9324	10.6734
Cbr3	0.491822	0.501757	13.7183	14.0528	10.325	12.7594	3.89045	2.53989
Ccdc125	2.04765	2.5568	5.59959	5.36565	5.5788	5.16954	2.33481	2.64841
Ccdc23	14.1299	12.3977	32.9805	20.798	31.5325	31.0454	30.5596	32.2259
Ccdc28b	3.36107	3.80092	13.2309	11.1213	8.04691	12.3305	24.2345	18.4337
Ccdc88c	7.21728	6.50694	2.04334	4.2088	2.38154	2.84583	3.13006	1.8525

Ccl2	0.837177	1.39599	9.58399	10.3401	17.0425	14.5104	1.5241	0.935706
Ccl5	4.33202	8.93348	28.9955	25.6462	37.4886	33.7758	14.7548	4.93252
Ccng1	27.2825	35.1891	85.9486	68.4986	108.64	92.8379	48.0395	42.4108
Ccnjl	2.10306	1.93346	4.98885	5.16486	5.253	5.80783	0.573113	0.704926
Ccrn4l	34.3285	34.1623	15.0615	10.6381	15.2788	14.4988	6.94378	7.15215
Cd14	7.65964	9.06402	91.2552	101.52	66.7715	84.4339	8.0395	9.09717
Cd200	0.0374569	0	1.13941	1.09733	0.742903	0.918239	13.5905	13.906
Cd24a	219.324	207.102	594.591	596.139	554.854	553.186	151.444	282.496
Cd47	9.05161	10.9182	29.6874	25.8955	25.52	25.2148	9.59406	8.54894
Cd82	32.0756	34.9188	137.432	131.178	146.761	138.307	124.172	209.725
Cd83	0.868975	1.26557	4.12613	3.11928	4.00364	3.98809	1.10732	1.06271
Cdc42ep1	38.6173	40.3145	17.7724	16.3172	17.8513	17.5916	32.0017	22.3838
Cdc42ep2	3.5537	3.17088	8.99954	8.63047	7.97249	7.83898	16.3355	18.2769
Cdc42ep3	11.2581	9.42034	1.80835	2.11165	3.12373	2.88944	7.15208	9.20978
Cdh10	2.17905	2.74915	0.111401	0.0234169	0.0624348	0.117546	0	0
Cdh3	34.7592	31.0418	12.9626	19.1827	15.1558	15.3726	2.3207	2.39204
Cdh6	7.40091	7.24405	9.11882	27.5736	12.1693	13.8352	1.67459	2.24144
Cdk14	6.81278	7.60449	2.76294	3.49672	2.12883	2.46424	1.33017	1.29035
Cdkl1	1.12824	0.834403	8.74234	14.5	4.46393	7.56457	66.0872	97.7422
Cdkn2d	6.49831	7.37751	19.7892	10.9987	13.8219	15.9426	12.7831	8.66834
Ceacam1	0.461313	0.222527	12.5426	19.1995	9.29357	11.4119	12.9747	13.5766
Cebpa	0.165199	0.279789	1.80967	2.62824	0.962743	1.85182	13.5709	11.4909
Cebpb	20.5305	22.3769	46.5389	72.1916	58.0272	57.554	8.45393	9.72962
Cebpd	1.92333	2.4458	5.03997	10.0386	6.94637	7.38011	18.6662	22.2972
Cenpm	11.7371	11.1025	38.7775	24.1069	27.7815	31.0531	0.771997	0.576281
Cep170	12.7522	13.1501	4.89857	6.10566	5.79812	5.66637	1.57881	1.19206
Cers3	1.40196	1.67982	0.0584917	0.0368852	0.213076	0.0925785	0.0787191	0.145251
Cetn2	18.3383	17.8751	47.7334	41.4392	44.8648	44.3333	53.687	47.504
Cetn4	1.4256	1.86253	4.84417	4.2865	3.48211	4.09284	13.0882	17.0863

Cfh	0.00971041	0.00913448	1.55624	3.73606	0.842208	1.47209	50.591	57.5104
Cfi	0.0857231	0	1.92062	2.61157	0.717367	1.20121	7.64586	5.01469
Chac1	58.6849	59.4407	6.48773	7.00115	4.66424	4.32728	1.03916	1.70342
Chac2	4.34164	4.99856	11.6869	12.5118	11.649	11.2649	16.9295	20.004
Chd1	24.3262	23.1665	8.4421	12.5176	10.2661	9.81248	4.38296	3.77517
Chdh	0.516799	0.326394	5.00977	7.0379	1.98539	3.94789	32.5978	41.4648
Chil1	0	0	6.21479	4.975	5.06356	4.95935	0	0.294404
Chka	55.4289	54.029	21.2166	15.4385	26.488	22.8471	49.1323	36.8264
Chst13	0.0275541	0.129681	0.854078	1.38481	1.29851	1.35233	0.32745	0.503245
Chst8	0.0183072	0.0344531	0.830241	1.992	0.907722	1.0685	5.98933	10.1118
Chst9	0.10217	0	1.35803	2.66394	1.05623	1.71256	1.21327	3.47982
Ciart	12.4327	14.9058	5.1315	2.88638	2.64628	3.46883	2.37506	1.82469
Cib1	26.715	26.3927	96.5158	71.1274	105.775	94.8245	210.386	165.527
Cisd1	33.2383	28.0167	74.2026	49.9481	76.535	70.8426	220.736	220.6
Cited1	0.407676	0.19214	7.42148	3.80841	1.77545	3.35163	10.4482	10.3741
Cited2	78.5828	78.1379	16.1817	23.2655	18.0469	17.8734	46.6391	67.3805
Cited4	0.600709	0.424225	2.49592	5.66533	1.86336	2.6354	8.69031	8.48989
Ckb	37.3582	30.9675	17.315	7.61261	20.7883	17.6591	317.537	517.286
Ckmt1	1.35179	1.43491	3.78929	3.53377	4.53018	3.86561	75.0142	86.343
Clca4	39.4097	45.5387	8.50729	7.33425	7.84438	7.33901	0	0
Clcn5	6.42999	5.98155	1.34805	3.60203	2.26741	2.44076	2.27927	1.69063
Cldn12	43.0192	43.1351	17.788	20.3188	21.0969	18.5907	18.3259	17.546
Cldn19	0.0200376	0.00942468	1.4419	3.21517	1.35779	1.86322	63.7151	106.705
Cldn2	0.0562885	0.0529581	2.6918	5.16759	1.77885	2.61053	67.1648	72.0076
Cldn23	3.3247	3.58591	1.128	0.858043	0.872633	1.02051	0.144253	0.266068
Cldn4	350.886	400.635	189.742	141.088	204.784	185.957	16.6978	22.3487
Cldn9	0.192424	0.27173	1.74033	1.52282	1.59126	1.19987	0.190463	0.234134
Clec2d	0.430461	0.184249	8.28269	12.2126	4.80733	6.41061	42.9595	35.3879
Clec2f	0	0	1.59381	1.33949	1.99125	1.09033	0	0.305186

Clic6	0.0570138	0.0536355	1.2479	0.837284	0.847777	0.98419	0.135763	0.0835078
Clip2	12.4702	11.1708	4.77558	5.88217	5.35306	5.1484	8.62929	8.02433
Clip3	6.50936	6.58943	2.25977	2.10577	2.19414	1.88325	2.16964	1.90638
Cluap1	5.61345	6.25456	14.9344	13.5688	15.6643	15.5308	20.9292	24.5806
Cmpk2	0.809882	0.660356	4.93984	11.6322	5.63724	6.69271	6.10549	8.94331
Cndp2	21.8575	17.5241	48.1255	56.3114	53.5111	52.0674	233.706	191.125
Cnnm4	9.40779	9.79629	3.52033	4.08394	4.18666	4.54312	4.56292	5.51228
Cnpy2	36.5467	49.2511	106.282	90.9636	95.7162	96.8726	134.791	114.269
Cntn2	7.47753	7.31892	0.451463	0.218076	0.403885	0.273594	0.0777049	0.0159361
Coa3	62.6216	60.5943	197.695	153.087	189.359	184.87	187.908	226.574
Coa4	5.4266	4.99785	19.4458	17.266	17.1599	17.5799	11.6792	17.0311
Col4a2	46.3592	38.0577	14.4498	21.2398	16.6279	17.9456	40.5172	37.0438
Col4a3	4.24088	4.17471	0.655624	0.900877	0.770963	0.719755	38.2002	29.2322
Col6a1	0.203375	0.201389	2.14156	1.59175	1.76401	2.03486	13.4463	13.0533
Col7a1	5.67778	6.03142	1.59431	0.775025	2.19541	1.68188	0.967264	0.562049
Col8a1	25.4516	23.84	1.7312	1.17984	1.72631	1.62459	0.392799	0.302047
Commd3	40.3539	44.0453	106.898	71.4867	111.538	95.7198	65.7879	60.2854
Coro6	3.60974	3.65439	0.683146	0.675663	0.495035	0.721298	0.215294	0.529284
Cotl1	40.7252	46.0741	22.9112	16.3405	21.0843	19.1532	141.953	90.6128
Cox14	55.6014	53.7503	141.906	73.6257	118.511	110.008	125.747	123.964
Cox16	3.41561	3.48758	8.84326	5.17131	8.74628	8.49924	11.4148	8.73229
Cox17	90.6765	97.6149	240.418	145.445	238.206	219.699	382.771	347.232
Cox20	11.1364	15.1576	43.5305	27.2171	45.9517	35.723	257.561	204.547
Cox6b2	2.91444	3.36101	25.8905	8.43611	21.3086	15.0775	33.2105	18.2724
Cox7a1	3.17167	4.60419	10.9399	4.99272	8.20533	10.5985	230.119	181.951
Cp	0.839405	1.47817	32.0286	66.9634	29.5261	36.3037	22.8285	33.0822
Cpm	9.28856	7.30384	15.4691	28.7328	10.7849	14.7705	6.51637	10.0663
Cpn1	1.10741	1.01861	6.07258	4.70736	4.08835	5.67467	83.9203	49.5706
Cpne8	31.2882	30.3516	11.2542	11.8033	14.9278	11.5237	6.89867	9.48433

Cpped1	7.29238	7.75377	16.9252	15.1124	17.8744	17.0973	21.5964	27.64
Cr1l	6.09914	6.04989	17.4697	13.5673	17.8071	15.5004	24.2153	24.5774
Crabp2	14.7436	16.3744	4.35506	2.92862	4.7343	3.48343	0.996018	0.815569
Creb3l4	0.324636	0.611188	2.7448	3.18671	2.56223	2.27664	1.05193	1.83209
Creb5	4.85525	4.92282	0.633599	0.771055	0.859636	0.73897	0	0.110365
Creg1	2.62831	2.86599	6.64652	8.20763	7.73222	7.6967	35.1041	32.4159
Crim1	20.2607	18.1563	3.61549	4.92385	4.74636	4.38485	11.3263	15.4985
Crip1	1.49028	2.67641	64.0365	41.7471	58.581	55.1083	71.6078	50.2505
Crip2	31.6573	32.9835	89.5012	81.5646	121.239	107.955	111.963	134.581
Crisp1	0.0655358	0	3.22992	4.30194	5.04032	4.17624	0	0
Crispld2	0.348007	0.27923	5.5097	12.3189	5.31396	7.03078	5.66752	5.99782
Crmp1	2.0829	2.26119	0.112739	0.108333	0.19857	0.186948	0.086662	0.106609
Cryl1	3.81953	3.34793	10.1381	8.79905	8.20993	8.85519	151.926	101.429
Csdc2	1.52877	1.30622	6.04439	6.63442	6.38183	7.01447	2.71786	2.50707
Csf1	48.4895	45.4299	10.9758	18.2735	14.9702	14.8519	6.28757	6.12987
Csf1r	6.99349	7.33414	1.00887	0.531449	0.654019	0.833619	39.4745	42.1597
Csf2	40.4144	46.7702	3.20502	1.45753	3.94066	3.38502	0	0
Csn3	3.31757	3.74207	0.759919	1.27777	1.45426	1.57095	0	0
Csnk1e	100.69	92.0023	31.34	34.4666	38.7308	37.7548	9.33671	10.0522
Csrnp1	10.0693	7.82436	3.08186	2.22901	3.15702	3.18213	4.19072	3.07092
Cstb	167.558	198.379	489.593	357.239	489.438	443.855	124.468	116.907
Ctgf	433.509	400.529	88.9189	70.9392	142.147	113.817	32.27	26.5928
Cth	9.98046	14.0079	4.26338	3.9801	4.52844	3.8572	141.537	158.221
Ctla2b	12.7202	18.1036	6.79489	2.66864	10.2701	5.94861	2.95584	1.92633
Ctsh	17.3294	15.9156	61.721	38.0096	49.1138	50.2187	124.658	161.675
Ctsw	1.38481	3.33243	0.213123	0.286667	0.286386	0.315053	0.913121	0.140279
Cttnbp2nl	45.5659	48.1736	14.659	16.4877	16.3784	15.7918	3.84508	5.62566
Cutc	2.09655	2.90445	7.23454	4.79569	6.57425	7.02984	9.80002	11.9839
Cwh43	3.55836	3.04643	0.567229	0.566345	0.397327	0.430223	13.2562	13.3725

Cxcl1	245.612	260.087	87.8605	65.5296	117.831	92.8083	0.622281	0.573306
Cxcl17	0.0694539	0	7.09981	6.81067	3.71156	3.67001	0	0
Cxcl2	8.28297	9.40373	24.0752	23.9524	37.0117	31.8737	0	0.16312
Cxcl5	15.9275	19.8517	43.0763	45.1864	56.6007	49.0006	0	0
Cxx1a	1.18287	0.904943	4.70975	3.82168	5.13669	4.88636	20.185	16.7191
Cxx1b	1.19248	1.05044	4.94445	3.79834	5.34588	4.81581	28.3068	21.4628
Cyb5	60.3776	60.1883	158.102	130.24	184.215	164.762	1316.99	978.422
Cyld	10.4186	11.0453	3.87179	4.59562	4.37985	4.34517	5.04515	5.2342
Cyp1b1	0.484675	0.517722	4.67539	3.02285	4.4273	4.51125	6.16413	7.3733
Cyp24a1	0	0	4.49651	5.768	2.55234	3.48241	4.11207	1.62249
Cyp26a1	0.278575	0.309869	2.49464	4.52462	1.72718	2.18936	1.05363	0.462689
Cyp2j6	0.0118961	0.0783396	0.907415	0.884601	1.22353	0.888067	22.0941	17.7277
Cyr61	691.982	623.58	35.4605	37.9207	69.0167	52.3451	9.78871	15.4883
Cys1	0.434308	0.312585	3.42289	5.09972	1.35419	3.22847	66.4939	61.9335
D10Jhu81e	11.8431	12.1815	29.0041	25.8817	23.9066	25.7786	136.162	172.117
D330041H03Rik	1.4882	1.17386	7.71468	0.561952	9.16759	5.18978	75.4104	71.2048
D630039A03Rik	0.029902	0.0140669	1.19035	1.69587	0.500302	0.854835	18.5943	12.9133
D630045M09Rik	22.5421	19.7352	2.15077	1.20542	2.94393	2.69139	17.9272	21.7723
Daam1	9.39833	9.66319	3.66456	4.87977	3.79544	3.82378	4.41167	4.67098
Dab2	5.28741	4.53761	1.77837	1.73046	1.82641	1.62034	109.19	75.3158
Dad1	119.794	111.946	256.161	186.269	288.646	249.268	309.904	279.317
Dalrd3	13.7963	13.8935	31.6781	31.3951	32.9365	33.9532	76.0232	51.2264
Dapk1	6.61501	6.01285	13.8267	22.0679	13.0534	15.2323	14.2	14.4304
Dbi	264.996	258.136	830.703	515.428	727.818	688.074	946.668	948.047
Dbndd1	8.37145	8.89145	4.41298	2.86782	4.24817	3.62185	0.357079	0.346413
Dcdc2a	0.988741	1.04817	4.21576	7.03713	2.55492	3.8483	3.32425	4.73494
Dclk3	6.35423	4.78001	2.47693	1.63879	2.50333	2.42786	15.5959	19.4096
Dcxr	4.86857	6.03018	40.7314	31.1257	33.4909	38.539	277.688	158.404
Ddah1	56.2438	50.2323	18.345	17.7962	18.9125	18.1605	56.0075	42.7499

Ddi2	22.9382	20.0989	6.29015	14.3324	8.26045	8.79259	5.858	3.66777
Ddit4l	0.814893	1.18768	3.76374	2.77825	4.27765	4.21411	8.55627	10.7581
Ddt	21.8007	20.7447	66.3661	58.0491	69.9701	61.6996	209.11	182.054
Ddx58	5.51264	5.55471	13.1183	25.2167	15.7427	17.5219	9.40125	11.5353
Ddx60	0.0489894	0.0724105	1.10587	2.24338	0.745636	1.34618	0.291787	0.4103
Def8	20.585	18.3298	7.82077	9.55662	8.75577	8.57114	29.3229	25.9949
Derl3	0.035024	0.032976	1.08633	1.46776	0.477965	0.778293	1.45552	0.894532
Dhh	16.3493	14.4065	3.75649	4.12487	5.52091	4.26761	1.3246	1.28971
Dhrs3	4.66512	4.79712	145.079	124.461	85.3362	108.879	251.317	211.447
Dhrs4	10.215	10.281	33.1081	27.4385	31.7392	32.773	93.2078	79.7409
Dhx58	0.436427	0.410665	5.69221	11.9394	4.62087	6.81371	1.83887	2.39466
Dmpk	2.04561	2.31022	7.36348	5.58178	6.16113	6.42168	12.263	10.251
Dmtf1	26.4895	28.0713	12.3059	13.054	11.1649	10.5574	13.8906	10.1637
Dnajc15	27.3056	25.836	60.5772	36.8578	66.751	58.4915	163.694	146.437
Dnajc22	2.08544	1.81453	0.454889	0.088253	0.391937	0.406348	16.8897	16.8408
Dnajc30	6.69363	7.39299	17.123	13.6914	15.7511	14.8312	53.4345	50.5301
Dnlz	5.74679	5.77507	13.9159	12.0725	15.8549	13.8728	18.7515	18.4936
Dnm1	3.82163	3.8603	1.62003	1.60314	0.628607	1.28856	2.8858	3.34372
Dpp4	0.255654	0.345682	2.65745	3.67464	1.09866	1.89695	20.4566	20.1154
Dpysl5	12.5187	11.7678	3.47962	3.86983	3.26637	3.40681	0.100001	0.123035
Dsc2	12.8843	12.4089	4.80339	6.38365	3.82756	4.1953	0.74261	0.456809
Dsg2	38.5203	35.957	13.5196	19.3593	12.3889	13.6056	4.75602	5.52959
Dtna	2.29717	2.39584	0.6874	0.512009	0.585961	0.514296	1.97074	2.1524
Dtx3l	2.71449	2.84565	7.14164	15.7645	6.76638	8.55245	2.52919	2.59059
Dusp1	136.432	111.004	9.27176	7.19565	14.3842	11.8561	27.3696	35.2547
Dusp2	11.1608	10.1077	3.21377	2.17726	5.34785	3.98534	0.669819	0.308818
Dusp4	28.1066	31.5635	7.83204	8.92004	12.2701	10.0786	0.859599	0.462536
Dusp5	55.5919	51.3374	12.7832	19.4052	23.9923	19.9085	0	0.453665
Dusp8	3.62718	3.70039	0.716529	0.920004	1.0638	0.817267	0.782592	0.834457

Dusp9	23.9573	19.9037	8.83736	6.77124	13.4789	11.5067	2.30061	2.18125
Dynll1	78.9704	77.9477	190.727	124.73	210.322	184.753	79.9158	64.9259
Ebp	16.44	16.916	39.8143	31.0325	40.7083	37.2618	108.816	88.0587
Echs1	32.2614	36.4215	79.0454	76.1495	76.3902	76.3307	112.146	123.876
Ecm1	15.2011	15.1051	76.9197	40.6489	84.9334	78.0182	121.36	138.364
Ecscr	5.86369	4.66341	17.6712	17.2839	26.0918	23.2889	13.3959	11.3959
Eda2r	0.137014	0.266063	1.55836	1.60955	2.04924	1.64446	0.333797	0.19569
Efemp1	0.240423	0.329105	4.71577	8.47866	3.30592	5.13029	24.042	35.0789
Efhd1	0.499047	0.469699	3.01017	2.78619	0.928217	1.74896	124.432	163.397
Efna3	3.48509	2.95356	0.77237	0.757528	0.624645	0.951482	0.689382	1.27088
Egfr	38.3818	33.7993	11.9363	16.3275	15.2779	14.6232	8.23087	7.56058
Egln3	3.61098	3.09151	19.7333	11.6307	5.96748	10.5353	7.16145	8.03476
Egr1	207.864	194.971	28.5391	40.1061	37.0348	36.5371	6.18444	4.67751
Ehf	14.335	15.8837	46.3683	65.578	37.9766	45.1258	12.7687	24.448
Eid2	3.47065	3.95586	10.6044	5.95464	10.4706	9.70092	7.15614	9.86171
Eif4ebp1	312.276	322.634	170.288	115.213	141.762	143.409	34.9682	47.9148
Elf3	19.3996	18.6563	89.6808	85.8374	60.3434	79.2329	19.7891	30.8522
Elmod1	1.40622	1.02386	0.0740583	0.0778343	0.0829989	0.039076	0.0995974	0.306253
Elovl4	4.26749	4.34738	0.234367	0.197056	0.455323	0.461634	0	0.155165
Emb	28.7271	27.4835	66.5015	100.898	64.6189	71.3996	19.4847	19.5861
Eml5	2.33843	2.42363	0.582037	0.609944	0.635104	0.554751	2.7462	1.67091
Emp1	252.293	236.252	59.5837	53.4255	82.721	66.4027	18.7287	22.3346
Eno1b	65.6508	144.842	515.801	106.109	362.688	367.995	426.772	445.349
Eno2	2.55079	3.63129	0.787689	0.446655	0.410599	0.637918	1.87236	0.787853
Enpp2	0.338233	0.40567	2.90232	4.49819	1.98488	2.45939	50.1195	77.3211
Entpd3	20.3979	17.8584	6.31363	5.10085	8.9847	7.55974	0.369495	0.0454536
Epb4.1l3	41.4954	41.4782	18.6396	22.5318	18.7715	18.7348	11.7047	5.77524
Epb4.1l4b	0.524742	0.479989	2.93339	5.75945	3.01682	3.67361	26.1091	32.6492
Epha2	109.289	99.7447	27.7955	29.6214	46.9383	38.1051	2.64335	3.33122

Ephx1	4.64021	4.65031	31.9077	34.3961	28.5016	30.5555	55.1455	73.5844
Ereg	0	0	0.885578	0.820986	2.39274	1.65311	0	0.112856
Esd	130.9	116.409	318.1	222.867	413.84	341.705	324.953	265.476
Eva1a	1.14892	0.744949	13.2816	11.6891	10.0678	10.41	18.0574	17.8183
Eva1c	1.32145	0.974652	4.66227	3.43758	6.64237	4.12893	12.1744	14.6947
Eya2	7.90438	6.4326	21.2037	23.8071	16.9752	19.231	0.803378	1.13004
F3	98.6345	95.8013	29.4784	34.5952	39.8176	34.8566	8.52841	13.7204
Faah	1.22916	1.314	5.15084	6.14845	4.44464	5.3826	29.0704	22.0141
Fabp3	1.57214	1.89486	7.09566	7.92381	3.80252	4.11094	207.193	338.938
Fads3	51.3605	52.5262	16.166	16.6484	22.3079	20.4481	70.4358	55.0675
Fam102b	11.6022	12.4643	5.0926	4.54883	5.38109	4.92643	3.33231	3.85723
Fam111a	0.0269068	0	9.98085	11.8813	10.2702	10.2334	2.08183	2.56087
Fam129a	4.48579	4.95203	14.075	27.8584	11.8668	15.9348	2.57409	4.43339
Fam131a	4.34722	4.03599	2.38302	1.29962	1.22696	1.49536	1.84681	2.12934
Fam131c	9.84213	8.84843	4.17047	4.65524	3.53293	3.10786	2.63136	3.92791
Fam132a	2.30633	2.27173	14.74	7.79854	11.8415	10.7487	41.2852	32.622
Fam13a	0.0494008	0.0743537	0.862714	1.92826	0.428469	0.875666	24.5312	19.5771
Fam162a	95.1748	102.853	495.149	211.491	216.909	278.867	202.125	205.156
Fam171a1	11.3216	11.1612	22.9783	32.3663	19.7284	22.0456	9.39436	11.4448
Fam187b	1.24745	1.17467	3.54225	3.13375	4.96585	2.73683	8.79046	8.52898
Fam189b	0.394976	0.39815	3.66537	5.19326	4.02087	4.29562	2.43454	2.47835
Fam198b	6.60926	5.29709	0.773488	0.462885	1.07815	0.977744	0.488353	0.808878
Fam20c	2.56243	2.49212	21.2183	30.8226	10.6689	17.9723	28.9561	39.0955
Fam43a	0.252512	0.316763	1.84583	3.96473	2.71215	3.14254	8.76616	17.4584
Fam65b	5.52819	5.01253	1.14797	0.663156	1.1425	1.05305	0.687874	0.297463
Fam71f1	2.3542	2.10951	0.158877	0.0801587	0.464376	0.302814	0	0
Fam71f2	2.31517	1.22398	0.675232	0.227057	0.201617	0.19014	0.240979	0.148072
Fam96a	24.0643	28.3774	64.5689	45.7793	58.6407	54.3212	48.353	52.5497
Fat1	25.9273	22.9378	9.2935	14.4365	10.7871	11.188	4.02698	5.99823

Fcho1	7.39271	7.20915	3.45383	2.43164	2.47456	2.54318	1.07604	1.09756
Fdx1l	21.8103	24.9833	57.2912	29.7745	65.4941	51.8134	75.6265	58.0933
Fes	0.912059	1.16885	3.5823	4.401	2.84553	3.63341	5.42684	4.60349
Fetub	0.697174	0.601582	4.1483	4.38801	3.72812	4.55715	6.84614	2.54414
Fez2	68.7127	61.323	27.7897	18.0813	34.4646	29.0245	29.4788	32.8421
Ffar4	0.233703	0.125729	8.87472	11.4091	7.24781	8.39357	0.594735	0.121845
Fgd3	23.3839	21.0482	5.13423	7.08742	7.48967	6.75988	2.14483	3.90677
Fgf1	0.326879	0.276754	6.56413	6.27878	6.37261	6.04901	86.5344	106.257
Fgf18	0.716077	0.589828	2.94025	4.83276	6.47613	5.23128	0.531674	0.32682
Fgf21	1.63217	2.43053	0.233658	0.196412	0.0653773	0.0617042	0	0
Fgf7	0.130334	0.199277	1.62155	1.39369	3.93758	2.66182	0.387724	0.476894
Fhdc1	9.49659	8.65199	2.37506	3.22549	2.95181	2.78592	1.05274	0.854888
Fhl1	44.6865	43.4669	7.77483	4.48772	7.63863	6.72143	44.323	29.451
Filip1l	22.7605	23.9499	7.9497	8.94499	10.4012	9.11305	7.00145	10.8441
Fjx1	2.70951	2.97448	0.319675	0.167986	0.626937	0.421698	1.7192	2.2466
Fkbp5	16.3663	15.4685	30.726	40.4348	31.6973	32.3682	5.42031	5.8246
Flnc	36.4915	29.958	6.94495	6.38203	8.15137	7.33168	0.053928	0.0829482
Fmn1	4.60625	3.72421	1.08161	1.82929	1.052	1.22743	3.86374	4.14504
Fn1	158.382	168.389	32.0826	39.1276	37.1207	36.227	9.34217	11.3051
Fndc3c1	3.25535	2.62472	0.0484873	0.0652314	0.0761001	0.0818502	0	0
Fos	60.5156	50.307	2.50931	3.58854	4.28335	3.81163	4.6635	1.86001
Fosb	7.19701	6.26035	0.249789	0.462059	0.700005	0.448047	0.269042	0.165488
Fosl1	40.7847	40.1526	9.86405	7.79948	27.2659	18.7478	3.12E-52	3.56E-31
Foxa2	0.386364	0.343398	4.66124	3.3545	4.41746	4.13572	0	0
Foxa3	5.23716	5.01078	1.54525	1.13679	1.2986	1.27413	0.259497	0.0797783
Foxn1	2.00328	2.11977	0.509895	0.587964	0.686167	0.568772	0	0
Foxn3	6.19553	6.47373	0.923446	2.40691	1.05563	1.18888	1.39094	1.4664
Frmd6	21.3184	20.8895	5.87279	6.05876	7.8083	6.70366	2.10584	2.42475
Frs3	4.19214	3.84714	1.85978	1.17756	1.31214	1.55086	16.9074	10.6239

Fsd1	9.43743	10.2212	1.45955	1.75644	1.50694	1.8729	0.30733	0.0944705
Fst	29.1633	25.7958	4.80767	3.25475	8.5596	6.45746	7.0504	17.2046
Fth1	748.374	672.607	1355.85	1754.95	1573.44	1563.52	6839.91	6204.69
Ftl1	1417.86	1207.11	2696.98	2154.67	3246.22	2903.92	5582.4	3497.92
Fuca1	14.12	14.0034	33.3034	26.5671	31.2672	29.0582	101.109	98.751
Fuom	0.633564	0.77499	18.2949	19.0521	16.4036	18.3052	21.484	19.6525
Fut2	0	0	1.19829	2.52986	0.58036	1.04669	0.0870598	0
Fuz	3.22579	2.86478	7.91708	7.11088	6.09372	7.5625	32.2608	25.272
Fxyd3	1.16279	1.97626	13.3603	8.82995	11.7328	9.33556	6.15512	1.25735
Fzd8	0.166789	0.229336	1.10639	1.76511	1.41503	1.37696	4.88736	6.66969
Gabarap	144.161	161.565	372.864	274.042	324.326	320.836	598.656	497.361
Gabrp	4.03559	2.94613	181.541	226.429	144.804	162.53	0.0779852	0
Gadd45a	59.5078	70.8234	26.607	19.4928	19.46	21.2665	31.3895	40.5704
Gamt	0.792837	0.995927	5.51245	4.1408	4.26613	4.08844	50.4169	32.6966
Gars	225.357	230.546	101.61	87.7032	111.98	106.267	42.9314	55.2194
Gas1	0.0275256	0.0776902	0.791288	0.870032	1.26215	1.05976	10.0748	15.9194
Gas2	2.88175	3.82395	11.4226	9.35302	12.6968	11.8616	30.6982	21.3778
Gas6	4.42466	4.13161	1.48852	1.07945	1.45969	1.47263	224.403	220.239
Gata6	2.19654	2.24403	0.670466	0.363294	0.701401	0.4402	0.721823	0.789262
Gatad2b	14.0278	13.5782	3.40632	9.94704	4.19531	5.24197	1.25716	1.54589
Gatm	17.1885	16.1917	4.08304	3.29423	4.57564	4.67893	199.931	87.4189
Gatsl2	7.54987	6.90089	2.29265	4.16158	2.45988	2.6784	15.958	7.50108
Gbp2	0.8564	1.09124	9.0974	16.4925	6.03753	8.41351	7.21708	10.247
Gbp3	0.318467	0.267139	7.07499	13.1575	6.72079	7.6596	35.0347	47.135
Gbp7	0.629553	0.472304	2.65026	5.67556	2.17272	3.04944	5.35628	5.62927
Gc	1.83858	3.13353	76.6918	53.4738	42.3304	53.1437	22.4471	13.8913
Gclc	10.146	9.65108	38.6158	63.0998	49.0052	47.7361	172.464	172.583
Gclm	35.8297	33.2948	60.7806	81.8192	75.0709	72.4113	105.951	111.714
Gdf15	214.628	208.255	84.6412	61.6491	133.728	114.786	6.89566	9.45061

Gdnf	1.59857	1.27429	0.0944331	0.147458	0.257068	0.241985	0.0726378	0.0893564
Gemin7	37.9359	32.9596	80.1544	49.0438	90.5113	77.2425	89.897	85.0308
Gfra4	0.13001	0.244807	3.92127	2.31687	0.9274	2.01435	2.70246	2.00511
Ggt1	0	0.0829002	2.09556	5.34707	1.22865	2.88038	838.401	665.414
Gimap9	0.302961	0.38031	2.31166	2.06879	2.12972	2.28311	10.9933	8.35363
Gjb1	0.0923546	0.173883	1.73854	2.09212	0.763681	1.79936	35.2908	29.2228
Gjb2	0.492742	0.326277	8.05426	11.1167	10.3381	9.45845	44.8335	47.1967
Glipr2	32.8444	32.1761	15.2054	12.0043	15.178	14.1163	4.19684	3.06433
Glod5	0.163457	0.0770759	2.45455	0.38202	1.42286	2.01823	138.023	101.86
Glrp1	2.53358	4.16736	0.479886	0.233586	0.282998	0.319893	0	0
Gltscr1l	4.9242	4.58976	1.41816	2.65646	1.4798	1.83683	4.2767	4.30751
Gm10052	997.604	940.979	421.928	534.834	439.232	448.108	24.2766	54.0801
Gm1045	17.4627	26.2992	4.40636	3.3787	7.00354	5.63705	0	0.505051
Gm10451	0	0	1.75907	3.32693	2.39026	2.5884	2.68174	2.88205
Gm11538	0.482575	1.38412	3.78887	1.73172	3.58996	3.11331	0	0
Gm12238	0	0	0	0	38.8447	0	0	0
Gm12942	4.80788	3.77324	10.565	9.62823	7.85117	9.47837	16.7617	16.4473
Gm13363	11.4461	12.2354	0.000516945	6.9341	3.19721	3.48323	2.26E-06	2.59E-06
Gm14446	0.458608	0.356508	1.01878	3.21201	0.992516	1.46626	1.54158	2.18757
Gm16617	1.51022	1.77992	0.0839469	0.423332	0.187743	0.177461	0.446203	0
Gm16973	0.751274	0.910257	2.87608	2.26128	2.86842	2.89858	6.46202	4.55897
Gm17296	3.56488	3.34545	0.902077	1.66405	0.980343	1.07797	0.744028	0.549242
Gm4532	0.377554	0.284787	2.26657	1.34055	2.53453	2.0408	2.67722	1.64301
Gm5424	1.69014	1.49846	17.8698	15.1868	13.1584	13.9616	444.328	309.595
Gm5617	1.50744	1.42198	5.62561	4.23013	4.1917	4.27865	70.107	50.0653
Gm6548	10.4033	10.2214	22.8043	22.6221	22.9025	22.0816	10.504	10.6846
Gm8439	0.49801	0.470961	2.7904	3.75016	2.28069	3.15407	0	0
Gnai1	0.768368	0.748257	2.78708	2.782	3.17405	2.8937	17.8911	18.7512
Golm1	24.8242	25.5767	55.377	68.4513	56.143	57.6175	6.94602	8.98462

Got1	45.1277	42.9977	18.7241	18.1752	23.553	21.1751	49.6053	77.5169
Gpc6	1.59047	1.68804	4.54724	5.45068	2.89794	3.7792	1.54826	2.06373
Gpd1	0.748158	0.762591	17.5984	29.2889	18.5647	19.8802	120.01	82.5909
Gpnmb	1.23527	2.19736	0.186203	0.219188	0.236556	0.301248	0.133705	0.0822426
Gpr123	1.43822	1.77879	6.21086	9.94956	4.30083	5.85919	0.211465	0.487815
Gpr124	20.6947	17.5529	2.51669	2.26475	2.61388	2.48642	8.11997	5.1069
Gprc5a	309.229	272.616	100.832	80.1861	131.204	115.088	7.6539	15.5305
Gpt2	29.7553	31.7518	5.45604	5.51608	4.17056	4.79971	25.1317	19.2466
Gpx1	176.687	150.747	470.322	309.454	590.509	503.497	1174.42	731.155
Gpx2	0.1823	0.0858521	2.4761	2.37885	2.9417	3.41556	0	0
Gpx3	2.81822	3.22353	18.1866	17.2926	8.60721	14.5232	22982.6	14189.8
Grasp	18.1146	16.7146	7.43984	8.18928	8.03467	8.29136	5.9178	5.17496
Grb10	0.221656	0.451736	2.31207	7.39654	3.50474	3.79686	8.7638	5.00118
Grcc10	63.6907	70.1785	257.619	161.296	207.576	217.082	381.855	348.929
Grem1	0.138899	0.261488	0.891844	0.723946	2.85948	1.68808	0	0
Gsap	0.831935	0.975619	2.93533	3.63326	2.24535	2.79141	1.89962	1.12673
Gsdma	3.12561	3.21095	0.705419	0.607939	1.34403	1.06076	0	0
Gsr	104.202	94.9632	232.945	198.676	237.062	221.501	20.7432	28.1368
Gsta1	0	0	0.394595	0.497523	2.7227	1.73706	3.15654	1.50755
Gsta2	0	0	0.663484	1.28271	1.78169	1.75247	853.708	525.081
Gsta3	1.1762	0.704744	13.7848	13.9437	21.7847	18.1019	38.405	27.2265
Gsta4	5.60648	5.03726	50.1617	35.8726	75.9955	59.6165	488.452	283.01
Gstm2	20.4192	17.3888	76.0435	69.8346	51.9676	58.583	311.778	346.221
Gstm4	0.992345	1.0702	3.04141	4.09665	3.59965	3.6898	9.74863	14.0472
Gstt1	0.707392	0.488629	7.37383	10.9465	4.50778	6.5728	346.684	277.896
Gstt2	0.476396	0.384449	8.44285	10.1715	8.10507	8.59542	180.02	124.3
Gstt3	1.55699	1.9388	12.3265	15.5787	8.31619	9.68088	44.5682	47.0117
Gtf3c6	24.1017	26.328	61.1415	39.3326	67.9038	59.3363	59.886	63.5901
H1f0	303.216	347.996	188.973	140.809	111.775	138.354	120.053	165.312

H1fx	6.96642	8.54729	22.4109	15.1655	12.9141	16.7988	3.26105	2.00341
H2-Q5	0.444568	0.190296	1.38853	2.78642	1.15353	1.56083	1.91831	1.91543
H2-Q7	0.94918	0.867978	5.46642	8.93547	3.24988	5.11139	17.4085	17.189
H2-Q8	0.000370546	0.000339647	2.04729	5.03143	1.50888	0.000409078	5.18144	0.00133302
H2-T23	10.1122	10.8621	43.0341	39.2954	30.1715	35.4412	121.962	125.746
H2afx	28.4026	30.8801	85.9375	76.4049	84.0625	80.8913	14.6816	14.0361
H6pd	22.0861	20.5571	48.1324	83.7576	45.5975	55.6434	32.0841	35.7445
Hapln4	1.57905	1.86815	0.145469	0.16679	0.177884	0.15349	0	0.0437999
Havcr1	0.553062	0.565584	3.05738	4.09997	5.7496	5.02301	1.28527	1.05362
Hbegf	83.9454	80.8352	24.0726	22.2689	37.8411	31.297	2.74958	6.6957
Hddc3	1.77986	1.95675	6.19105	6.20047	6.04292	6.47133	11.5907	13.1562
Heg1	2.1647	2.08611	0.478479	0.607585	0.678714	0.700565	1.57842	1.5212
Herc3	5.84311	5.63928	2.23159	1.79336	2.28034	2.47946	7.44836	6.80738
Hexa	7.65329	8.99243	24.903	24.7888	16.9201	21.7869	39.3421	42.3077
Hfe	0.97274	0.940325	3.20105	2.91144	2.54294	2.39593	9.84301	14.8879
Hgfac	11.8568	10.7808	32.9588	21.4502	18.3089	22.344	25.2162	16.8936
Hif3a	0.0180257	0.0226956	2.08755	3.71251	0.807574	1.67459	0.107775	0.0663055
Higd1a	15.0429	16.1416	55.5885	27.48	37.7199	41.4935	37.7335	37.6444
Hilpda	75.6767	73.4028	28.7231	9.35853	20.0002	21.2253	4.20588	4.38444
Hint3	3.19678	3.74962	14.3922	7.65116	9.20939	8.52316	41.4545	19.7854
Hip1	16.4162	15.2358	4.88853	5.89257	4.79839	4.97186	4.5773	4.64172
Hist1h2af	0.415209	0.392332	2.78513	1.94989	3.10712	2.12986	0	0
Hist1h2bc	2.64478	3.46319	27.4322	10.9102	28.9485	26.8385	259.691	270.766
Hist3h2a	2.6806	3.10688	9.52138	9.82866	15.0318	12.6682	37.2416	45.1984
Hkdc1	0.0983898	0.0809924	1.15571	1.25754	0.929673	1.04729	0.878493	0.6304
Hmgn2	82.0744	89.1338	262.044	233.106	239.348	246.048	44.73	39.0324
Hmox2	44.9626	44.2984	117.523	109.673	95.9295	107.97	71.1526	74.2473
Hoxb13	9.07574	8.34341	22.3251	15.0747	17.1559	19.0996	0	0
Hoxb2	12.3351	13.1643	28.2402	28.949	31.4973	30.2027	20.4417	30.2407

Hoxb6	21.1998	22.6975	77.7984	52.0329	71.603	69.2041	69.661	86.1407
Hoxb7	23.0436	22.8177	56.0041	47.0469	51.0033	54.2197	20.5158	33.6203
Hoxb8	6.48908	6.8185	18.9612	21.3944	19.7399	20.605	42.4289	52.7891
Hoxd9	6.46588	8.07334	15.7336	18.3584	14.8271	16.3833	45.1079	39.2479
Hp	0.0347548	0.0327222	0.731472	1.65064	0.517401	1.13811	0.206339	0.38044
Hpcal4	5.0166	4.17184	1.15509	1.05569	1.48633	1.01733	0.270582	0.366188
Hras	26.0487	24.6571	57.2131	40.293	73.178	64.4544	52.9537	52.6375
Hs3st1	21.7358	18.4816	50.3869	54.82	68.1719	59.324	1.76058	3.64056
Hs3st6	0.116725	0.219829	7.41583	8.77148	5.26249	6.28262	1.8471	2.27007
Hs6st2	0.0294938	0.00924601	1.40704	1.53508	1.63245	1.70847	1.58066	2.26877
Hsh2d	0.0387232	0.218782	1.41601	1.87578	0.528546	1.35926	0	0
Hspb2	12.0395	11.533	5.68018	2.86607	4.39703	3.67426	7.48932	9.27226
Hspb8	0.0496914	0.0701536	1.4024	1.52584	1.07829	1.01585	7.97462	5.35679
Htra1	51.6296	50.5801	13.7382	8.668	18.9108	14.6525	31.3991	24.1728
Hypk	138.099	140.656	308.315	198.333	331.677	292.728	354.384	307.699
I830012O16Rik	1.72614	1.6453	5.87676	11.7348	5.81665	6.56434	6.05259	16.9895
Iapp	0.07812	0.147327	1.30301	0.876107	1.16553	1.28554	0	0
Iars	62.0084	62.8642	21.8722	33.485	27.3737	26.5051	8.43695	11.5802
Icam1	5.36888	4.92225	10.5628	17.282	8.57438	11.2472	14.6391	17.2498
Ict1	41.0636	42.2767	96.3685	55.5368	102.082	89.302	103.536	106.526
Id1	19.9211	17.4062	78.67	58.7373	100.679	81.5334	45.6831	28.4472
Ier2	115.149	118.856	39.5474	29.2011	45.6097	39.6314	17.2165	14.2206
Ier3	314.791	287.652	130.585	77.8902	154.248	134.214	28.4568	22.6634
Ifi204	1.4007	1.15556	7.8109	10.2613	9.34769	9.34082	2.16832	2.03513
Ifi205	0.593917	0.692165	4.50869	4.42234	4.98064	4.62721	0.336069	0.413183
Ifi27	1.99236	2.72768	138.664	157.222	115.554	129.741	75.1901	83.1773
Ifi27l2a	0	0.132586	15.6886	16.4092	14.7922	14.6193	72.4225	87.6862
Ifi35	17.638	17.6724	60.0939	56.9437	63.8131	62.3227	26.6899	21.4122
Ifi44	0.0893123	0.0840305	18.4845	36.8407	12.7478	19.1405	11.8695	14.8179

Ifi47	0.681339	0.369978	5.16316	7.60872	3.34715	4.74576	6.07369	6.04306
Ifih1	1.75881	1.81194	4.47619	8.21372	4.49012	5.05976	2.89087	2.98489
Ifit1	0.660661	0.979061	7.48916	19.9962	9.21221	9.52226	9.5318	6.04323
Ifit3	4.17234	4.30458	14.6401	27.3368	15.2146	17.6145	35.2868	67.6179
Ifitm1	0.243214	0	12.6848	20.818	6.13344	10.6903	45.2071	92.6063
Ifitm2	172.777	167.819	454.157	363.098	377.019	372.466	378.982	356.662
Ifitm3	29.1947	33.5301	694.249	724.951	657.471	667.651	189.541	198.98
Ifne	0	0	0.924045	1.5532	0.723094	0.879216	0	0
Ifngr2	20.3485	19.6949	47.1654	49.1794	50.9073	49.9795	7.65792	10.2806
Ift22	16.5668	16.8579	42.3458	32.762	37.9247	37.1595	26.4936	25.3101
Igf2bp1	6.72895	5.8904	2.26259	2.23417	2.53072	2.13838	0.324958	0.327155
Igfbp3	1.34579	1.46896	7.00611	4.17183	14.0572	9.55098	84.6454	82.8387
Igfbp4	42.0952	38.0563	219.143	259.864	161.872	200.015	294.684	133.222
Igtp	4.18196	4.43276	17.4373	25.3899	14.5447	19.4577	14.3955	12.4723
Iigp1	0	0.0276838	1.12258	3.29372	1.13055	1.51075	6.12885	5.60026
Il10rb	11.8255	13.2095	27.3031	26.5023	28.0012	28.2226	48.6423	49.3406
Il11	6.323	8.69907	0.610635	0.317254	1.02905	0.825501	0	0.267004
Il13ra1	6.12011	7.06604	33.7123	35.4781	21.516	25.7959	15.1113	17.0194
Il17re	3.49046	3.30629	8.38366	10.9731	6.03784	7.2254	19.1015	20.5496
Il1f6	0.114807	0.108179	0.764575	0.749795	2.56667	1.27892	0	0
Il1rl1	0.31335	0.624819	1.21084	2.41144	2.05763	1.82047	0.148472	0.152225
Il1rn	3.42991	4.39795	8.66846	12.9239	12.7765	10.9733	0.108274	0
Il23a	23.1136	32.8202	7.18999	3.96576	11.8872	8.0329	0.407822	0.626613
Il24	53.098	49.8448	9.53087	6.4419	21.0734	13.8584	0	0
Il33	2.63489	2.34808	7.31585	10.2041	10.3175	8.67787	3.38528	4.16377
Ilk	119.489	108.297	42.7116	70.0404	55.2837	53.5222	75.0761	75.3691
Immp1l	13.132	14.4826	38.5036	21.869	32.7411	30.1677	38.5955	31.4811
Inhba	22.8267	20.9792	3.02635	5.53596	6.77999	4.49378	0	0.10967
Inmt	0	0.0451546	2.81763	3.03891	1.66629	2.75133	39.4995	28.8032

Insl3	0.867383	0.480374	3.91924	1.21E-07	3.73773	2.79388	10.7067	2.54541
Iqgap3	25.0893	22.9593	9.76747	13.9722	9.84171	11.5034	0.176213	0.162606
Iqsec1	10.1371	8.72632	3.96479	5.46625	3.14478	3.91325	19.1515	22.6227
Irf7	1.03923	1.53392	34.1019	66.8547	29.315	42.0795	18.702	19.0547
Irf8	10.5385	9.09273	3.07033	2.35332	1.82515	2.20172	5.93306	6.5119
Irf9	4.27715	4.61096	23.8468	34.6882	23.0999	23.975	15.0753	14.205
Irgm1	17.6624	19.6614	66.0957	94.2539	70.4785	73.0878	22.0461	23.1258
Irgm2	1.4256	1.7309	7.9609	16.0149	7.14183	8.92579	4.93199	6.20099
Irs2	3.40374	2.64336	0.175811	0.354782	0.144526	0.210231	1.01007	1.66978
Irx3	0.130978	0.264077	1.38673	2.19264	1.55402	1.98768	28.0278	34.9679
Isg15	6.78333	9.88845	157.82	191.495	176.69	173.522	19.4344	18.5284
Isg20	15.7293	14.1874	62.8984	64.3911	46.533	57.3572	16.8363	10.3878
Isoc2a	0.846956	1.0496	2.86643	3.32364	3.65172	3.28815	178.965	124.568
Isoc2b	2.14684	3.39878	10.4332	7.49324	4.08229	7.54188	34.9389	34.776
Itga2	28.4391	29.7821	6.24008	20.2671	7.89295	9.98721	0.119835	0
Itgb6	2.14379	1.89167	6.81335	8.76095	6.10941	6.77672	21.9546	25.0068
Itm2a	7.06204	6.09567	1.11245	0.70145	0.900046	1.23954	2.48778	5.60753
Itm2b	95.6055	103.936	301.159	272.962	247.874	263.196	1485.79	1505.48
Itprip	16.1789	15.1242	4.15805	4.92833	5.09619	5.08762	3.43982	4.10721
Izumo4	1.11869	1.2547	4.55115	2.83199	2.64598	3.6837	22.7227	13.762
Jag1	29.7398	36.9915	13.9508	14.3679	18.4177	16.7253	7.5642	8.2415
Jag2	9.0518	7.80475	3.86071	3.61661	3.665	3.79208	3.68438	2.38157
Jdp2	40.752	40.9105	5.27054	7.4956	5.57561	6.13043	6.8077	15.7341
Josd2	5.91727	7.02939	21.2089	20.2364	26.2106	23.1523	35.5503	26.1098
Jun	33.5514	31.8706	10.3791	11.2632	11.0811	11.3143	13.3445	10.5314
Junb	331.685	375.675	99.0555	92.077	119.22	108.89	16.5532	16.6047
Katnal1	4.40087	4.47852	1.38296	2.12347	1.34826	1.52245	4.61863	4.56117
Kbtbd11	0.0897972	0.0675682	3.80997	3.65966	0.978922	2.10041	5.59869	3.72963
Kcnab3	9.12793	8.88051	2.40007	1.31327	1.79318	2.29156	1.43441	1.00814

Kcne3	0	0.0894591	2.68758	2.2689	3.59338	2.38561	2.84739	0.512788
Kcnj16	0.0353961	0.0110927	1.31783	2.94355	1.63808	2.12012	104.841	129.309
Kctd1	4.11437	3.49828	22.1766	28.9919	21.4344	23.137	14.8416	19.63
Kctd15	0.654641	0.752886	2.474	2.79037	2.88522	3.26888	5.08392	6.38575
Keg1	0	0	2.29485	3.04635	1.15012	1.62556	964.725	792.348
Kif12	0.744934	0.793232	7.65522	5.15999	7.48488	6.56849	131.514	74.7049
Kif5c	4.89623	4.70287	0.404735	0.283599	0.499156	0.384285	0.472759	0.872548
Kif7	2.89071	2.37563	1.01317	0.417225	0.706922	0.730761	0.946804	0.342601
Kifc2	2.78255	2.74685	1.10635	0.739084	0.747432	0.991499	16.5604	11.0384
Kifc3	15.1937	14.9463	3.37477	3.58387	3.05016	3.22669	41.5456	57.0037
Kirrel	13.8321	14.8387	5.19187	7.38948	6.20161	6.10979	1.77228	1.80365
Kitl	2.53001	2.50314	5.46387	7.4898	6.80408	6.46592	5.88013	4.91733
Klc1	66.7008	61.5298	26.8316	24.8096	29.8532	28.2124	30.1674	23.3011
Klf11	7.73744	8.07302	3.42582	2.8507	2.39686	3.00385	2.54505	3.91375
Klf2	7.28684	7.97801	1.28925	0.530434	1.19859	1.04233	28.2853	20.7412
Klf3	26.1297	24.6238	8.1832	15.8122	9.14674	9.52411	6.56045	6.53824
Klf5	31.5398	29.2912	7.89998	18.9731	10.0219	11.8031	0.311874	2.87598
Klf6	110.211	108.742	23.776	23.8551	33.9743	27.1165	7.91107	10.9124
Klf7	4.51809	4.45176	0.96994	2.96813	1.26009	1.7614	0.415862	0.638951
Klhdc7a	1.05323	1.33438	6.20717	11.0043	3.47952	5.78011	20.142	18.4556
Klhl14	0.042348	0.0597563	2.89736	5.26205	2.14658	2.98803	3.99217	5.44467
Klhl5	14.6729	13.7804	5.74575	5.46276	5.31763	5.47549	13.8571	12.7156
Klra33	9.63273	6.46659	0.862634	1.58147	2.6723	1.59931	0	0
Kng1	0	0	0.501878	5.82837	0.529988	1.26604	0.627594	1.40841
Kng2	0	0	0.689307	3.68864	0.807284	0.918396	464.406	530.966
Krt15	0.131621	0.0743323	1.63174	1.56785	1.6322	1.7839	5.63183	2.01967
Krt80	20.8574	17.3568	2.33666	3.52669	3.75336	2.85272	6.19819	8.17883
L3hypdh	4.70505	5.00409	1.94201	1.3606	1.68118	1.55296	26.0647	26.0263
LOC106740	6.05579	8.05213	3.94935	1.78449	3.64772	2.76318	7.65783	4.86699

Lage3	29.6568	30.8372	82.4967	51.5665	78.4607	78.0489	37.3735	44.1174
Lamb1	99.4384	102.337	41.1589	40.0398	42.6716	42.5847	35.8127	47.4159
Lamp2	40.4995	45.603	81.7336	130.738	65.8097	81.1694	200.159	173.27
Lamtor1	53.8487	58.0941	125.64	87.4119	131.301	122.128	73.253	80.7862
Lamtor4	25.7627	25.585	77.1134	47.6962	78.3783	74.3397	187.853	148.827
Lars2	38.0616	49.4898	102.252	124.607	143.243	122.689	19.4112	20.8649
Lats2	14.8816	14.7874	3.34685	4.3192	4.62606	4.6534	11.4188	12.8221
Layn	6.21063	6.46327	2.01186	2.02035	2.47315	2.12356	1.20043	1.66054
Lbp	0.380829	0.407202	6.87326	10.4472	3.9909	6.36432	2.67747	2.59651
Lca5	6.5567	6.69908	2.02645	3.80314	1.96187	2.25553	0.895005	0.917574
Lcn2	6.47354	10.3932	827.685	960.514	482.104	675.225	6.38695	8.93304
Ldhb	80.8489	79.1812	596.617	747.85	601.632	621.192	1130.6	1352.37
Ldhd	0.417507	0.411593	3.56302	6.00987	2.80967	3.55139	59.5922	75.8328
Ldlrad3	4.07422	3.85302	0.46979	0.465908	0.796363	0.571938	7.00392	7.18232
Lef1	1.54418	1.58497	0.105736	0.053085	0.268746	0.0848206	0.681526	0.978236
Lgals3	78.174	83.2495	181.23	119.03	183.657	169.54	104.631	83.8074
Lgals3bp	0.653111	0.653019	52.8814	125.315	55.6391	68.2972	42.7236	50.1534
Lgals9	13.3289	11.9158	48.4425	90.5682	52.295	58.5349	25.1077	24.1071
Lgr4	2.77361	2.46012	6.71035	10.9208	4.79852	6.73975	32.142	26.3941
Lhfp	83.4342	78.6775	23.9361	19.5118	24.9242	23.7734	18.9553	25.3309
Lhx9	2.49479	2.48315	0.109601	0.0608382	0.158577	0.207448	0	0
Lipg	9.56304	7.9579	2.15409	2.51264	2.624	3.23187	0.201168	0.659941
Lmbrd2	8.99803	8.31903	2.3032	5.54613	2.12491	3.20739	2.36121	1.55597
Lmo7	14.1922	11.7031	3.63241	6.24884	4.91415	4.81621	18.9665	25.2476
Loxl4	12.9421	11.2133	1.31669	2.7026	2.11777	2.14684	3.31668	3.51395
Lpar6	3.05137	2.95467	9.98762	10.7566	9.9615	9.54604	8.65374	6.03559
Lpcat1	34.6682	30.9737	13.5389	16.1738	15.0608	15.7875	1.52074	1.97354
Lpcat2	11.5922	10.3528	5.08993	3.31416	5.87755	4.39454	1.01436	0.623842
Lrch2	3.57069	4.18227	0.562037	0.318709	0.395585	0.455112	0.0703828	0.129875

Lrfn1	2.80417	2.64626	0.883105	0.718897	0.690714	0.682195	0.904172	0.679615
Lrg1	0.0690379	0.0324999	2.17948	3.40739	0.513884	1.61479	3.27911	1.25957
Lrp1	2.87584	2.88926	0.534532	0.426352	0.339077	0.383427	5.04232	4.80582
Lrp12	10.0329	10.8946	3.01818	3.899	3.90187	3.46509	1.05887	2.14557
Lrrc26	0.118194	0.0371	1.39711	0.734068	0.635551	1.47526	2.10402	1.86751
Lrrc8b	20.7985	17.7885	4.10038	9.47732	5.23867	6.48139	1.18039	1.11686
Lrrc8c	40.9573	38.6655	10.3228	11.5867	15.1032	11.8767	2.09781	1.85113
Lrrk2	4.86978	4.88904	0.686482	0.680473	1.00749	0.818402	7.16262	8.18392
Lsm7	71.0007	64.2117	201.458	95.2844	205.415	184.643	56.0266	57.6795
Lsp1	4.53115	4.35944	1.392	0.486854	0.746981	1.16964	13.3019	10.762
Ltb	1.32568	1.12371	10.8758	11.8608	12.1761	11.4857	1.04826	0.322017
Ltbp2	3.83069	4.81742	1.049	0.839137	1.17165	0.994288	0.0754115	0.115985
Ltf	0	0.0149109	1.80512	2.53447	2.18024	2.31172	0.471468	0.405935
Lurap1l	5.09718	4.84796	13.4704	13.7838	17.7716	16.0274	9.19807	9.84687
Ly6c1	45.8723	44.2489	7.16272	5.70868	6.68072	5.4338	152.409	168.953
Ly6g6d	0.472167	0.356389	2.73417	1.94423	1.52761	1.0009	6.12486	11.269
Ly75	9.04632	9.505	2.78952	4.9256	2.79439	3.31328	0.193965	0.0298305
Ly96	2.66823	3.10486	8.66369	7.81347	10.8614	8.93886	14.5437	12.693
Lypd6b	0.732583	0.598659	4.43581	4.41087	3.68033	3.62353	4.24248	5.64068
Lyrm2	9.68159	10.8723	27.0657	18.754	23.9351	20.2587	37.412	37.6359
Lysmd2	1.90401	1.79297	6.98944	5.46929	8.90933	8.40162	1.64758	2.60332
Mab21l3	1.50327	1.36897	7.61797	12.9783	4.32174	7.12622	1.21699	0.704519
Mafb	3.11395	2.69139	0.643749	0.423603	0.894127	0.679258	7.83589	6.07089
Maff	54.9889	60.9426	25.9008	20.5882	28.0254	24.9703	1.33888	2.28652
Mageh1	0.432247	0.594769	3.46169	3.59137	3.25848	2.41055	5.72571	8.37311
Mal2	29.9944	32.0931	62.8091	73.9188	54.2018	58.8112	33.7951	49.5843
Mansc1	1.25676	1.18259	4.53839	5.74087	3.59682	4.14226	7.80526	9.19397
Maoa	10.9116	10.7063	3.65547	2.70363	5.91902	4.42823	4.0718	4.18691
Map1b	10.7089	10.0355	2.93723	4.62238	4.54958	4.05279	0.747526	1.21371

Map3k5	1.71549	1.87769	7.22116	10.6365	6.83012	7.11845	10.7276	11.2847
Mapk15	0	0	1.43619	1.33686	1.55171	1.40757	23.8361	20.8394
Mapk8ip3	14.4565	12.4194	5.59037	4.39478	4.78643	4.84379	26.9274	18.4488
Matk	0.219326	0.33932	2.4532	1.65468	1.48011	2.3058	1.30253	0.622943
Mcam	19.1355	20.7228	7.63275	4.31553	6.55062	5.32875	8.82077	6.57571
Mcee	8.96658	13.381	25.4898	20.0387	23.3628	23.2783	169.963	159.979
Mecom	2.3624	2.05133	4.80984	7.76851	4.9772	5.3424	11.9594	13.8376
Med9os	0.229285	0.324722	1.79055	1.50453	1.28526	1.48866	6.06961	5.78646
Mef2b	1.10864	1.54893	0.158474	0.0999075	0.133092	0.0836463	0	0.130416
Mef2d	15.6732	14.6975	4.27478	7.96679	5.58542	5.59224	7.31042	7.0366
Mest	17.1979	14.8669	4.36217	2.39648	7.19353	5.3092	2.26323	1.15907
Mettl7b	9.58512	7.86117	5.18215	2.12424	3.64482	3.663	423.398	353.059
Mfap3l	17.6506	15.4751	5.18932	7.52147	7.20406	6.24968	11.0932	15.3692
Mgat3	4.63055	3.90404	1.97298	1.39789	1.61675	1.29968	17.483	16.6632
Mgmt	0	0.0594691	2.94305	1.70807	4.15488	3.77605	15.6828	13.2969
Mgst1	6.93311	7.97779	68.9956	69.7451	48.6973	59.2359	92.8118	69.7144
Mgst2	0.490367	0.277631	1.42025	0.734468	2.92947	1.73322	0.578027	0.354456
Mgst3	27.9874	27.0665	112.905	86.7104	109.607	103.9	563.136	563.38
Mical1	0.55239	0.386492	3.83183	3.68882	3.51667	3.98516	3.36113	3.33638
Mien1	54.3382	49.4761	166.322	108.446	134.097	138.041	239.349	238.372
Mif	685.142	588.585	1959.09	1124.98	1414.58	1543.52	1159.67	834.996
Minos1	16.5671	16.0721	35.9903	23.0376	39.3933	34.4958	93.9329	78.8772
Misp	1.24637	1.20679	18.2415	21.9893	18.3827	20.8319	21.0602	37.4654
Mitd1	14.0804	13.8762	33.2138	33.0248	33.5069	32.498	10.9885	7.17448
Mks1	4.21643	4.14237	1.47931	1.91749	1.08217	1.56116	6.62904	5.57118
Mllt4	57.3494	53.1704	20.6577	25.6511	20.0214	20.5757	17.7223	15.1859
Mmp11	14.6847	13.8739	7.79662	6.2813	5.35409	6.18837	4.08656	4.59502
Mmp15	0.465951	0.522252	5.96099	8.96475	5.1346	5.80375	2.36113	1.99706
Mmp7	0.635926	0.217899	173.065	205.292	78.9129	130.366	0	0

Mmp9	8.41062	9.99318	1.79984	1.76554	2.52171	2.12074	0.161473	0.198628
Mnda	1.92879	2.17863	6.13106	8.97563	7.21012	6.40749	1.63725	1.00649
Mndal	0.0234701	0.0883551	0.960978	1.70324	1.36772	1.56255	12.2781	9.60783
Mocs3	1.83516	1.67656	3.97327	4.49582	5.38831	5.20318	4.81109	3.84495
Morn2	6.36758	7.38577	16.4692	16.2552	19.2651	14.6794	96.4923	89.7278
Moxd1	10.0308	9.84527	2.36668	2.06795	2.18455	2.36673	0	0.0512072
Mpc2	13.8258	13.5928	37.0107	22.7319	35.6863	30.9593	333.762	315.247
Mppe1	1.51124	1.1487	4.23205	4.68481	4.66894	4.0473	3.92987	4.59889
Mras	6.42408	6.29132	2.40194	2.08823	2.61412	2.12831	7.28512	8.15117
Mrpl52	114.137	129.285	317.019	147.378	325.738	293.353	373.547	257.227
Mrpl54	75.8822	73.0927	206.393	122.701	209.986	188.221	317.694	246.81
Mrps12	39.5035	35.2955	97.0089	65.9412	104.689	97.3889	30.6374	39.0448
Mrps16	55.7546	56.6392	132.612	78.4728	122.151	116.778	159.609	115.102
Mrps6	49.0117	48.9867	119.132	80.4964	140.091	123.463	292.322	476.637
Msra	1.51779	2.10713	6.98152	6.25819	5.6821	6.02639	289.305	263.601
Mt1	49.657	86.4469	735.33	577.365	390.479	539.455	1050.54	865.754
Mt2	22.5871	47.467	305.013	360.704	210.799	272.539	276.655	200.367
Mt3	0	0	0.872931	1.25744	3.20291	2.375	14.4749	8.46876
Mthfd2	112.389	118.109	27.2875	31.3885	30.7892	28.7996	4.33949	5.17956
Mthfs	1.16481	1.74387	5.78558	2.5594	3.74654	4.18332	11.3459	10.6958
Mtmr10	13.8243	12.9351	2.48515	2.8187	4.31942	3.46129	5.49179	5.48249
Mtmr7	22.0836	20.745	9.28712	8.79884	10.4241	9.27344	0.80948	0.959223
Muc1	1.33755	1.44376	6.65846	12.3308	4.21841	6.08554	31.2348	53.4481
Muc20	0.652044	0.70764	3.53147	7.20008	2.05503	3.22769	16.3989	40.2665
Mum1l1	3.30712	3.16196	0.953981	0.97358	0.780028	1.11316	0.604527	0.818297
Mx2	0.0181238	0.102209	1.26153	2.13817	1.54839	1.5215	0.538442	0.596009
Mxd3	2.31557	3.03189	10.4618	6.23317	7.10686	8.33619	0.214862	0.396056
Mxra7	6.21874	6.12498	1.65551	0.879064	1.04124	1.16498	10.7597	11.2163
Myc	112.33	114.841	38.5337	37.0612	60.7409	52.0107	3.15518	2.54242

Myeov2	59.6341	60.281	177.765	98.1896	147.805	139.538	256.295	219.671
Myh10	47.1462	44.4388	13.7093	17.8179	15.6956	15.3229	8.12479	13.5453
Myh14	14.4425	11.7688	3.39352	3.49848	2.27259	2.99991	5.49674	5.15582
Myh9	175.406	163.081	64.2538	105.056	77.6996	82.4577	27.9124	38.0423
Myl6	482.768	479.893	1135.59	720.638	1143	1056.34	1640.98	1467.21
Myof	73.3118	74.7845	29.8316	41.3711	35.5959	34.4944	8.12746	10.1081
Myzap	5.49958	4.86645	2.15597	2.046	2.24857	2.00497	5.3942	4.16394
N6amt2	22.563	23.6969	49.0812	36.7224	59.7248	47.9632	24.016	24.5765
Naprt1	0.260879	0.0446413	2.38833	2.16246	1.64674	2.13301	39.8984	27.2178
Nbea	5.80188	5.24063	1.90075	2.88176	1.99135	2.14989	1.59276	1.89094
Nckap5l	3.7218	3.09194	0.855322	1.09897	0.969645	1.06463	1.24279	2.00685
Ncoa1	2.15567	2.51435	0.666138	0.649952	0.465032	0.523747	5.86164	6.67049
Ndn	0.991948	1.08932	7.96123	6.2573	9.67045	8.30719	3.11078	2.71752
Ndrg4	5.66175	5.02824	0.866347	1.18197	0.787799	1.12117	3.15345	6.86134
Ndufa2	112.973	101.119	340.692	159.841	307.437	277.759	796.705	544.306
Ndufa3	136.226	116.074	373.726	218.914	298.716	315.042	1435.65	1099.31
Ndufa8	55.8996	49.3039	136.9	90.571	138.959	132.334	551.034	515.419
Ndufab1	70.4118	68.0803	149.478	105.248	156.15	147.573	221.558	229.771
Ndufaf3	8.93271	8.92861	21.6785	15.6325	18.4422	19.0524	38.9146	36.3589
Ndufs3	47.0217	46.7531	116.722	74.8757	101.67	99.7591	258.115	240.502
Nedd9	2.31998	2.94621	6.38914	8.65115	5.64127	6.51555	8.0005	9.20552
Nek1	7.393	7.1137	2.98044	4.38324	1.97261	3.21423	1.83658	1.82216
Nenf	45.3259	50.6011	130.234	77.4501	112.011	118.499	132.048	140.099
Nes	94.1235	85.085	26.0052	23.6832	33.6789	29.157	10.0184	8.69378
Neurl3	6.9095	7.97963	20.216	22.04	25.0644	22.7926	6.93486	5.25373
Nfat5	13.377	13.6217	5.49353	7.21495	5.93254	5.62399	14.998	14.6724
Nfe2l2	29.9153	31.5361	59.1542	69.2774	60.9079	62.2395	37.1316	47.3555
Nfix	4.55543	5.03964	1.50127	1.64984	1.31185	1.41933	8.68896	9.94169
Nfkbiz	24.9397	27.8436	9.39806	10.8242	7.69194	9.0214	4.1004	4.07237

Nipal4	10.4317	8.82043	2.1769	1.61217	4.50842	3.81819	0	0.143207
Nmb	1.17859	0.893528	5.77097	4.41756	5.77231	4.80583	6.996	2.84806
Nmi	8.14728	10.4903	33.044	31.1389	30.2416	29.8565	14.8951	16.9126
Nppb	10.3267	10.681	1.34074	0.563462	2.24961	1.88909	0	0
Npr1	0.334691	0.393561	3.4914	5.00616	2.20315	3.01288	14.7582	15.3599
Npr2	1.35577	1.64928	0.245486	0.162954	0.188277	0.25902	21.1509	14.85
Npr3	6.37493	5.66465	0.788718	0.900014	1.16376	0.94657	2.47226	1.93048
Nqo1	7.20624	5.08767	13.6686	10.9142	29.1208	19.9524	23.51	28.6876
Nr4a1	48.7761	38.8927	4.55602	4.88282	6.63981	6.19054	4.31186	3.94511
Nradd	1.06884	1.00656	13.2133	7.3925	14.7948	13.6162	4.87773	3.44668
Nrep	1.54315	1.48847	5.98762	3.652	5.32839	5.29472	11.1999	12.9997
Nrg1	14.4729	12.3294	2.40421	4.08201	4.89108	4.23724	1.50545	1.07996
Nrk	2.5261	2.40577	0.125919	0.452922	0.125489	0.214022	0.0754232	0.0232006
Nt5c	104.075	95.2295	220.777	133.584	236.064	217.366	61.9205	49.5137
Nuak1	4.67116	4.59938	1.38343	2.08445	1.89442	1.72405	2.60164	2.95466
Nudt12	7.43984	7.18279	2.69347	4.31352	2.65901	2.81999	3.67787	5.15931
Nudt16l1	19.1312	17.8781	52.3358	35.2015	43.299	42.3472	57.7003	52.5234
Nudt19	6.23961	6.46395	22.2343	19.1484	22.5741	21.428	151.42	190.112
Nyap1	6.03047	5.58124	1.52809	1.59183	1.30359	1.65516	1.38343	1.25399
Oas1a	0.166208	0.290508	26.3812	37.6884	26.0842	29.3979	1.83492	3.2977
Oas1b	0.244944	0.230536	3.55423	4.35692	4.37734	3.98061	2.76782	1.88087
Oas1g	0.0447467	0	6.5109	8.86679	8.93197	7.6296	1.06417	0.736101
Oas2	0.0110288	0.0103752	3.15755	10.8965	2.58288	4.75938	1.96975	2.30206
Oas3	0.0626923	0.0758218	5.43412	15.5552	4.24994	6.88203	0.159994	0.0984207
Oasl1	3.56881	3.63343	14.362	22.5777	16.6367	16.7903	1.86159	2.67091
Oasl2	1.75866	2.09409	34.4236	62.2044	32.4199	38.0066	8.3406	9.00242
Ogfod1	8.34997	7.0867	2.5564	4.19597	3.38617	3.36209	2.80687	2.26645
Ogfr	17.2875	18.4415	36.1813	36.5797	37.2283	35.1724	18.4344	17.9542
Oit1	0.982497	0.426986	15.8687	16.8252	12.9409	15.0314	3.58833	2.61856

Olfm2	1.38571	1.64475	0.233487	0.0560848	0.149453	0.035212	2.32625	3.18981
Oma1	4.4257	4.59542	11.8718	10.2946	11.0049	11.7158	9.57952	13.098
Omp	0.765062	0.659979	2.35129	2.21411	3.50414	2.75454	0.505475	0.543914
Orai3	3.68552	4.54054	9.98503	11.0133	9.36034	10.1212	9.29618	10.0435
Osgin1	46.0718	46.8527	11.4662	14.1321	12.2828	12.8734	53.6171	54.7776
Otub2	4.82685	5.52813	1.97818	2.29413	1.81573	2.15943	1.65169	2.20093
Otud4	28.549	29.8547	11.744	14.009	15.5926	13.4837	6.47333	7.46469
Oxld1	5.31143	5.54876	19.2599	11.2903	13.9932	12.9916	40.5151	29.7561
Padi1	4.39199	3.80608	9.32277	11.0941	8.32951	8.58824	9.25E-07	0.0899005
Padi3	1.50468	0.820284	0.186585	0.117661	0.139421	0.131256	0	0
Padi4	27.1477	24.0374	2.79246	3.19189	2.98643	2.79272	0.196373	0.0603834
Palld	7.81055	6.81741	0.742756	0.825258	0.743369	0.97964	1.4998	1.58144
Palm3	38.3355	31.3086	6.70751	4.27542	9.13564	6.64002	16.3876	15.5746
Paox	11.8993	12.5724	35.8239	29.9005	40.2637	36.7795	7.11819	5.32743
Pard3	20.2837	19.7811	7.7166	12.5052	8.43383	8.62412	9.17848	12.2544
Parp12	4.72906	5.4267	15.7299	28.9283	15.5511	18.4006	6.58067	8.72892
Parp14	2.07017	2.34195	8.75529	18.4901	8.63383	10.7379	3.97364	5.20548
Parp9	5.49855	6.18667	16.9198	27.503	14.8324	19.0094	7.5569	5.97783
Pbdc1	16.5521	16.495	41.0293	32.1999	46.3192	39.6304	10.6724	13.7699
Pbp2	6.62001	5.48972	1.2086	0.700721	4.01364	1.71619	0	0
Pcbd1	44.7821	44.8092	130.426	80.7264	111.349	112.164	567.933	520.991
Pcbd2	10.9406	10.6376	39.4052	29.7289	31.3366	34.204	59.8903	54.0803
Pcdh1	25.9047	23.5272	7.79522	11.8506	9.99619	9.80356	8.18874	7.99521
Pcdh7	0.39624	0.280756	1.54045	1.6255	2.04843	1.8211	0.362454	0.412546
Pcdhgb7	0.0383491	0.0583302	0.514749	1.71662	0.825416	0.745228	2.75841	2.01205
Pcolce2	13.229	12.0205	4.20572	3.15551	7.84363	5.6191	2.00169	2.9888
Pctp	13.7695	12.5964	31.1997	30.6349	38.2436	34.5695	18.5811	17.3025
Pde4b	6.85272	6.07763	2.85657	3.38464	2.37829	2.36839	4.05809	3.60304
Pdgfb	82.0402	82.7349	23.4921	23.0874	30.4664	26.8993	7.52431	7.80244

Pdk4	6.46626	6.38691	1.05652	2.74577	0.984325	1.53465	5.17136	6.22525
Pdlim2	13.2413	11.8324	5.01374	4.82568	5.3571	4.43517	13.8971	15.1985
Pdrg1	11.2643	9.62618	25.6905	22.3879	24.6037	26.5852	43.9539	45.8604
Pdxk	1.81148	1.78176	16.509	21.5907	14.2038	15.7105	23.8908	22.2726
Pdzk1ip1	15.8482	14.9394	30.1794	37.571	28.8272	31.7997	1896.03	1318.55
Pepd	8.7745	10.1518	19.5823	26.6578	20.3472	21.8015	94.6618	89.3813
Pex7	6.53048	7.72724	16.7269	13.649	16.6344	15.5318	29.3973	32.257
Pgd	84.7489	67.9255	134.801	170.892	188.858	173.044	46.0457	48.134
Pglyrp1	0.561942	0.454222	8.30338	5.55285	5.58998	7.45634	0.948217	1.16365
Pgp	21.5352	21.3124	53.1814	31.5725	45.7533	46.5014	33.1821	28.1577
Phf19	4.00237	3.63648	1.80252	1.28242	1.32818	1.35659	0.543053	0.417539
Phlda1	108.96	92.6417	24.6306	25.4119	41.5525	33.7446	8.43225	10.62
Phlda3	19.7351	21.2456	59.6033	40.7027	75.2337	68.0014	11.0295	6.31974
Phldb2	37.4849	36.5731	16.1259	17.0775	17.177	15.8981	39.0054	33.2386
Phyhd1	1.40396	1.68558	6.72847	3.32218	4.12558	4.60863	48.8936	28.4812
Pigr	0.0221477	0.0104176	2.50943	5.43447	0.740979	1.98903	7.25187	4.13626
Pigyl	17.7917	20.3404	46.4861	32.8156	39.7402	39.8854	40.9218	37.9516
Pih1d1	17.476	21.2883	62.7146	51.0913	57.0847	58.1722	52.819	45.5513
Pih1d2	1.23782	1.0949	3.32482	3.52882	3.67674	3.07178	1.78099	2.59936
Pik3cb	15.0959	13.5129	4.58414	5.6484	6.24882	5.61721	5.07473	4.77822
Pim1	25.2328	23.8952	4.99237	5.54042	4.89001	4.63728	3.36996	5.83869
Pim3	89.6237	82.9855	39.1195	33.7171	36.0651	37.1361	74.2962	55.9669
Pinlyp	0.292807	0.069014	1.62726	1.77801	2.72971	2.49383	0	0
Pip5k1a	34.2329	34.871	8.06422	16.2695	10.2982	11.0651	3.17888	3.78371
Pir	1.25486	1.20806	3.69419	2.44217	4.49203	4.19963	0.677772	1.35409
Pkia	7.88871	8.58909	2.57772	2.43696	3.50761	3.40993	7.45852	6.45676
Plac8	12.8651	11.3224	200.017	206.727	164.315	186.318	42.8363	35.0485
Plau	470.012	423.473	64.1737	41.5837	104.664	78.8736	294.63	529.811
Plcg1	16.0741	14.2851	6.51317	8.5564	5.96423	6.54886	8.01859	7.13647

Plcl2	2.0154	1.95517	0.104424	0.00975582	0.0780391	0.0856939	3.24976	2.57562
Plet1	3.99183	4.46717	26.6872	68.9364	36.3018	40.3713	34.724	76.6566
Plk2	121.811	112.467	23.4699	22.3405	36.9223	30.0993	4.79334	6.17936
Pmaip1	61.242	72.1063	30.5253	24.4905	39.9059	33.8977	4.3935	2.762
Pmp22	0.0500274	0.0235428	0.747476	0.581886	1.61287	1.08118	16.0568	17.7325
Pnp	15.7227	16.1904	33.9904	29.3039	36.438	33.1558	15.2291	16.3103
Pnrc1	21.3131	25.2427	51.3365	49.3242	42.7077	44.9813	53.7494	53.6061
Podxl	31.3825	27.5667	9.44647	10.2825	12.1844	10.8129	46.6777	33.3997
Pof1b	0.219033	0.154539	1.91273	2.56508	1.75058	2.00537	0.325998	0.842203
Polr2l	14.1367	11.1825	31.7795	17.5085	37.2877	31.5445	38.9322	34.2619
Pop1	28.412	25.384	9.70625	10.954	14.2643	13.5494	5.18663	5.47559
Pou3f3	0.0985731	0.0794922	7.25576	10.9053	7.03306	8.51063	75.3402	93.4477
Ppdpf	26.1314	22.6593	65.2329	39.9486	56.9825	53.6725	131.019	90.1637
Ppm1h	0.183406	0.289567	1.37516	3.1196	1.49308	1.92746	10.6689	12.2314
Ppp1r12b	4.01313	4.07803	0.487095	1.1032	0.443832	0.514778	0.735687	0.554284
Ppp1r15a	34.2125	39.342	10.1753	11.0095	8.78577	9.26407	12.5572	15.3748
Ppp1r1b	0.736481	1.00133	10.8401	11.7792	5.34469	8.82901	118.646	211.638
Ppp1r26	0.430648	0.687185	2.05656	2.72962	1.23856	2.06212	2.56479	2.90204
Prap1	0	0	2.32617	3.14484	2.93875	3.4223	3.21539	3.28704
Prdx1	362.514	353.989	782.641	619.955	809.01	748.704	404.612	406.79
Prickle1	6.31621	6.67589	1.08116	1.03479	1.35381	1.3349	2.66397	2.85813
Prkcdbp	5.49336	6.07437	18.0114	12.1127	13.817	14.4912	38.5117	42.6189
Prkch	6.57446	5.68307	2.13023	2.35992	1.9844	2.27813	5.73404	6.10041
Prkci	64.6342	64.8417	18.669	27.4727	21.3516	21.7542	7.3414	9.37938
Prl6a1	0	0.0552613	0.846287	0.98496	1.23832	1.10043	0	0
Procr	37.3316	34.9416	6.5395	5.18451	9.07101	8.01247	4.90746	3.57512
Prr15	1.28129	1.07933	5.93814	5.02358	3.01165	3.94295	7.60792	7.6291
Prr15l	2.43228	2.3545	31.7228	29.9869	18.1044	23.5168	64.6805	76.5048
Prr5l	1.86809	1.70114	0.364635	0.211037	0.421154	0.32156	1.52899	1.38385

Prrg3	0.853169	1.20374	3.9832	6.50029	2.1844	3.63037	0.381114	0.755479
Prss22	437.237	379.055	84.0528	40.4397	82.8498	79.1001	0.420183	0.38735
Prss27	6.13605	4.28482	0.601467	0.272261	0.82885	0.244289	0	0
Prss46	1.27769	1.27385	0.0416373	0	0	0	0	0
Prss52	1.96542	0.993401	0.106326	0.13407	0.357062	0.168449	0	0
Psmb10	19.0122	19.65	44.8445	32.3389	40.6363	42.2674	33.2803	34.1316
Psmb8	1.39669	2.15546	23.7313	37.5875	16.7051	24.6951	29.6997	24.7583
Psmb9	0.70838	0.364172	11.0144	12.9853	5.44081	7.70863	15.241	9.59099
Psmd10	17.8301	20.3513	44.3356	36.7754	41.7845	42.9442	11.9616	17.9479
Psme1	102.533	118.554	252.268	218.25	208.922	226.97	160.089	156.816
Psme2	46.8913	56.3287	178.708	128.475	147.74	145.306	89.2689	77.0622
Psme2b	3.41151	3.75597	0.203965	0.547039	0.386952	0.161559	0.0117726	0.167513
Psmg4	21.3782	21.8595	69.3884	34.6204	60.3621	53.7449	89.7264	37.6576
Psors1c2	12.3921	9.27372	6.00248	2.21091	4.98033	5.34434	1.07869	0.220779
Psph	45.8034	50.0599	21.1259	16.9827	22.7016	19.0908	12.416	10.2488
Psrc1	5.32477	5.9425	16.9082	11.1742	13.951	13.694	0.308798	0.189843
Ptbp2	16.2403	19.5995	8.80111	6.81897	8.90072	7.94718	9.33997	7.286
Ptges	0.374974	0.451973	2.87575	2.13479	1.72793	1.96819	9.62625	8.15215
Ptges2	8.94971	7.86271	17.9578	17.0998	19.6102	17.8576	14.8328	17.4867
Ptgs2	40.4811	38.6208	11.4977	18.3255	18.0191	16.0274	0.488142	0.788204
Ptpn21	17.6063	16.7819	5.6181	7.4735	6.55011	6.59557	6.38777	6.80773
Ptprs	50.6523	46.43	25.4268	22.4471	21.3438	21.9213	8.12076	4.58227
Pts	4.57977	4.80302	21.8855	17.4292	18.2279	17.1977	88.1706	86.1473
Pycr1	4.20334	4.30411	1.33033	1.86423	1.12288	1.16116	0.0883845	0.163076
Qk	51.5868	52.682	14.8702	17.0937	19.1708	16.9092	11.26	14.1137
Rab11a	49.6506	51.6518	110.573	91.8806	117.206	110.487	74.7827	65.636
Rab11fip5	35.1102	36.8052	15.2218	16.5851	15.9689	15.7915	21.5898	19.0349
Rab15	4.08069	4.26723	1.57002	1.35853	0.905504	1.30282	2.54346	3.1287
Rab17	0.504228	0.442185	6.60671	3.9385	2.91466	3.0249	34.039	28.8653

Rab19	2.62567	2.93989	8.05786	8.09594	5.23845	7.38712	4.8447	4.66029
Rab20	12.4032	11.1339	25.4282	31.1558	21.3268	27.3126	13.607	31.7877
Rab26os	13.9045	12.2403	34.1628	12.6049	30.8319	30.7828	13.3333	12.7625
Rab32	4.50265	4.19663	12.1194	19.2112	14.1973	14.2573	5.53477	7.43952
Rab3b	7.90387	7.21687	2.38841	1.03416	2.00384	1.59963	4.7737	3.315
Rab6b	5.39047	5.9605	1.73597	1.65041	1.80237	1.74829	9.88484	10.1018
Rac3	6.43792	6.91166	25.6564	19.8894	23.079	23.88	2.13575	2.46024
Ramp1	1.97058	1.85419	11.7358	7.65414	7.49308	8.70372	0.917472	0.442427
Ramp2	0.349851	0.879102	2.07117	2.50262	2.31755	2.66681	52.4758	43.884
Rap1gap	2.30998	1.84778	6.60388	6.71047	5.74272	6.21196	60.5167	77.9964
Rap1gap2	1.46835	1.30131	4.47081	8.00357	5.41672	5.43934	7.89098	9.68526
Rapgef2	5.61017	5.57732	1.73684	2.60618	1.98686	1.9664	1.86497	2.13079
Rapgef3	10.9521	9.21093	2.93737	3.93874	4.90604	4.56327	40.0808	31.4112
Rapgef5	3.23394	2.79123	0.460108	0.912614	0.674472	0.70952	1.52503	2.81421
Rasa3	20.1521	18.1614	3.98049	3.80721	5.67674	4.83758	2.82601	2.75868
Rassf10	0.0122469	0.0230431	2.03088	2.16295	1.32034	1.6143	1.82249	3.58714
Rassf3	5.98552	5.4308	11.1718	16.1329	9.71389	11.6127	17.8138	23.2432
Rassf8	10.703	8.2803	1.82036	4.05691	2.62209	3.14147	2.79251	6.29481
Rbbp9	9.89761	10.3747	19.9276	28.3816	16.2332	19.4761	34.6087	44.51
Rbm43	6.65325	7.32249	18.601	20.7802	15.4318	17.1564	5.99291	7.78559
Rbmx	6.21449	5.54873	19.2129	16.277	23.4284	21.6041	37.4199	29.8458
Rbp1	2.43882	2.18545	26.2013	18.6359	15.7311	19.6141	10.6602	8.31626
Rbpj	39.0134	40.0979	16.0414	15.2856	15.4864	14.7743	5.06002	5.08358
Rcbtb2	2.4241	2.64466	7.4753	8.27839	7.0088	7.12698	8.9037	8.47472
Rcn3	3.24563	3.08546	0.916197	0.651755	0.513109	0.558024	43.0872	47.5078
Rdh10	9.5938	13.5056	22.1592	26.5187	28.2096	26.4545	12.7817	14.2459
Rdm1	9.91033	10.4953	42.8732	29.3501	25.8037	27.8469	16.718	13.3206
Rem2	12.9955	10.8669	3.41789	1.96731	4.2419	3.96826	0.42369	0.43416
Renbp	8.59696	10.4229	2.97108	2.01043	1.8262	2.91862	61.8096	40.3198

Rfc1	44.9575	42.8373	21.252	24.3832	19.022	20.0003	5.97048	6.88642
Rftn1	9.6012	8.61104	2.89408	2.18856	4.3952	3.41537	3.68578	4.36358
Rgl1	2.45516	1.75285	4.89474	6.84575	6.96722	6.66015	32.6914	28.0559
Rgs16	34.0302	30.9766	8.74933	11.1395	18.1622	13.8283	0.336043	0.275527
Rhob	108.849	107.914	22.7848	16.4096	23.3945	20.7599	36.0755	37.2622
Rhof	11.2645	9.45293	3.81876	4.17198	4.86848	4.63362	4.67063	8.84258
Rimklb	4.24649	4.13422	0.409691	0.137791	0.288676	0.308761	0.126095	0.193908
Rin1	17.6229	17.5058	6.76647	6.6912	10.4076	8.95367	1.51364	0.782108
Ripk3	0.85679	0.71677	3.37737	3.58524	4.7215	4.03085	1.57169	0.790596
Rn45s	140.801	140.339	713.026	600.677	1094.53	813.068	348.181	354.96
Rnf122	8.15619	7.86604	3.2412	3.35329	3.05004	3.53252	0.933668	2.03209
Rnf130	26.9803	30.6119	12.3555	5.95817	10.3918	10.132	38.0238	48.9496
Rnf135	5.12989	4.89223	12.135	12.0056	12.3249	11.9559	5.69302	4.33376
Rnf24	7.28959	7.17619	2.1059	3.25598	2.20618	2.27987	67.7563	42.3571
Rnf39	11.3582	10.3315	2.33857	2.41288	2.89719	2.84294	0.38003	0.817545
Rogdi	11.227	11.6395	23.9815	30.5841	23.0463	24.4591	172.019	140.48
Rpl41	3241.51	2564.89	7559.05	3272.14	8146.18	7013.56	1473.96	1355.09
Rpp25l	19.0951	14.3615	41.8594	29.0733	38.0144	33.8166	27.8931	30.0273
Rps27l	195.731	212.536	512.233	297.611	590.141	487.919	425.482	323.028
Rps4l	2.22863	2.24962	9.71413	10.8381	11.9261	11.3807	44.0112	46.146
Rps6ka2	6.47033	6.59822	1.21176	1.27904	1.63792	1.26937	1.25052	1.02573
Rsad2	1.03685	1.12384	3.17686	8.03444	4.27335	4.75848	7.84989	9.82204
Rsrp1	16.266	22.9705	52.3016	51.4817	68.3434	60.0868	195.523	131.186
Rtp4	0.610899	0.657209	34.4302	43.2434	31.858	32.7077	9.67809	12.6421
Rundc3b	1.85988	1.59659	0.334103	0.442988	0.489744	0.271171	0.138397	0.0425635
Rusc2	10.7952	10.9159	3.82479	4.841	4.09446	3.89635	7.00219	8.45837
Rxra	4.22188	3.81375	8.14941	10.8602	8.09556	8.57713	16.374	15.1092
Rybp	14.2272	13.9847	5.33086	8.11859	6.25202	6.38115	1.67234	3.33448
S100a1	33.7789	36.918	97.8241	78.5831	78.6596	89.7554	1343.37	1137.43

S100a14	58.3343	51.1368	29.4221	10.5084	19.2672	20.5294	0.955466	0.65597
S100a3	6.73296	5.13572	1.68208	0.494811	2.16307	1.86684	0	0.548676
S100g	0	0	0.445798	2.74663	0.165811	0.629282	2796.33	1609.43
Samd9l	5.46406	6.87021	29.1445	39.3779	21.8739	26.633	57.6996	45.0419
Sars	123.805	135.363	53.2759	53.1991	50.6078	55.1507	48.7132	48.205
Scand1	23.3358	28.6095	59.8046	50.7221	68.2732	67.2828	160.988	154.285
Scarf2	8.09247	9.03325	1.02128	0.580531	0.628798	0.880312	10.9961	11.7167
Scn1b	0.69802	0.94279	3.02442	2.23191	3.80186	3.7604	15.6837	21.8296
Scp2	14.9058	15.3168	41.0308	34.3463	33.5669	35.1728	184.273	177.794
Scrn1	3.4528	2.91526	0.112399	0.118123	0.18889	0.08898	1.35804	3.71072
Sdhaf1	13.9598	15.3751	43.327	34.363	47.2832	42.2173	53.2209	46.3258
Sdpr	0.381398	0.239222	1.71809	4.55698	1.80315	2.57107	22.1934	16.752
Sec23b	30.5973	29.406	58.4255	79.9785	62.7574	62.107	35.5544	42.4011
Sec61b	122.421	107.731	293.866	166.944	309.841	269.235	150.232	127.455
Sel1l3	10.7792	10.3427	2.84101	3.94937	2.44096	2.65832	13.3508	11.9582
Selenbp1	1.75071	1.9014	51.0429	37.0981	12.9597	27.6006	213.83	171.008
Selk	38.3841	43.7419	101.824	63.4774	95.8419	86.703	180.081	137.137
Sema3a	5.34755	4.27581	0.899657	1.13771	1.0974	1.04779	0.0775799	0.167069
Sema3e	13.2063	11.6086	2.62251	2.83274	3.61128	3.10718	0.471276	0.111514
Sema4g	3.91292	3.30072	1.77571	1.42895	0.683889	0.954	34.5657	18.9522
Sema7a	7.38863	6.32156	1.85723	3.62334	3.39907	2.37307	3.04632	4.13169
Sepp1	3.46452	3.54933	45.4666	126.746	42.9907	60.1351	444.924	432.415
Sept4	3.32129	2.35705	8.70164	9.92772	6.64869	7.20005	49.7089	78.2199
Sepw1	79.408	70.3933	289.494	164.738	328.565	305.009	306.591	237.06
Serf1	29.9124	36.4622	93.8455	51.9636	80.8046	69.5598	45.0861	38.9902
Serpina3g	0	0	0.56105	1.43564	0.76532	0.926842	2.22837	0.402984
Serpinb9	11.5031	12.0336	20.5209	33.2172	27.1953	25.9244	17.9628	28.0141
Serpinb9b	12.5222	12.3962	0.564541	0.798259	1.26508	1.21879	0	0
Serpine1	108.729	90.3987	42.471	29.7979	41.8294	39.0637	0.51023	0.523016

Serpine2	2.87757	3.4614	12.2806	8.23185	17.4891	14.3507	41.939	25.629
Setd7	16.8689	15.509	6.62381	8.53867	6.23326	6.34057	4.48942	4.38183
Sfta2	2.45158	2.08377	8.21084	4.94421	7.48504	6.80731	0.720436	1.32424
Sgms2	31.5004	29.4114	10.602	20.8726	12.0568	13.4155	13.7248	11.0189
Sgtb	3.97159	3.19222	0.551242	0.956863	0.956602	0.675526	0.287009	0.294182
Sh3bgrl3	76.8191	73.5845	288.909	234.329	309.575	304.303	506.677	394.003
Sh3bp2	9.22511	9.74226	4.64457	3.86227	4.44889	4.11292	5.15642	5.26766
Sh3bp5	29.4384	25.1017	9.43722	9.54794	12.2218	9.81451	27.9056	26.2918
Sh3kbp1	5.61415	6.50378	13.4086	10.5749	16.7143	14.7246	4.3763	4.25255
Shisa5	31.7971	32.1676	56.0246	84.7943	58.3695	66.0051	83.7912	97.4181
Shmt2	110.727	113.739	49.5438	55.4454	60.1131	55.0627	108.823	70.5643
Siglecg	1.62083	0.911755	0	0	0	0	0.314412	0.322252
Sim2	0	0.0657071	6.55008	11.664	6.99605	7.68513	6.23674	17.0923
Skil	22.7105	24.3056	10.1277	9.89099	10.3539	9.26	2.72784	2.78124
Slamf9	3.98181	3.82883	0.557496	0.156221	0.156051	0.29436	12.6804	8.86063
Slc12a4	20.7159	18.7223	6.68883	6.42325	8.11928	7.41176	10.4818	12.6908
Slc16a12	0.0220219	0.0725084	2.00829	3.34652	1.30981	1.84929	18.0268	15.1206
Slc1a4	20.9773	21.7134	3.81271	5.22203	4.36802	4.38967	9.55707	10.6452
Slc22a18	0.152989	0.0576026	1.89747	0.911561	1.66998	1.25195	778.909	482.797
Slc23a2	4.03674	4.47713	1.11148	1.74089	1.59637	1.48682	4.42513	3.99923
Slc25a10	9.47739	9.08568	21.9278	22.3917	24.2248	23.533	86.1886	64.4294
Slc25a11	33.4359	36.5092	77.236	68.6622	75.9471	71.6874	92.6368	95.8359
Slc25a38	3.61563	4.38368	8.80667	7.29629	11.085	10.5474	23.3707	17.0072
Slc25a48	2.36686	1.82301	5.44598	14.3365	4.91829	7.33026	1.09547	1.57117
Slc27a3	0.856368	0.695963	3.05731	2.28087	2.46096	2.65849	5.55629	2.27779
Slc27a6	0.0839455	0.126378	2.0982	2.90381	3.30874	2.5081	0	0
Slc35a1	5.80451	5.75007	14.2452	11.9769	13.7705	12.3081	19.9812	21.3806
Slc36a4	2.79317	2.97634	0.689695	1.24564	1.08761	0.754907	0.548941	0.590646
Slc37a4	3.66295	4.84999	15.6453	11.7277	8.81832	10.7926	54.0722	34.1045

Slc38a2	107.269	117.785	36.0008	41.7674	41.3661	38.0788	20.675	30.189
Slc39a4	3.01459	2.79947	6.01163	11.6768	6.22028	7.59484	0.471804	0.217589
Slc3a2	140.954	149.979	47.5144	49.5267	58.6947	52.9349	188.232	106.147
Slc40a1	9.38076	9.3866	22.5384	29.7474	14.944	18.7025	14.6579	12.594
Slc52a3	2.45976	2.51726	0.529941	0.247029	0.360442	0.34054	11.4651	10.0987
Slc6a9	8.76197	10.8263	2.61426	3.57801	2.40933	3.1876	9.61692	12.2147
Slc7a1	27.4386	29.2557	4.07604	6.65495	4.82909	4.67252	1.93801	4.06663
Slc7a5	78.5531	79.026	16.0613	20.0416	20.2991	18.8379	2.39418	2.40974
Slc7a6	26.9173	24.6549	9.14679	12.4882	12.0541	11.6666	2.24827	2.89153
Slc9a5	5.95771	5.27795	1.79076	1.4458	1.69798	1.61671	0.816195	0.443031
Slfn2	6.20021	6.11889	16.2972	19.8533	17.9186	17.7137	12.6595	11.3743
Slfn9	1.71318	1.94438	4.26402	14.3924	3.5335	5.38851	0.0658009	0.121424
Slk	40.3432	40.8887	15.6645	20.201	17.9269	17.3747	17.706	15.2112
Slpi	0	0	1.28706	0.595027	2.01626	1.01981	0.686563	0.421605
Smagp	32.0494	33.963	72.3541	65.471	62.0264	65.1987	103.032	141.43
Smim1	3.00078	2.85372	7.57505	8.30352	5.57211	6.44636	37.9061	26.6421
Smim22	4.88991	5.11132	51.0054	21.7697	42.1808	43.5733	136.913	113.3
Smim24	0	0.287356	1.13098	2.376	1.26344	1.79444	208.691	165.355
Smim4	6.03651	6.04634	19.5169	8.69796	19.3035	13.2997	109.93	86.8961
Smim6	0.25405	0.335059	5.01632	2.98489	4.35293	4.46508	24.3891	18.4946
Smpdl3a	0.302021	0.379016	3.00844	3.93451	1.52923	1.94056	87.9592	74.6778
Smtn	86.7325	83.4718	30.6545	32.4148	38.9669	37.119	51.8467	56.0341
Smtnl2	4.15273	4.5013	15.1191	8.8042	5.97071	9.32476	18.8759	17.4429
Smurf1	35.3584	34.7921	12.7341	15.9591	15.6746	15.0169	4.88673	5.7522
Snhg1	185.001	201.903	100.593	53.643	106.861	97.8925	26.8882	13.0773
Snora16a	0	0	0	12.0109	0	0	0	0
Snora21	0	0	49.3428	0	0	0	0	0
Snora33	0	167.903	0	0	0	0	0	0
Snora34	50.2525	0	0	0	0	0	0	0

Snora52	0	10.8134	0	0	0	0	0	0
Snora64	55.898	43.9456	0	0	0	0	0	0
Snora78	0	0	0	3.73342	0	0	0	0
Snora7a	0	0	0	0	0	27.3531	0	0
Snora81	0	0	0	2.81472	3.61868	0	0	0
Snrpe	175.959	178.538	442.669	201.034	424.641	380.222	96.9645	76.897
Snx9	70.428	67.8004	23.6387	34.0793	30.3602	30.459	17.7945	24.039
Sorbs2	14.3023	12.532	3.7765	3.76539	5.47473	4.34793	4.98773	4.60819
Sorbs3	11.8075	12.7505	28.6699	28.5454	25.7247	26.219	65.4954	58.9346
Sort1	5.21555	5.39598	2.0298	2.59757	2.14456	2.02716	15.8827	24.301
Sox11	18.9935	17.4755	6.75749	5.84648	9.28812	7.9138	0.0878758	0.144173
Sox17	2.35559	2.05885	13.4003	13.0345	13.39	13.3331	8.58708	6.72273
Sp100	0.102563	0.11582	3.13172	6.907	3.28043	4.09554	10.9786	13.7265
Spa17	0.867647	1.19007	5.87693	3.68605	3.43255	4.63612	64.296	62.6115
Spaca7	1.80272	2.41832	8.32027	6.02198	9.39229	6.83892	0	0.251674
Sparc	88.6251	77.0813	17.9935	11.5227	21.1262	16.5617	85.4915	72.453
Spc25	18.9515	20.907	61.6621	42.548	48.7297	47.6112	12.2964	6.11388
Specc1	28.3749	26.1833	9.95379	15.7494	11.7522	12.0429	4.59287	6.46654
Spg20	27.3292	25.4861	10.7884	11.8277	12.3464	10.9593	6.5584	9.71214
Spint2	272.398	288.419	613.444	503.662	597.654	583.989	525.951	556.379
Spn	2.02886	1.7337	0.402459	0.381857	0.521627	0.511435	1.57693	1.3748
Spp1	786.743	868.529	3101.5	3473.41	2931.69	3061.76	2101.16	1705.21
Sprr2e	0.344193	0.649298	3.44704	1.20707	2.56868	3.03712	0	0
Sprr2g	0.767941	0.885275	7.50043	6.06416	8.2779	7.62886	0	0
Sprtn	7.18799	7.1982	2.10987	3.95717	1.92254	2.66721	2.74155	2.83513
Spry1	34.529	32.953	14.868	12.174	14.3101	13.7418	12.3349	15.8781
Spsb4	3.89567	4.31722	9.47238	10.7782	9.19288	10.7125	17.5236	13.9576
Sptbn2	4.62948	4.30671	1.07092	1.60867	0.731697	1.10033	15.6096	24.8326
Srcin1	2.54088	2.48569	0.423766	0.189296	0.155898	0.244486	7.96047	4.7437

Srd5a1	27.2183	27.0549	114.099	92.5763	114.268	107.363	4.37413	4.00519
Srd5a2	0.418964	0.169204	2.39207	2.34579	1.41258	1.75506	1.06294	1.30537
Srxn1	5.61546	5.10929	28.4386	32.5604	38.1066	34.4561	28.4567	26.3083
Ssh1	13.6062	10.1846	3.59969	4.38863	2.77389	3.40268	1.61473	1.48971
St5	4.03882	5.07459	11.9914	12.5171	9.85366	10.7564	14.378	17.3719
St6galnac2	1.11649	1.13313	10.0039	16.4145	8.35958	10.5584	122.747	84.1968
Stambpl1	20.6193	20.0845	8.28756	6.33821	7.16031	6.82377	2.14599	1.56687
Stard13	7.42692	8.22254	2.23005	2.49964	3.12376	2.76413	3.32743	4.5777
Stat1	2.27489	2.24756	14.0992	30.7962	14.2418	17.8061	7.57071	7.49996
Stat2	4.69647	4.59935	9.01738	18.1216	8.639	10.6836	10.3933	9.81954
Stbd1	11.8878	14.5927	7.2857	5.18473	4.73649	5.51134	3.60739	6.57193
Stc1	0.33068	0.278904	3.11344	1.99252	2.00641	2.31418	1.8328	1.92068
Stc2	0.125686	0.0236592	3.95064	8.02301	5.42361	5.69701	1.64347	1.28596
Stmn2	0.964499	1.21769	19.0038	11.4688	24.7635	20.4152	2.51723	1.37571
Ston1	14.8646	13.1675	3.28407	6.29027	3.32175	3.27945	2.06921	3.34065
Stra13	32.3317	34.653	74.125	50.1824	76.931	71.2231	194.934	158.8
Sulf1	0.0280228	0.0263605	0.993887	1.08966	1.03461	0.930974	0.500649	0.444853
Sulf2	0.540087	0.539754	4.1312	3.33989	5.53637	4.56798	9.78142	7.58264
Sult1d1	0.0183319	0.0172499	24.6571	42.0932	14.8386	21.934	221.359	161.203
Supt20	25.8837	26.2598	10.3671	15.8707	11.6442	11.0479	11.5988	7.46324
Sv2a	2.73991	2.49569	0.384592	0.434532	0.633203	0.570637	7.70286	5.85296
Syde2	5.41494	5.2301	1.66501	2.16041	1.97089	1.89037	2.36862	2.80218
Syne3	4.29111	3.76329	1.16069	1.0599	1.19812	1.22574	0.738434	0.907977
Syngap1	2.32611	1.76052	0.338929	0.933551	0.340631	0.64122	2.26618	0.813148
Syngr3	24.8743	23.2166	3.55726	3.05442	2.742	3.56812	0.406644	0.50005
Synpo	2.58894	2.67627	7.27735	8.1211	5.08655	6.23907	19.6597	12.2126
Syt11	5.16925	4.63602	1.69963	2.22113	1.82501	1.56636	5.18527	4.75062
Sytl1	0.139453	0.131245	2.03186	0.778462	1.90196	1.76451	5.94145	6.37157
Sytl2	2.95327	2.46583	8.85238	11.1047	7.04937	8.32041	6.13339	10.7041

Tacc1	5.1265	4.91995	1.56208	1.8053	1.83327	1.73698	10.963	14.4458
Tada1	2.16913	2.43007	7.65649	6.68714	7.37584	7.67053	6.511	6.26977
Taf10	112.675	110.034	240.394	180.146	280.238	253.725	225.386	214.109
Tal1	0.141037	0.09476	1.5305	1.63871	1.44818	1.44527	0.899726	0.885572
Tap1	1.48068	1.71034	10.2127	14.5345	8.06492	10.7766	7.15371	5.17353
Tars	117.974	120.46	51.3699	49.9697	63.9643	58.0289	9.34284	12.3987
Tbc1d16	2.95038	2.67953	0.613706	0.732433	0.584494	0.633516	9.67688	10.8562
Tbc1d9	4.78717	5.04264	1.13928	1.26621	1.63221	1.7559	1.70503	2.54667
Tbkbp1	1.58357	1.55038	0.384315	0.394944	0.319092	0.255325	5.0576	4.28908
Tceal8	3.28093	3.45236	20.9922	13.2354	21.4646	18.9639	32.784	23.019
Tdrkh	11.5713	12.2794	4.48986	2.17026	3.52441	3.1267	0.977907	1.32308
Tecpr1	10.9544	10.0746	3.44979	4.8534	4.01887	4.34163	8.80549	6.2383
Ten1	23.033	20.8593	57.4521	35.1835	52.281	53.0246	12.2507	21.757
Tesc	0.582928	0.79882	3.70418	2.91602	2.10573	2.11169	61.536	80.603
Tgfb3	0.697075	0.631952	1.8636	3.00427	1.97896	1.96639	1.05614	0.649604
Tgm2	13.469	11.3667	36.0987	60.4131	20.7848	35.2761	82.3295	69.2708
Tgtp1	0.216225	0.159847	6.11435	13.2975	4.49837	5.62307	1.1947	0.621716
Tgtp2	0.0154037	0.0289861	0.732457	1.77593	0.553646	1.04254	2.01646	1.80385
Thbd	14.0095	12.3692	3.402	2.16669	4.88067	3.95118	10.6641	7.94706
Thbs1	68.5862	54.3444	10.2998	14.4251	19.144	15.1234	10.8531	20.6395
Thbs3	7.77684	6.87122	1.64492	1.7854	3.017	2.44575	2.09288	0.89116
Tiam2	4.64885	5.93713	1.98868	1.37347	2.82976	2.19725	1.52748	1.70694
Tigit	23.986	19.4866	1.54506	1.29878	2.94621	1.81336	0	0
Timm10b	14.341	14.9534	34.3908	21.3939	34.6269	31.0524	34.5853	28.2661
Timp3	5.47192	4.93491	0.42912	0.352426	0.469878	0.494862	233.41	214.332
Tinag	2.89179	2.4472	8.5457	10.4684	7.00764	7.40859	54.708	47.7836
Tjap1	38.082	36.3831	17.4817	15.9576	19.2681	18.4451	8.12836	8.00164
Tkt	168.434	145.857	338.752	339.747	335.289	338.189	137.718	114.207
Tma7	126.586	127.698	283.625	192.482	289.67	270.719	177.918	164.825

Tmbim4	30.1717	31.4617	75.9296	59.5069	81.9919	74.0539	563.67	442.372
Tmc4	15.5518	16.8556	36.0841	43.7393	34.3983	36.5497	43.3534	49.8241
Tmco4	1.65475	2.33517	5.20256	6.97013	3.95707	4.9014	8.69493	11.1219
Tmem100	0.0767874	0.0481823	2.63469	2.57237	4.09439	3.76781	1.9776	4.30208
Tmem116	0.649884	0.611788	3.29577	2.99471	3.13371	3.23961	37.8013	37.5046
Tmem120b	6.3001	7.58787	2.5912	2.96463	2.66377	2.45303	7.46124	7.67493
Tmem121	11.3017	10.531	3.12805	2.09856	2.61924	2.83508	0	0
Tmem123	29.2394	28.4052	72.9618	108.307	61.9627	71.4958	25.061	30.9365
Tmem130	2.32098	2.13893	0.210963	0.192158	0.216736	0.0556549	0	0
Tmem144	8.28157	7.52177	2.10428	2.18026	2.38192	2.22075	2.85709	2.74077
Tmem147	45.0977	46.0326	99.992	68.1264	116.984	98.9695	152.079	158.842
Tmem158	6.36956	6.70778	2.42715	2.16207	3.23714	2.74497	0.620557	2.00289
Tmem173	13.3212	13.5179	43.1981	53.7184	36.9358	44.1306	2.85025	3.06711
Tmem176a	40.9729	48.5564	103.974	128.153	90.1707	102.118	962.869	680.285
Tmem176b	122.379	128.569	284.705	312.245	278.011	283.206	999.165	955.411
Tmem178	0.0267394	0.0503371	19.7128	18.6884	14.2917	16.3403	19.0679	13.7736
Tmem200b	0.0340283	0	3.20376	3.644	1.56191	2.42741	4.64698	4.84286
Tmem205	9.34013	9.5123	30.7975	17.0132	22.5189	21.8925	354.782	188.448
Tmem213	0	0	3.44341	11.6807	2.70566	4.45327	724.99	565.096
Tmem238	13.922	17.5279	34.4771	27.2955	51.442	41.5755	55.0289	47.3284
Tmem256	72.8085	77.5185	202.425	94.5493	152.95	158.497	356.709	214.329
Tmem260	1.0408	1.10803	2.98697	3.50117	2.48901	3.01023	4.61276	5.67529
Tmem30b	1.59748	1.70002	4.45889	5.55057	3.16424	4.15497	18.1589	35.6157
Tmem42	0.60748	0.572158	3.9897	2.6134	4.11825	4.18421	8.90552	11.0621
Tmem45b	1.77198	1.64067	6.85046	4.56962	2.59399	4.65129	60.2294	50.8148
Tmem63b	71.1625	66.7752	23.7975	30.6313	31.9758	29.8382	20.8767	18.8715
Tmem71	4.25123	4.16645	1.64857	0.850881	1.07936	1.13677	1.40545	0.594265
Tmem80	1.30974	2.02834	10.3155	9.41882	13.6494	10.3321	24.5065	26.4767
Tmprss11e	3.61159	3.56348	0.62605	0.280741	1.6218	1.23309	0	0

Tmprss2	4.34624	3.34556	9.59581	19.4042	8.7969	10.8884	49.3011	45.1847
Tmprss4	0.0972215	0.0731892	3.01136	5.02758	2.36219	2.79215	0.462583	0.0711131
Tmsb15b1	0.718096	2.55245	4.37261	3.56419	6.21231	4.78473	3.59643	2.75159
Tmsb15b2	0.109274	5.06E-07	2.69979	1.03187	2.58121	1.21065	0.000141692	1.43631
Tnfrsf1b	2.94552	3.03728	7.07051	11.994	5.4475	7.41963	0.920501	1.60439
Tnfsf10	0.0170634	0	0.76374	1.90275	0.369966	0.815725	7.67184	10.9078
Tnip3	0.328288	0.564422	7.18291	9.87998	6.98563	6.86585	0.134783	0.0829049
Tnk2	45.5227	40.8536	19.3202	19.0906	22.9284	22.0078	12.0531	9.27159
Tnks	12.0985	11.7887	2.56753	6.84452	2.81428	3.56367	1.31448	1.83142
Tnnc2	20.9788	18.8933	5.50458	2.71708	9.67157	5.81875	0	0
Tnpo2	44.681	43.0771	17.2485	21.2991	20.5947	20.6023	11.7788	8.73954
Tns1	2.60545	2.60384	8.19607	13.189	4.93167	7.48619	102.086	118.919
Toporsos	4.282	2.78958	11.4856	8.37369	11.4206	10.9222	78.9846	46.2949
Tor3a	6.30047	7.18972	13.3645	19.6006	14.2791	14.9766	6.96756	9.43768
Tor4a	6.55346	6.45953	15.1816	17.1477	13.0806	14.137	8.70987	9.56975
Tpgs1	7.02454	7.77363	21.8537	16.2845	19.181	19.1724	46.8681	44.7924
Tprn	9.40937	8.88216	17.0686	20.3129	19.5514	19.9046	19.2144	22.3902
Traf5	1.15617	0.811073	4.13981	6.17906	4.4564	5.27735	3.75004	5.38033
Trappc2l	32.9527	33.7317	78.9978	61.6711	69.5766	72.8785	57.8013	66.8496
Trex1	2.92341	3.41799	8.96429	9.24171	11.9281	11.0857	21.2444	17.6273
Trib3	32.2628	36.8507	10.5795	8.12841	4.94609	7.72239	0.644604	1.82321
Trim12a	0.933845	1.58803	6.6377	6.67409	5.19348	5.0001	13.7785	13.6392
Trim12c	0.548409	0.730879	4.71801	6.1805	3.27041	3.95842	11.3269	8.44231
Trim14	1.74606	2.05971	4.64806	6.26797	2.76305	4.74785	1.27901	1.62249
Trim21	1.21344	1.40531	2.99341	6.37887	3.00905	3.84861	4.16556	3.24457
Trim30a	0.0226343	0.0106465	2.23934	5.80642	2.48215	3.32637	2.96439	2.81798
Trim30d	0.011355	0	0.612851	1.70024	0.471064	0.82674	0.946996	0.527284
Trim34a	0.997967	1.30507	4.24654	6.90496	2.9771	3.79035	4.12584	4.70329
Trim54	3.38647	2.68008	0.0995487	0.11159	0.223021	0.140119	0	0

Trim65	0.636769	0.732228	1.84625	3.02573	2.17456	2.37743	1.93687	1.65739
Trmt1l	6.33895	6.67149	14.8157	18.2904	17.9208	16.054	19.149	16.2599
Trp53inp1	2.74333	3.85665	9.68507	6.85665	9.55358	8.76797	6.4612	5.00248
Trpv6	0	0	1.5033	3.65454	1.04391	1.7415	0.967296	1.51432
Tshz1	2.33065	2.55873	10.6845	17.6259	7.2319	10.5571	6.3175	5.81649
Tspan2	2.70994	2.31132	0.288947	0.249862	0.427557	0.280477	1.74312	1.11146
Tspan33	4.6283	5.53594	30.88	32.9835	18.5438	24.1248	57.3369	75.6742
Tspan7	26.555	24.7783	8.69913	5.63323	8.61065	7.48838	38.8521	28.9047
Tspan8	136.61	113.764	704.323	541.365	506.862	541.621	47.4485	85.0022
Ttc30b	0.934407	0.973403	3.24786	3.47552	2.3577	3.07686	5.06284	6.41027
Tub	2.64627	2.15058	0.592632	0.262268	0.48083	0.419559	0.084061	0.051714
Tubb3	5.25466	5.71249	0.668877	1.00245	1.59645	1.53486	0.156138	0.0959892
Tusc2	13.1097	14.1431	30.3274	27.4077	33.5677	31.4695	38.5427	45.8885
Twist2	1.50203	1.27955	15.7285	13.023	15.7511	15.6445	0.212298	0.130471
Uba7	0.31326	0.730396	5.33089	8.73658	4.18599	5.48208	4.70149	4.28763
Ube2l6	6.40831	5.84375	10.9255	18.7138	11.1961	12.9471	26.895	19.5911
Uchl1	129.038	128.928	29.1154	13.4463	53.8824	41.2232	10.4213	9.45236
Ucp2	9.08704	8.25816	31.9391	32.9216	29.7408	29.8025	76.1358	68.5554
Ufc1	38.3291	38.0501	98.8722	65.3369	94.7986	88.5603	120.275	95.8133
Ugt1a6a	6.57E-11	8.08E-47	1.47451	3.82999	1.50673	2.06849	1.48436	1.3617
Ugt1a6b	5.48E-07	0.00147718	1.80855	0.462364	1.50065	1.40487	348.708	211.528
Ugt1a7c	0.600013	0.710748	12.8929	23.0622	18.1232	16.1915	15.4644	5.64381
Ugt2b34	0.283205	0.146547	2.20765	2.05367	1.63206	2.06518	16.4327	7.67076
Unc5b	28.3436	26.3173	8.92611	10.4943	9.66195	9.55873	1.40502	1.57156
Upk1a	0.502444	0.473078	2.94214	3.84383	2.58252	2.93849	5.11305	4.05879
Upk3a	0.131003	0.0411269	2.66253	1.18017	0.975575	1.78932	9.84156	1.43208
Uqcr11	133.375	129.321	388.528	198.453	374.849	351.739	1808.39	1489.09
Uqcrb	150.661	160.166	412.252	250.182	409.594	359.152	1311.34	1221.18
Uqcrh	239.49	235.99	670.986	363.821	620.465	546.619	1749.06	1694.25

Urah	0.324956	0.842743	4.87948	3.49339	5.05108	3.72548	7.19601	6.77013
Usp18	2.49739	2.68637	20.0823	35.9735	26.1965	25.6335	3.33209	4.74887
Usp44	1.91387	1.906	0.601807	0.54545	0.444004	0.478574	0	0
Usp53	10.7994	11.0664	2.79979	5.41745	2.66578	3.20126	2.33458	5.12317
Vash1	2.65218	2.3228	0.553231	0.490325	0.603489	0.615202	1.12837	0.892511
Vcam1	11.5879	11.0086	46.9461	73.6301	51.6206	52.6867	4.2827	3.14252
Vegfa	57.4523	63.571	31.7522	18.1486	17.1288	20.6139	104.847	62.1129
Vim	32.9991	24.4819	60.5874	29.7009	88.0669	66.098	93.8478	67.3749
Vkorc1	16.0957	14.171	41.4464	36.359	35.8675	36.7243	79.2316	98.0366
Vtcn1	1.09722	1.29831	5.46058	6.63174	6.07862	5.78181	0.593442	0.425784
Wfs1	52.2211	43.3003	17.2793	17.0505	22.6564	19.1703	8.64677	15.4281
Wipi1	33.2167	33.1981	13.4055	11.6394	11.3029	11.3139	14.2813	14.1208
Wnk2	0.0361568	0.0509994	0.760395	2.08006	0.6175	1.05486	0.843563	0.484368
Wnk4	0.839734	0.645442	5.56907	7.24281	5.34202	5.72061	130.418	119.117
Wnt16	0.981975	0.949979	4.71048	9.36753	2.97679	5.16354	0	0.199283
Wnt4	0.947984	0.681968	5.49847	4.71651	2.86067	4.05847	4.91325	3.02216
Wnt7a	34.3792	35.5841	4.67647	3.51612	8.66891	6.24726	0.161365	0
Wscd1	0	0.0298684	1.8957	1.29873	1.96725	1.9634	6.51639	7.55053
Wwc2	31.513	30.8957	12.7508	17.8455	13.1216	13.0618	10.6282	17.435
Wwtr1	40.0957	41.3676	17.9484	20.1504	19.1578	18.5417	15.2559	17.5416
Xaf1	0.352571	0.576095	15.2282	16.6286	12.9902	14.0604	4.40018	2.55925
Xpo5	36.4138	33.2166	11.7465	20.2241	16.9903	15.6973	5.07149	5.90167
Yipf2	7.14322	8.01968	24.4233	19.0844	20.8474	20.671	34.8567	29.1202
Zbed6	8.95099	8.05537	2.24272	6.83096	1.83614	2.73924	3.30959	1.88171
Zbp1	0	0.140117	9.27997	17.0473	8.52388	9.5222	3.57713	3.43344
Zbtb32	3.40627	2.48825	0.562698	0.804244	0.630422	0.593944	1.96405	1.76478
Zbtb45	3.47767	3.54539	8.42786	8.18341	8.28906	8.91886	5.90873	6.96372
Zbtb46	1.42511	1.3447	0.142991	0.2282	0.252111	0.257175	1.49703	1.76749
Zbtb8os	19.9317	22.9846	50.9644	39.465	44.4734	44.0307	54.3839	53.3852

Zc3h18	53.199	46.9245	17.4303	29.4661	19.7711	20.6984	16.269	18.6202
Zc3h6	3.43284	3.71347	1.53651	0.869271	0.435949	0.700466	2.2998	2.29504
Zcchc18	0.445109	0.53432	1.79137	2.85423	1.60715	1.79425	0.828627	0.452986
Zfhx3	3.88819	3.31311	1.0198	1.31068	1.05986	0.979551	0.641144	0.798161
Zfp105	0.244627	0.334865	2.43611	2.52402	2.26091	2.33742	1.45446	2.03248
Zfp467	0.983475	0.846023	3.13796	3.95845	2.2	3.03772	51.0873	47.8709
Zfp518b	6.02172	5.10121	2.09869	2.23145	2.05	2.01408	0.802906	0.763406
Zfp647	4.89002	5.63467	2.09763	1.74507	2.15264	1.74772	1.18699	1.53279
Zfp771	5.81952	6.96783	17.979	19.5304	20.3242	18.7588	18.983	20.8005
Zfp9	6.92936	6.7332	1.24859	1.10031	1.0901	1.17806	0.840591	0.556831
Zfp951	1.93865	2.56763	0.758546	0.429811	0.68384	0.730833	0.354961	0.818658
Zfpm1	7.94418	8.42738	2.45133	2.65	2.27666	2.45353	3.31828	5.28799
Zfpm2	4.27311	5.03234	1.34927	1.42601	1.4391	1.12696	0.353428	0.621189
Znfx1	3.61377	3.35272	7.24168	13.0469	8.20644	8.7767	22.6699	21.7827
Znhit2	9.35157	9.26337	23.9139	17.8741	25.9909	21.6617	38.8522	35.3362
Zswim4	19.2172	18.5268	5.94858	6.64178	5.73827	6.03743	2.78657	3.74963
Zswim7	3.94326	4.77939	12.2847	7.76046	13.1272	10.7502	25.8129	16.6109
